# Multifunctional Arylsulfone and Arylsulfonamide-Based Ligands with Prominent Mood-Modulating Activity and Benign Safety Profile, Targeting Neuropsychiatric Symptoms of Dementia

**DOI:** 10.1021/acs.jmedchem.1c00497

**Published:** 2021-08-26

**Authors:** Monika Marcinkowska, Adam Bucki, Joanna Sniecikowska, Agnieszka Zagórska, Nikola Fajkis-Zajączkowska, Agata Siwek, Monika Gluch-Lutwin, Paweł Żmudzki, Magdalena Jastrzebska-Wiesek, Anna Partyka, Anna Wesołowska, Michał Abram, Katarzyna Przejczowska-Pomierny, Agnieszka Cios, Elżbieta Wyska, Kamil Mika, Magdalena Kotańska, Paweł Mierzejewski, Marcin Kolaczkowski

**Affiliations:** † Faculty of Pharmacy, Jagiellonian University Medical College, 9 Medyczna St., 30-688 Krakow, Poland; ‡ Institute of Psychiatry and Neurology, 9 Sobieskiego Street, 02-957 Warsaw, Poland; § Adamed Pharma S.A., 6A Mariana Adamkiewicza Street, Pienkow, 05-152 Czosnow, Poland

## Abstract

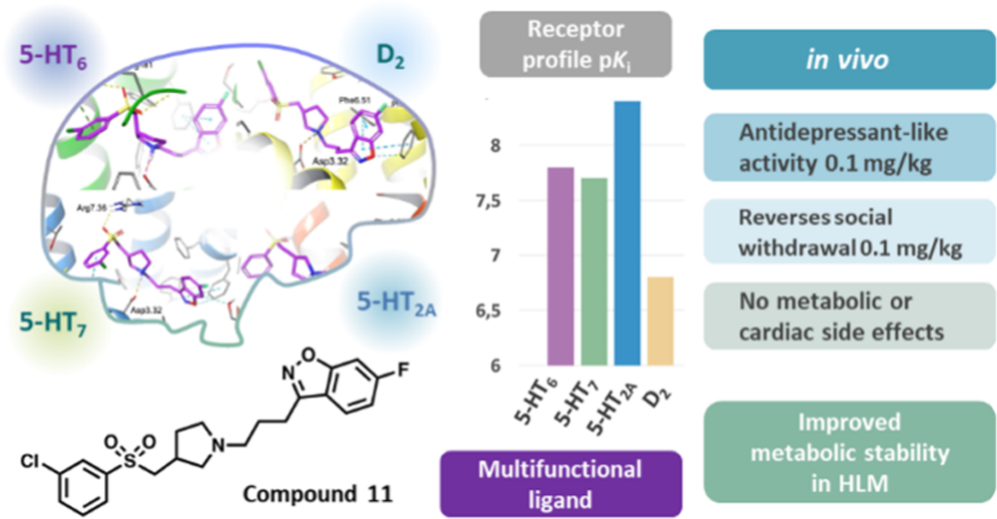

The current pharmaceutical market lacks therapeutic agents designed to modulate behavioral disturbances associated with dementia. To address this unmet medical need, we designed multifunctional ligands characterized by a nanomolar affinity for clinically relevant targets that are associated with the disease pathology, namely, the 5-HT_2A/6/7_ and D_2_ receptors. Compounds that exhibited favorable functional efficacy, water solubility, and metabolic stability were selected for more detailed study. Pharmacological profiling revealed that compound **11** exerted pronounced antidepressant activity (MED 0.1 mg/kg), outperforming commonly available antidepressant drugs, while compound **16** elicited a robust anxiolytic activity (MED 1 mg/kg), exceeding comparator anxiolytics. In contrast to the existing psychotropic agents tested, the novel chemotypes did not negatively impact cognition. At a chronic dose regimen (25 days), **11** did not induce significant metabolic or adverse blood pressure disturbances. These promising therapeutic-like activities and benign safety profiles make the novel chemotypes potential treatment options for dementia patients.

## Introduction

The very first portrait of a dementia patient, described by Alois Alzheimer in 1907, reported an elderly woman who, along with severe cognitive decline, manifested disturbing neuropsychiatric symptoms.^1^ Nowadays, the symptoms of abnormal behavior have been termed as behavioral and psychological symptoms of dementia (BPSD) and are recognized as an inherent element of Alzheimer’s disease (AD) and other types of dementia.^2^ The spectrum of behavioral abnormalities includes agitation, aggression, irritability, apathy, depressive mood, anxiety, psychosis, and reduced sociability. BPSD affects approximately 90% of dementia individuals and is considered to be an even more disturbing symptom in everyday care than memory impairment.^2,3^

The pharmacological management of BPSD poses an enormous challenge to psychiatrists, since specifically designed and approved pharmacotherapies remain inaccessible.^4^ Consequently, the most severe BPSD such as depressive mood, aggression, agitation, and psychosis have had to be addressed with available psychotropic drugs.^5^ Antidepressants have been used in an attempt to treat the depressive mood commonly exhibited by dementia individuals.^6^ However, the effectiveness of these medications remains dubious, as shown by the recent systematic review by Cochrane.^7^ Moreover, treatment with antidepressants poses a risk of side effects that geriatric patients are particularly sensitive to, such as cognitive slowing or cardiac arrhythmias.^8−11^ Clinical practice showed that the use of atypical antipsychotics in the treatment of dementia-related psychosis or agitation/aggression gives modest efficacy at the expense of a high risk of side reactions.^12,13^ Elderly individuals appear to be particularly responsive to antipsychotic-induced metabolic and cardiovascular side reactions as well as cognitive deterioration, which results in significantly higher mortality.^14,15^ In addition, clinicians do not support the routine use of benzodiazepines in dementia patients because these drugs induce excessive daytime sleepiness with resulting cognitive slowing and increased risk of abnormal falls and resulting injuries.^16^

Given the unfavorable status related to the current pharmacotherapy of BPSD, more emphasis should be placed on the development of specifically designed and well-tolerated medications for BPSD. Clinicians accentuate that, to achieve optimal clinical efficacy, BPSD patients should be treated with therapeutic agents that interact with pharmacologically relevant targets, matching the pathogenesis of the disease.^17,18^ BPSD are a result of complex pathological processes induced by neurodegeneration and aging, running in parallel for several years. These processes cause shifts in neurotransmitter abundance, which, in turn, trigger changes in the expression and function of certain receptors.^19^

Genetic studies have made important strides in uncovering the specific receptor changes that contribute to the onset of BPSD. For instance, a robust association between the polymorphism of the serotonin 5-HT_2A_ receptor and the onset of psychosis in AD patients was found.^20,21^ This evidence supports the relevance of targeting the 5-HT_2A_ receptor to mitigate psychotic symptoms in dementia patients. The pertinence of this strategy was confirmed in clinical use, and a 5-HT_2A_ inverse agonist pimavanserin is pending approval for the treatment of dementia-related psychosis.^22^ Another relevant molecular target that closely correlates with the BPSD pathology includes the serotonin 5-HT_6_ receptor. Postmortem studies have revealed a reduced density of 5-HT_6_ receptors in the prefrontal cortex of AD patients who manifested neuropsychiatric symptoms, compared to age-matched healthy controls.^23−25^ The results from animal models showed that pharmacological modulation of 5-HT_6_ receptor activity accounts for anxiolytic, antidepressant, and memory-enhancing properties.^26^ These shreds of evidence validate the 5-HT_6_ receptor as a therapeutic target in managing neuropsychiatric symptoms in dementia patients.

Similar observations were found after pharmacological modulation of another serotonin receptor subtype—5-HT_7_.^27^ In experimental studies, 5-HT_7_ receptor antagonists elicited pronounced antidepressant and anxiolytic-like activity. Reduced levels of serotonin 5-HT_7_ receptors were observed in the brain of aging rodents,^28^ and decreased expression of 5-HT_7_ receptors in the hippocampus was linked to age-related memory impairment.^29^ The dopamine D_2_ receptor acquired considerable importance as a potential target for BPSD after clinical studies revealed changes in the abundance of dopamine D_2_ receptors in AD patients that manifest behavioral abnormalities.^30−32^ Other studies suggested that dysfunction of dopaminergic signaling may result in the onset of apathy and loss of enjoyment in AD patients. Therefore, targeting D_2_ receptors to mitigate behavioral symptoms in dementia patients constitutes a rational intervention.

Given the multifactorial origin of BPSD, acting on an individual target might be insufficient to deliver the desired therapeutic efficacy. In fact, a recent belief that “single drugs do not cure complex diseases” has shifted drug discovery paradigms toward the development of multifunctional drugs, which can hit simultaneously multiple targets.^33,34^ Such a strategy offers a prospect of tackling several biological targets relevant to the disease and, thus, achieve a superior therapeutic window, compared to target-selective therapies. The attractiveness of such an approach inspired us to design multifunctional ligands dedicated to the sensitive population of dementia patients. In the pursuit of a novel anti-BPSD agent, we sought to obtain multifunctional molecules that will modulate the activity of the target combination that underlines BPSD pathology. We identified 5-HT_2A_, 5-HT_6_, 5-HT_7_, and D_2_ receptors as major druggable targets in the development of potential anti-BPSD agents because their dysregulation underlies many pathophysiological conditions associated with BPSD. Herein we report the design, synthesis and comprehensive *in vitro* characterization of a series of novel promising chemotypes based on the arylsulfone and methylopyrrolidine modifications of 6-fluoro-3-[3-(pyrrolidin-1-yl)propyl]benzo[*d*]isoxazole scaffolds that elicit a multifunctional profile in relation to laboratory-based models representing BPSD. To confirm the therapeutic potential of the presented chemotypes, the most promising molecules were comprehensively characterized in extended pharmacological studies. Finally, considering the specific sensitivity of dementia patients to drug-induced adverse reactions, in the final stage, we determined whether these novel compounds exhibit favorable safety effects that would be compatible with the further development of these dual chemotypes as successful anti-BPSD agents.

## Results and Discussion

### Design

**Figure 1:**
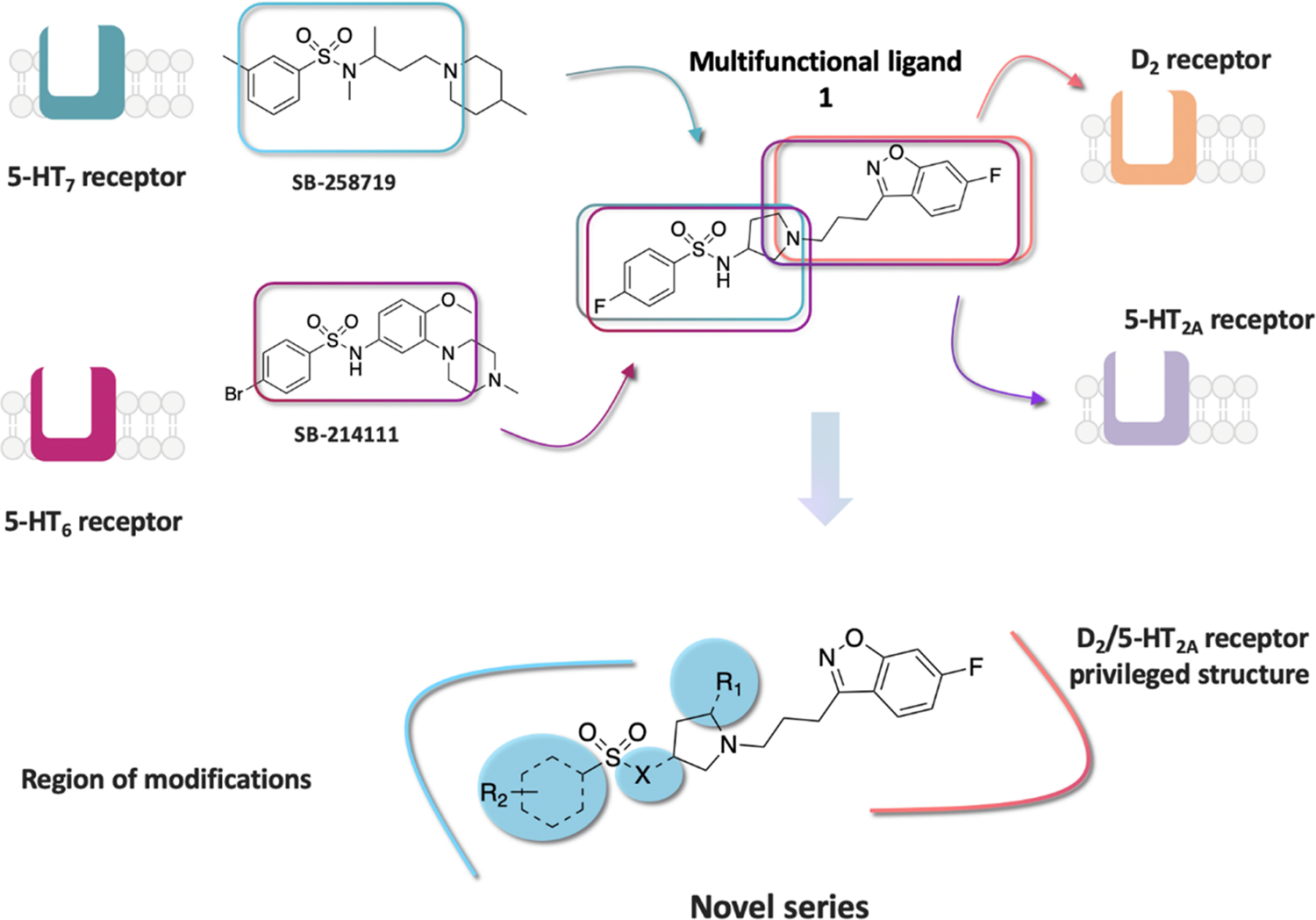
Design strategy based on “the framework combination approach” to deliver multifunctional ligand **1**, containing a central basic nitrogen atom within the overlapping boxes representing the combined scaffolds. Further structural modifications resulted in the novel series based on arylsulfone and methylpyrrolidine fragments.

One of the strategies practiced in pursuit of multifunctional agents is “the framework combination approach”, which takes two distinct pharmacophores and combines them into a single chemical entity that possesses the activity of both original fragments. In this approach, the degree of framework overlap is systematically increased until the frameworks overlap maximally to give the simplest single molecule, with druglike properties. The framework combination strategy has been already successfully applied in drug discovery to develop multifunctional drugs that have been approved for human clinical use.^35^ On the basis of this strategy, we have previously successfully identified several series of druglike multifunctional ligands acting on a defined set of serotonin and dopamine receptors, with a representative molecule **1** portrayed in Figure 1. We found that the superimposition of the selective 5-HT_7_ receptor antagonist, SB 258719, with the potent 5-HT_6_ receptor antagonist, SB214111, showed a remarkable overlap of the pharmacophore features to yield a common *N*-(3-(piperidin-1-yl)propyl)benzenesulfonamide fragment, with molecular recognition for both 5-HT_6_ and 5-HT_7_ receptors. Structure–activity relationship (SAR) studies around this scaffold revealed that the replacement of the piperidine ring with pyrrolidine markedly altered the geometry of the molecule and conferred a balanced 5-HT_2A_/D_2_ receptor functionality to the 6-fluoro-3-(3-(pyrrolidin-1-yl)propyl)benzo[*d*]isoxazole core. We took advantage of the basic nitrogen atom, which is present in both frameworks, and used it to merge both fragments to construct the smallest possible molecule with acceptable physicochemical properties, namely, compound **1**.^36^ Although **1** displayed a well-balanced affinity for the defined biological targets (p*K*_i_ > 7), which encouraged us to further explore this chemotype, it was characterized by high microsomal clearance, requiring the need for possible improvements. We found that all of the molecules in this series, including **1**, underwent metabolic degradation with the predominant transformation routes involving sulfonamide bond cleavage^37^ and pyrrolidine N-dealkylation (Supporting Information Figure S1). Therefore, we reasoned that the metabolic stability of this compound might be rationally tuned by the replacement of the labile sulfonamide function with a bioisosteric sulfone (series I) and the pyrrolidine ring substituted by methylpyrrolidine (series II). The latter modification was to introduce steric hindrance against CYP450-mediated oxidation, thus reducing N-dealkylation.^38^ In the present study, we undertook structural modifications of the core 6-fluoro-3-[3-(pyrrolidin-1-yl)propyl]benzo[*d*]isoxazole moiety, in pursuit of more metabolically stable chemotypes, and verification of how these systemic modifications would affect the receptor profile of the resulting novel compounds (Figure 1).

**Figure 2:**
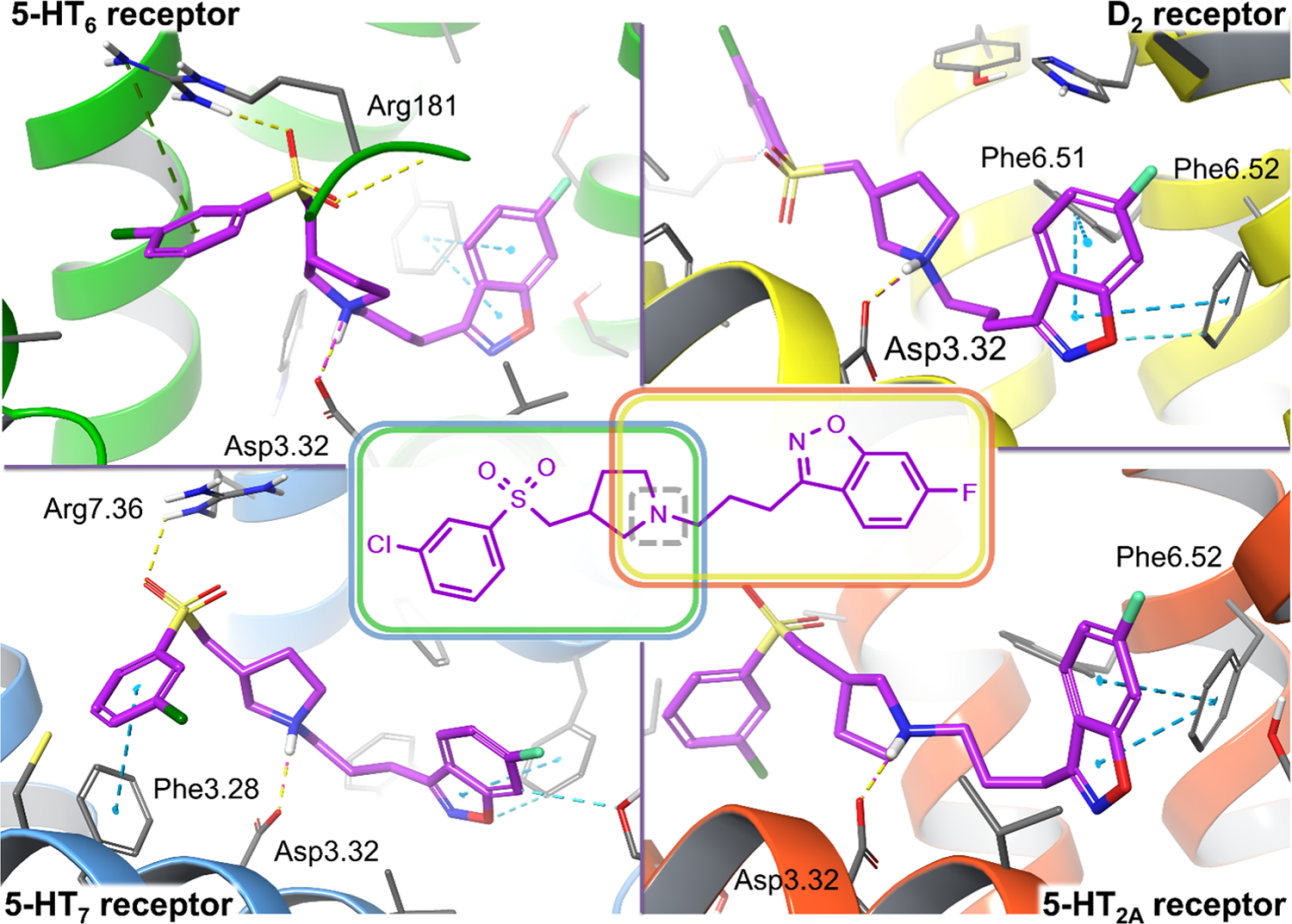
Proposed binding mode of representative compound **11** with the targeted receptors. The arylsulfone fragment substituted with an alkylarylamine moiety satisfied the required interactions for both the 5-HT_7_ and 5-HT_6_ receptor binding sites (homology models based on 2RH1 and 4IAR, respectively), mimicking the interactions of their reference ligands.^41−43^ 6-Fluoro-benzo[*d*]isoxazole linked to the propylamine moiety constitutes a pharmacophore with blocking properties for the 5-HT_2A_ and D_2_ receptors (homology models based on 4IB4 and 3PBL, respectively).^44,45^ The design stage resulted in a series of 21 ligands potentially characterized by high affinity for both the desirable biological targets. Key amino acid residues engaged in ligand binding (within 4 Å from the ligand atoms) are displayed as thick sticks together with their interactions: salt bridges (dotted magenta lines), H-bonds (dotted yellow lines), halogen bonds (dotted violet lines), π–π stacking (dotted cyan lines), or cation−π (dotted green lines). The detailed complexes of lead compounds representing series I (compound **7**)**** and series II (compound **16**) are shown in Supporting Information Figures S2–S5. (For interpretation of the references to color in this figure legend, the reader is referred to the Web version of this article.).

The compounds complied with Lipinski and Veber criteria (except for compounds **19** and **22**, due to exceeded molecular weight, and compound **20**, with exceeded MW and log *P*). The p*K*_a_ values of the most basic nitrogen atom of the compounds did not exceed 8, which limits the risk of poor cell membrane permeability due to ionization issues, or P-gp efflux susceptibility. The series was characterized by promising values of Central Nervous System Multiparameter Optimization (CNS MPO) reaching up to 5.1 (median 4.1). Moreover, none of the compounds contained substructural features recognized as pan assay interference compounds (PAINS, reported by SwissADME). The predicted molecular properties suggested a druglike profile, possibly favorable bioavailability and low risk of attrition of the designed compounds (see Supporting Information Table S1). In addition, we expected that, by changing the substitution pattern around the aryl ring, we could enhance the biological activity profile of the new chemotypes, as arylsulfone moieties have been previously reported to demonstrate an affinity for 5-HT_6_ and 5-HT_7_.^39,40^ The structural changes were monitored using a structure-based molecular modeling approach to predict the binding capabilities of the modified core structures. Docking into the biological targets of interest proved that the binding modes of the designed compounds resembled those of the prototype representatives (Figure 2).^36^ Such an observation suggested that the affinity should remain high, despite the modifications that were primarily made to increase metabolic stability. The chemical structures of the novel series are presented in Tables 1 and 2. In series II, featuring a methylpyrrolidine ring, we decided to investigate the role of the 3S,5S enantiomer, due to the superior docking score for all of the targeted receptors.

**Table 1:**
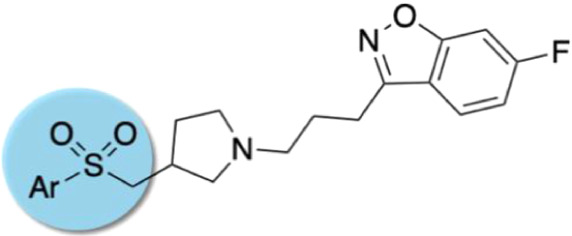

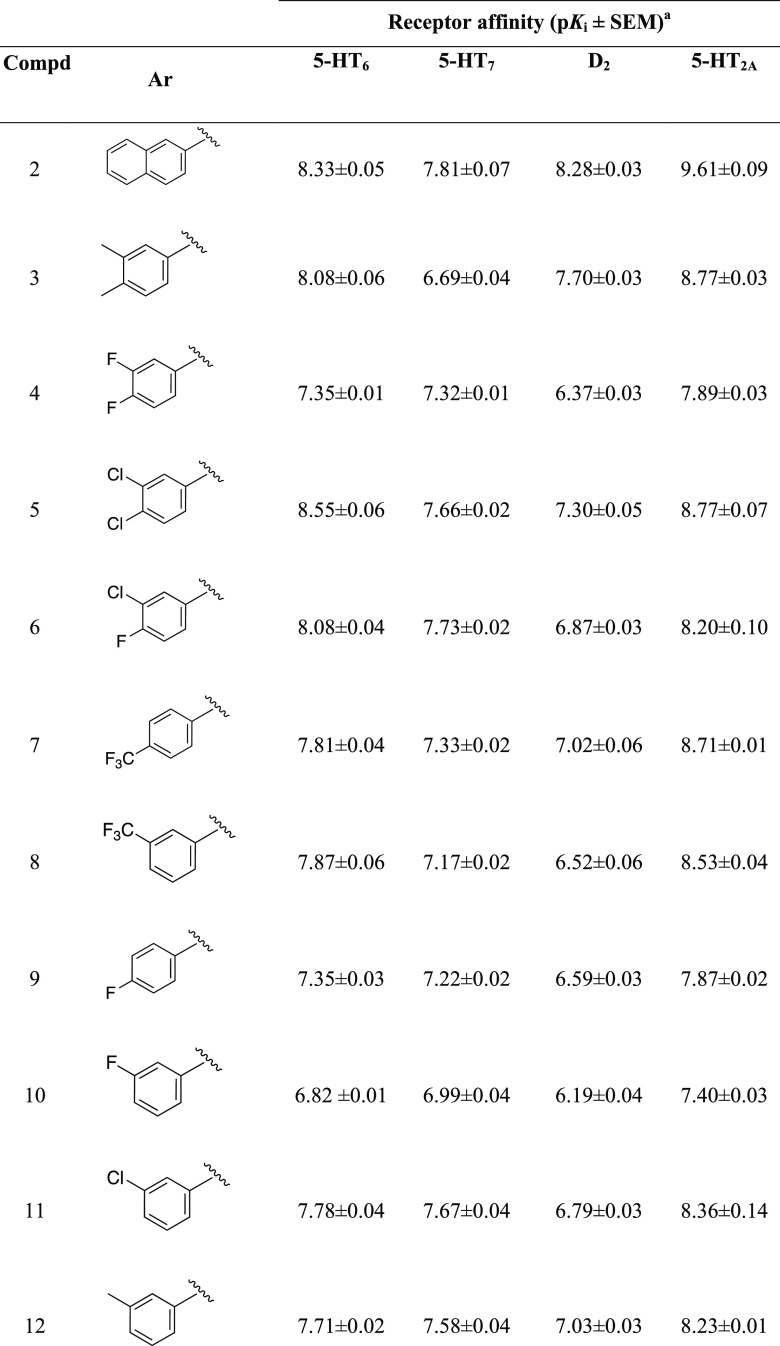

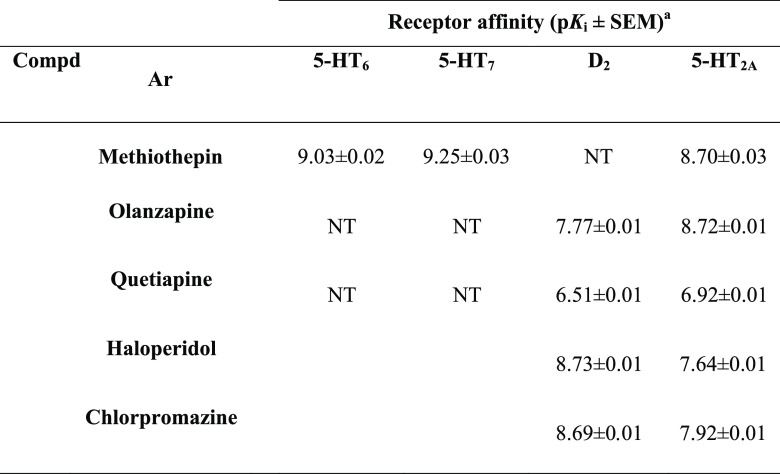
Structure and Binding Affinities for 5-HT_2A_, 5-HT_6_, 5-HT_7_, and D_2_ Receptors of Compounds **2–12** (Series I) and Reference Drugs^a^

a Binding affinity values are represented as p*K*_i_ (i.e., −log *K*_i_) and expressed as mean ± standard error of the mean (SEM) from at least three experiments performed in duplicate.

**Table 2:**
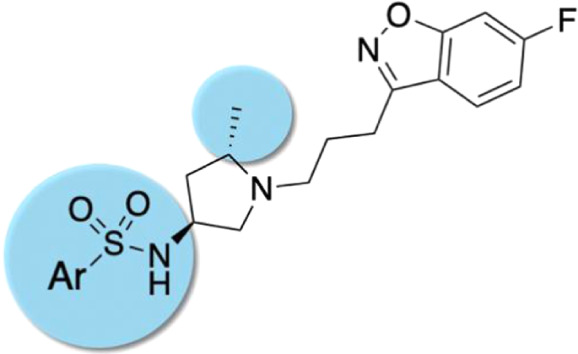

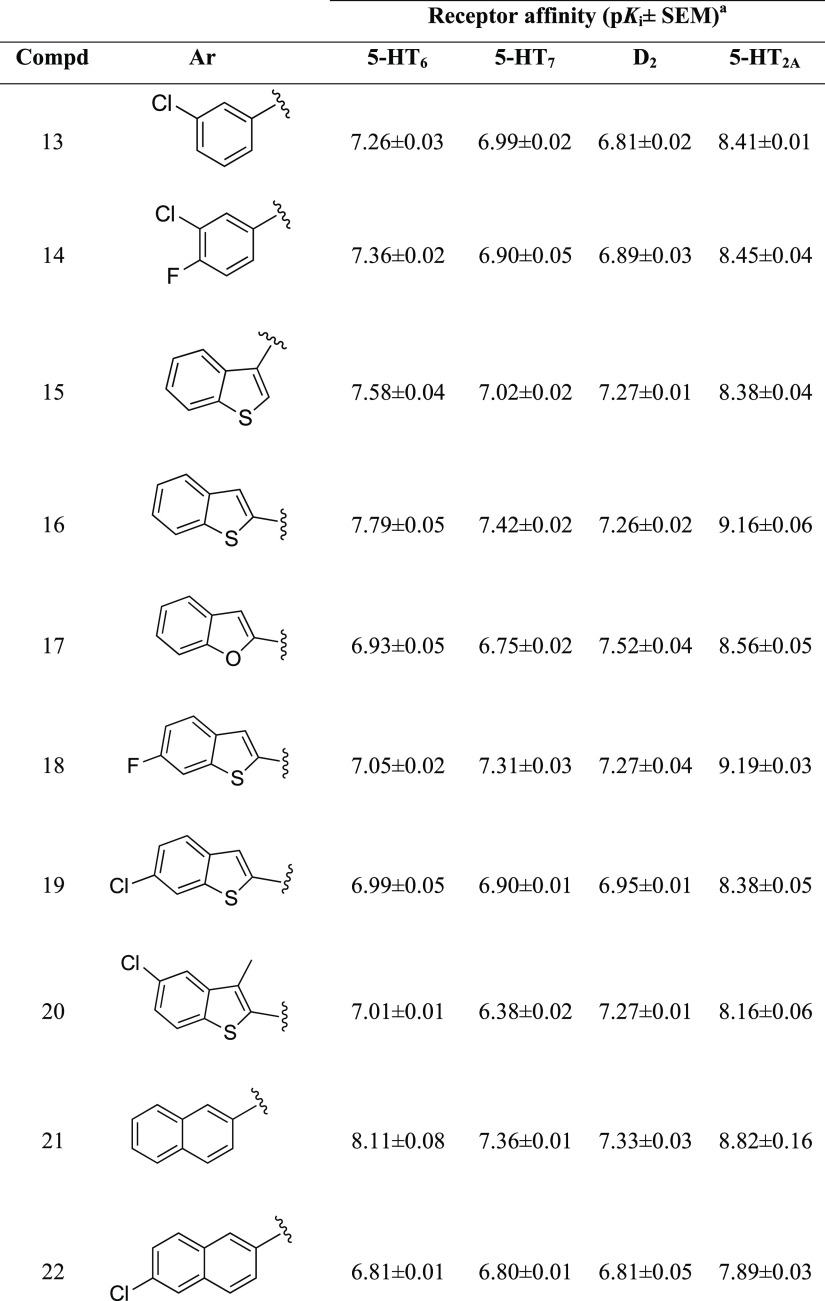

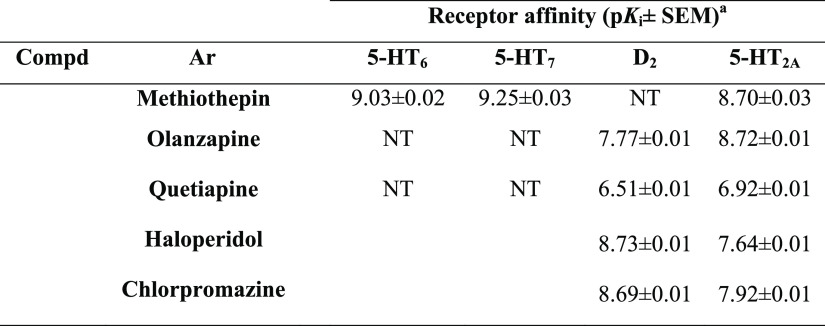
Structure and Binding Affinities for 5-HT_2A_, 5-HT_6_, 5-HT_7_, and D_2_ Receptors of Compounds **13–22** (Series II) and Reference Drugs^a^

a Binding affinity values are represented as p*K*_i_ (i.e., −log* K*_i_) and expressed as mean ± SEM from at least three experiments performed in duplicate.

### Synthesis

**Scheme 1:**
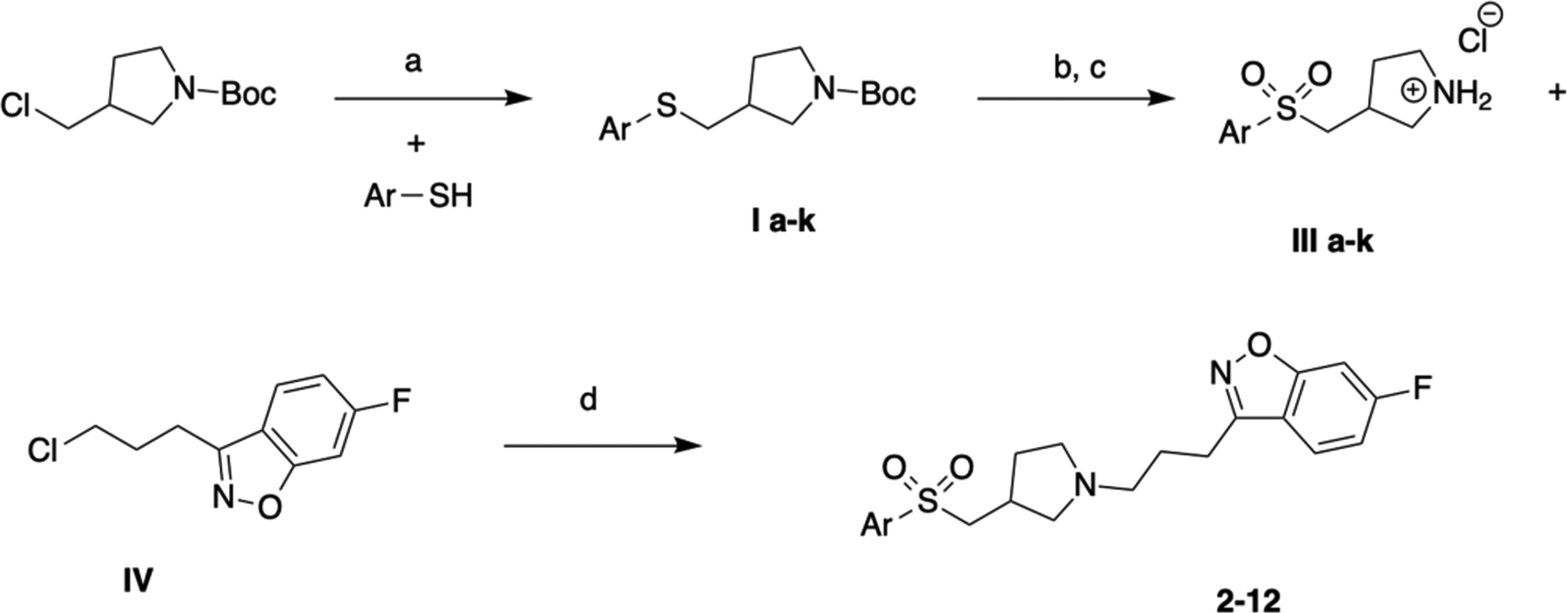
Reagents and Conditions: (a) K_2_CO_3_, EtOH, 80 °C, 2–6 h; (b) *m*CPBA, Dichloromethane (DCM), rt, 1 h then 35 °C, 12 h (c) 1 M HCl in EtOAC, rt, 12 h; (d) K_2_CO_3_, MeCN, KI, 60 °C, 24 h

The series I compounds **2–12** were prepared in a four-step synthesis, as portrayed in Scheme 1. The synthesis commenced with the preparation of key building blocks **III a–k**. The starting thiol derivatives were S-alkylated with *N*-Boc-3-chloromethyl pyrrolidine and then subjected to an oxidation reaction in the presence of *m*-chloroperbenzoic acid (*m*CPBA), to deliver the corresponding sulfone derivatives **II a–k**. The latter were first deprotected with HCl/EtOAc to give key amines **III a–k**, which then reacted with 3-(3-chloropropyl)-6-fluorobenzo[*d*]isoxazole **IV** to yield the final products, **2–12**.

**Scheme 2:**
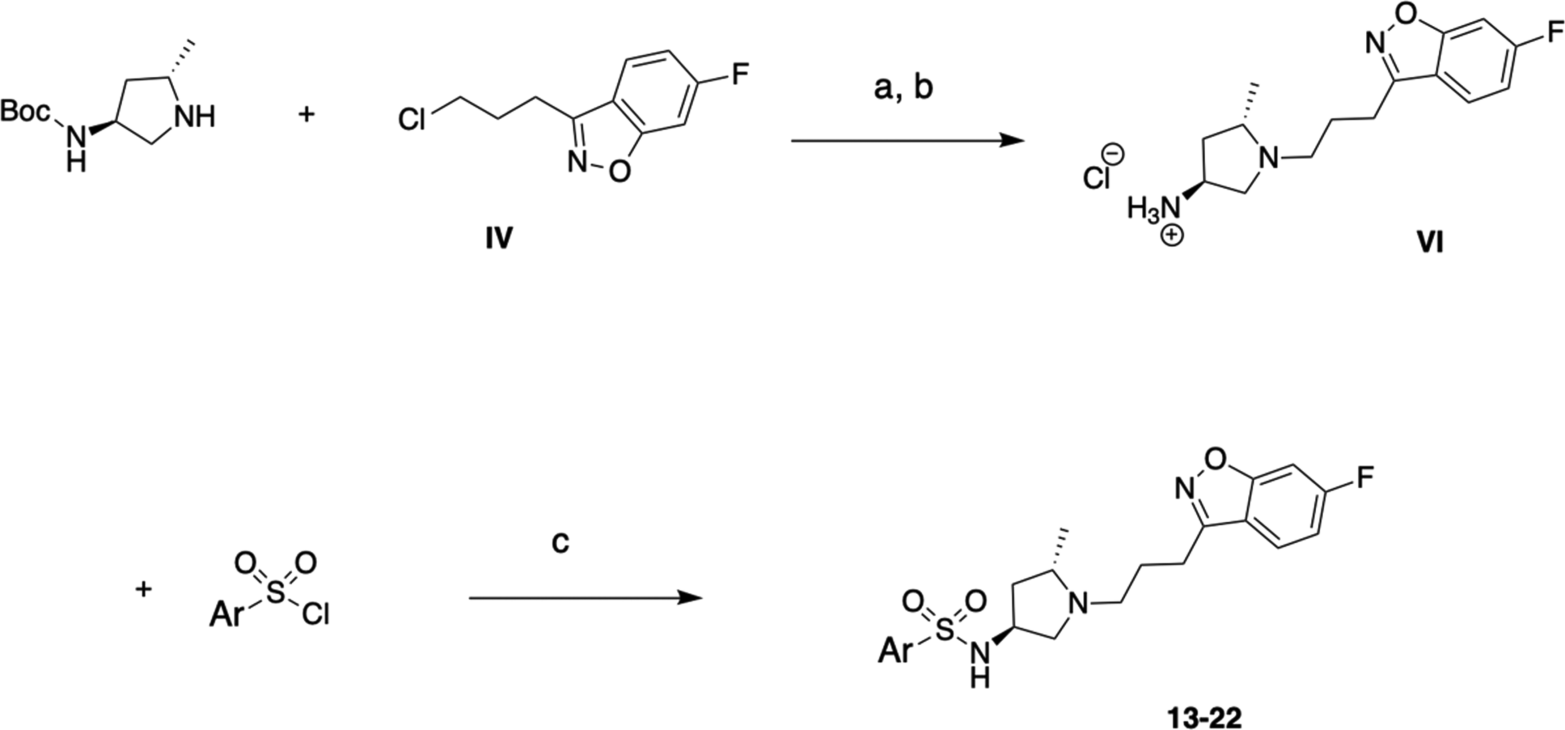
(a) K_2_CO_3_, CH_3_CN, 60 °C, 48 h; (b) HCl/EtOAc 12 h, rt; and (c) Cs_2_CO_3_, 4-Dimethylaminopyridine (DMAP), DCM, rt, 12 h

We next addressed the synthesis of series II, namely, Compounds **13–22** (Scheme 2). The key intermediate **VI** was constructued from commercially available 5-methylpyrrolidin-3-amine and 3-(3-chloropropyl)-6-fluorobenzo[*d*]isoxazole **IV**, which were first reacted in the presence of K_2_CO_3_ and KI to give an intermediate, *tert*-butyl (1-(3-(6-fluorobenzo[*d*]isoxazol-3-yl)propyl)-5-methylpyrrolidin-3-yl)carbamate, **V**. Next, the removal of Boc protecting moiety under acidic conditions yielded **VI**, which was further alkylated with corresponding sulfonyl chlorides to produce the desired molecules, Compounds **13–22**.

### Structure–Activity Relationships

#### Series I

All of the synthesized compounds were characterized in radioligand binding assays to establish their affinity for target serotonin 5-HT_2A_, 5-HT_6_, 5-HT_7_, and dopamine D_2_ receptors (Tables 1 and 2). The vast majority of compounds performed well in the *in vitro* assays and displayed marked affinities for all of the desired receptors, with p*K*_i_ values ranging from 6.19 to 9.61. It is noteworthy that we observed a favorable difference between the p*K*_i_ for the 5-HT_2A_ receptor and the p*K*_i_ for the D_2_ receptor for all compounds, within the range of 1–1.5, which translates to a 10-fold to 100-fold preference for the 5-HT_2A_ receptor. This testifies its promising safety, particularly in terms of the appearance of potential extrapyramidal side effects. The potent 5-HT_2A_ binding capabilities were proven for a majority of the sulfone derivatives, with p*K*_i_ values ranging between 7.40-the 5-HT_6_, 5-HT_7_, and dopamine D_2_ receptors varied depending on the structure of the aryl fragment. Our first attempts focused on assessing the impact of various substituents incorporated into the meta position of the phenyl ring on the resulting interactions with 5-HT_6_/5-HT_7_ and D_2_ receptors. The affinity values were compared to the derivative containing a 3-Cl benzyl function, which was well tolerated by all targets (p*K*_i5-HT6_ = 7.78, p*K*_i5-HT7_ = 7.67, p*K*_iD2_ = 6.79, p*K*_i5-HT2A_ = 8.36), and, based on these studies, **11** was chosen as the lead structure for this series.

In series I, we observed that the replacement of the chlorine atom at the meta position with a methyl group (compound **12**) resulted in a negligible change in affinity for all tested receptors. On the other hand, introducing a fluorine atom at the meta position (compound **10**) led to a decrease in activity for all targets, which was the most prominent for the 5-HT_6_ and 5-HT_2A_ receptors (p*K*_i5-HT6_ = 6.82 and p*K*_i5-HT2A_ = 7.40 vs p*K*_i5-HT6_ = 7.78 and p*K*_i5-HT2A_ = 8.36 for **11**).

Incorporation of a CF_3_ substituent at the meta position of the phenyl ring (compound **8**) resulted in reduced affinity for 5-HT_7_ (p*K*_i_ = 7.17) and D_2_ (p*K*_i_ = 6.52) receptors, with no substantial changes toward the 5-HT_6_ and 5-HT_2A_ receptors. Shifting the CF_3_ group from the meta to para position (compound **7**) slightly improved affinity for the D_2_, 5-HT_2A_, and 5-HT_7_ receptors (p*K*_i_ = 7.02, 8.71, 7.33, respectively), with no influence on the 5-HT_6_ receptor (p*K*_i_ = 7.81). Compared to lead structure **11**, the *para* CF3 derivative **7** maintained a high affinity for the 5-HT_6_ receptor, a slightly improved affinity for D_2_ (p*K*_i_ = 7.02) and 5-HT_2A_ (p*K*_i_ = 8.71) receptors, but showed a small decrease in affinity for the 5-HT_7_ receptor (p*K*_i_ = 7.33). Introducing a fluorine atom at the para position (**9**) led to a slight decrease in affinity for all targets, in comparison to **11**.

Further studies focused on the exploration of different types of 3,4-disubstituted derivatives. The introduction of a second chlorine atom at the para position was well tolerated and compound **5**, featuring a 3,4-dichlorophenyl group, showed high affinity for all receptor targets (p*K*_i_ = 7.30–8.77), comparable with the mono-substituted compound, **11**. In the case of the 3-chloro-4-fluorophenyl derivative, **6**, no substantial changes in affinity were observed. Compound **4**, featuring a 3,4-difluorophenyl group, maintains almost the same level of activity as its mono-substituted *para* analogue, **9**. In comparison with the 3-methylphenyl derivative, **12**, incorporation of a second methyl group at the 4-position of the phenyl ring (compound **3**, 3,4-dimethylphenyl derivative) generally improved affinity for all targets, with exception of the 5-HT_7_ receptor, where a marked drop in affinity was observed (p*K*_i_ = 6.69). Replacing the phenyl ring with a naphthyl ring (compound **2**) maintained a high affinity for all targets (p*K*_i_ = 7.81–9.61), and was particularly favorable for the 5-HT_2A_ and 5-HT_6_ receptors (Table 1).

#### Series II

Further SAR studies revealed that the methylpyrrolidine series exhibited a high affinity for the 5-HT_2A_ receptor with p*K*_i_ values in the range of 7.89–9.19, while the affinity for the remaining receptors varied depending on the aryl fragment (Table 2). The affinities of the methylpyrrolidine series were compared to the 3-Cl benzyl derivative, **13**, which was established as a starting point. Introduction of the 3-chloro-4-fluorophenyl ring, resulting in compound **14**, maintained a high affinity for all targets, and p*K*_i_ values were comparable to the mono-substituted analogue, **13**. Replacement of the phenyl ring with 3-benzothiophene (**15**) or 2-benzothiophene (**16**) improved affinity for the 5-HT_6_ receptor (p*K*_i_ = 7.58 and 7.79 respectively) and the D_2_ receptor (p*K*_i_ = 7.27 and 7.26). In the case of 2-benzothiophene (**16**), we noticed also an improvement in binding affinity for the 5-HT_2A_ receptor, with a p*K*_i_ value of 9.16. Incorporation of the 2-benzofuran ring (**17**) slightly reduced affinities for the 5-HT_6_ (p*K*_i_ = 6.93) and 5-HT_7_ (p*K*_i_ = 6.75) receptors, while the activity against the D_2_ receptor increased (p*K*_i_ = 7.52), compared with **13**. Introduction of a 6-fluoro-2-benzothiophene ring (compound **18**), improved interaction with 5-HT_2A_ (p*K*_i_ = 9.19), 5-HT7 (p*K*_i_ = 7.31) and D_2_ (pKi = 7.27) receptors, while a slight reduction was observed in the affinity for the 5-HT_6_ receptor (p*K*_i_ = 7.05). Swapping the fluorine atom (compound **18**) for chlorine at the 6-position of the 2-benzothiophene ring in compound **19** resulted in a substantial decrease in affinity for the 5-HT_2A_ receptor (p*K*_i_ = 8.38), but a slight decrease in affinity for 5-HT_7_ (p*K*_i_ = 6.90), D_2_ (p*K*_i_ = 6.95), and 5-HT_6_ (p*K*_i_ = 6.99) receptors. Similar activities were observed for compound **20** (5-chloro-3-methyl-1-benzothiophene derivative). When the phenyl ring was replaced with a 2-naphthyl ring, compound **21**, affinities for all receptors increased. However, placing an additional chlorine atom in the naphthyl ring (**22**) resulted in a considerable decrease in affinity for all targets. Based on the above results, we observed that unsubstituted heterocycles make the compounds more favorable for achieving proportionate interactions with all four receptors (Table 2).

### *In Vitro* Functional Activity

The most promising compounds, **2**, **3**, **5**, **7**, **11**, **15**, **16**, **18**, and **21** were subsequently submitted to functional studies. The majority of compounds exerted a prominent antagonist effect against all targeted receptors with nanomolar *K*_B_ values (p*K*_B_ 6.60–8.30) (Table 3) The sulfone derivatives (series I) performed slightly better than the methylpyrrolidine derivatives (series II) and a majority of compounds from series I demonstrated *K*_B_ values < 100 nM (p*K*_B_ > 7). Among the methylpyrrolidine derivatives, compounds bearing 3-benzothiophene (**15**), 2-benzothiophene (**16**), and 2-naphthyl (**21**) demonstrated the most efficacious antagonistic responses, with p*K*_B_ values > 6.34.

**Table 3:** Functional Data for the Selected Compounds^b^

	antagonist effect p*K*_B_ ± SEM			
compd	5-HT_6_R	5-HT_7_R	D_2_R	5-HT_2A_R
**2**	7.26 ± 0.01	7.06 ± 0.02	7.62 ± 0.01	8.22 ± 0.20
**3**	7.02 ± 0.01	6.85 ± 0.01	7.81 ± 0.01	8.40 ± 0.34
**5**	7.41 ± 0.02	7.26 ± 0.01	7.22 ± 0.02	7.96 ± 0.04
**7**	7.44 ± 0.03	7.42 ± 0.03	8.21 ± 0.01	7.99 ± 0.07
**11**	7.12 ± 0.01	7.36 ± 0.01	7.34 ± 0.03	7.81 ± 0.58
**15**	6.82 ± 0.02	7.12 ± 0.01	6.87 ± 0.01	6.82 ± 0.01
**16**	6.74 ± 0.05	6.40 ± 0.01	6.73 ± 0.02	7.79 ± 0.58
**18**	6.41 ± 0.02	5.62 ± 0.01	6.79 ± 0.01	8.30 ± 0.19
**21**	6.77 ± 0.07	6.34 ± 0.02	6.60 ± 0.02	7.63 ± 0.01
**SB-42457**	9.47 ± 0.01			
**SB-269970**		9.7 ± 0.01		
**Chlorpromazine**			8.80 ± 0.0	
**Pimavanserin**				9.17 ± 0.01

a All of the functional activity values were expressed as mean from at least three experiments performed in duplicate.

b Blank spaces—compounds not tested in the assays.

**Table 4:** Off-Target Activity Data for the Selected Molecules

	% activity at 1.0 × 10^–6^ M^a^			
compd	H_1_R (%)	α_1_R (%)	M_1_R (%)	hERG^b^ (%)
**2**	39	48	–15	23
**3**	58	66	5	34
**5**	54	39	4	31
**7**	29	56	–7	28
**11**	24	43	–5	29
**15**	10	13	11	29
**16**	2	14	–1	40
**18**	19	19	7	38
**21**	4	15	–6	47
**Phentolamine**	91	76		
**Mepyramine**	95		55	
**Amitriptyline**	99	82	78	
**Doxepin**	97		92	
**Quetiapine**	76	75	88	
**Olanzapine**	89	82	89	
**Verapamil**				91

a % inhibition of control specific binding at the concentration of 1.0 × 10^–6^ M. Assays carried out in duplicate (*n* = 2). Blank spaces—compounds not tested in the assay.

b % inhibition of hERG-mediated potassium currents at a concentration of 1.0 × 10^–6^ M.

### Determination of Interaction with Off-Target Receptors

It is becoming increasingly evident that dementia patients are acutely sensitive to drug-induced adverse reactions and show a higher prevalence of metabolic syndrome, stroke, and related conditions caused by psychotropic medications (antidepressants and antipsychotics in particular).^46,47^ The results from population-based studies clearly demonstrate that these undesirable events result in higher mortality among the geriatric dementia population. Higher occurrence of drug-induced side effects has been closely related to psychotropic drugs that exert high affinity for off-targets, such as the histamine (H_1_), muscarinic (M_1_), and α_1_ adrenergic receptors (e.g., imipramine, amitriptyline, doxepin, or antipsychotics: quetiapine and olanzapine).^48^ Small molecules interacting with histamine H_1_ receptors have been linked with drug-induced metabolic disturbances, weight gain, and excessive daytime sleepiness.^49,50^ Moreover, clinical studies found an association between high-H_1_ affinity antidepressants (e.g., amitriptyline, doxepin) and dysregulation of glucose homeostasis, resulting in hyperglycemia.^51^ Blockade of muscarinic M_1_ receptors may induce tachycardia, which drives precarious hemodynamic conditions, changes blood flow in intraparenchymal microcirculation which, in turn, can increase the risk of stroke (olanzapine and quetiapine).^52,53^ Furthermore, blockade of M_1_ receptors escalates the cholinergic deficit and intensifies memory impairment in dementia subjects who suffer from cognitive deterioration. Anticholinergic activity generates additional unpleasant reactions, namely, severe constipation and urine retention, which adds another layer of undesirable effects. Lastly, treating dementia subjects with antidepressants or antipsychotics may trigger orthostatic hypotension, which has been closely related to the blockade of the adrenergic α_1_ receptor. Consequently, it has been suggested that potential anti-BPSD agents should be devoid of any interactions with adverse targets, in particular: histamine H_1_, muscarinic M_1_, and adrenergic α_1_ receptors.^54^

Considering the above, we conducted *in vitro* screening to verify potential interactions of the two series of compounds with the above-mentioned undesirable targets. The two series of compounds proved to elicit a less disruptive profile than the reference antidepressants and antipsychotics, exhibiting substantially lower values of % inhibition of control specific binding at a concentration of 1.0 × 10^–6^ M, compared to the off-targets (Table 4). The two series of compounds showed an almost negligible binding with muscarinic M_1_ receptors. This observation is in line with our previous studies which show that arylsulfonamide-based hybrids bind poorly to muscarinic M_1_ receptors, due to insufficient space within the receptor binding site.^45^ However, we observed a slight tendency toward binding with the H_1_ and α_1_ receptors, particularly among the sulfone series of compounds 2, 3, 5, 7, 11 (α_1_: 43-66% at 1.0 × 10^–6^ M, H_1_: 24–58% at 1.0 × 10^–6^ M), but this was still substantially lower than that of the reference drugs (α_1_: 75–82% at 1.0 × 10^–6^ M, H_1_: 75–99%). This could be attributed to the presence of an aryl fragment featured with a basic pyrrolidine in these compounds, a common pharmacophore shared with other monoaminergic receptors. In general, an increased tendency to interact with α_1_ and H_1_ receptors was observed for the 3,4-disubstituted analogues, **3** and **5**. Interestingly, the methylpyrrolidine series (**15**, **16**, **18**, and **21**) did not show a significant affinity for the H_1_ or α_1_ receptors, indicating that the methyl group around the pyrrolidine moiety impairs H_1_ and α_1_ receptor binding. In summary, the majority of compounds proved to have a relatively high safety window, showing negligible interaction with the M_1_ receptor. In the case of the methylpyrrolidine series, we observed no significant affinity for the H_1_ and α_1_ receptors, while the sulfone series showed a limited interaction with H_1_ and α_1_ receptors. These promising biological activities permitted us to consider all of the compounds as eligible for further *in vitro* profiling.

Additionally, we established the potency of selected compounds to block the human hERG potassium channel, which is the most common mechanism related to drug-induced cardiotoxicity, via prolongation of the QT interval. The compounds were tested in a whole-cell electrophysiological assay at a single concentration of 1.0 × 10^–6^ M, and induced inhibition of hERG in the range of 20–47%. This would suggest that these compounds would display a relatively low occurrence of cardiac side effects.

### Developability Analysis—*In Vitro* Assays

In the present study, our goal was to develop metabolically stable compounds from within the series I and II compound syntheses. We also paid close attention to the physicochemical properties by measuring the thermodynamic solubility of selected compounds.^55^ In general, sulfone analogues were characterized by superior solubility, ranging from 62 to 306 μg/mL (Table 5). Two compounds, **11** and **7**, displayed remarkable solubilities (306.29 and 204.75 μg/mL, respectively), exceeding the reference drug metoprolol (193 μg/mL). Benzothiophene derivatives, **16** and **18**, showed acceptable water solubility, while the other two **15**, **21** were markedly less soluble (<0.1 μg/mL), and with a high probability that they would require more complex formulations for *in vivo* use. Therefore, compounds X and Y were excluded from further development. At this point, we selected the most promising compounds from series I namely: **2**, **7**, **11**, and two methylpyrrolidine derivatives from series II, namely, **16** and **18**. The compound selection was guided by the overall *in vitro* properties of potency and efficacy, as well as aqueous solubility.

**Table 5:** Thermodynamic Solubility Data of Selected Compounds

compd^a^	aqueous solubility μg/mL
**2**	128.38 ± 1.61
**3**	132.7 ± 0.74
**5**	62.44 ± 1.05
**7**	204.75 ± 1.68
**11**	306.29 ± 0.80
**15**	<0.1
**16**	1.62 ± 0.1
**18**	9.16 ± 0.42
**21**	<0.1
**Metoprolol**	193 ± 1.02

a Prior to the measurement, compounds were converted to the corresponding hydrochloride salts.

**Figure 3:**
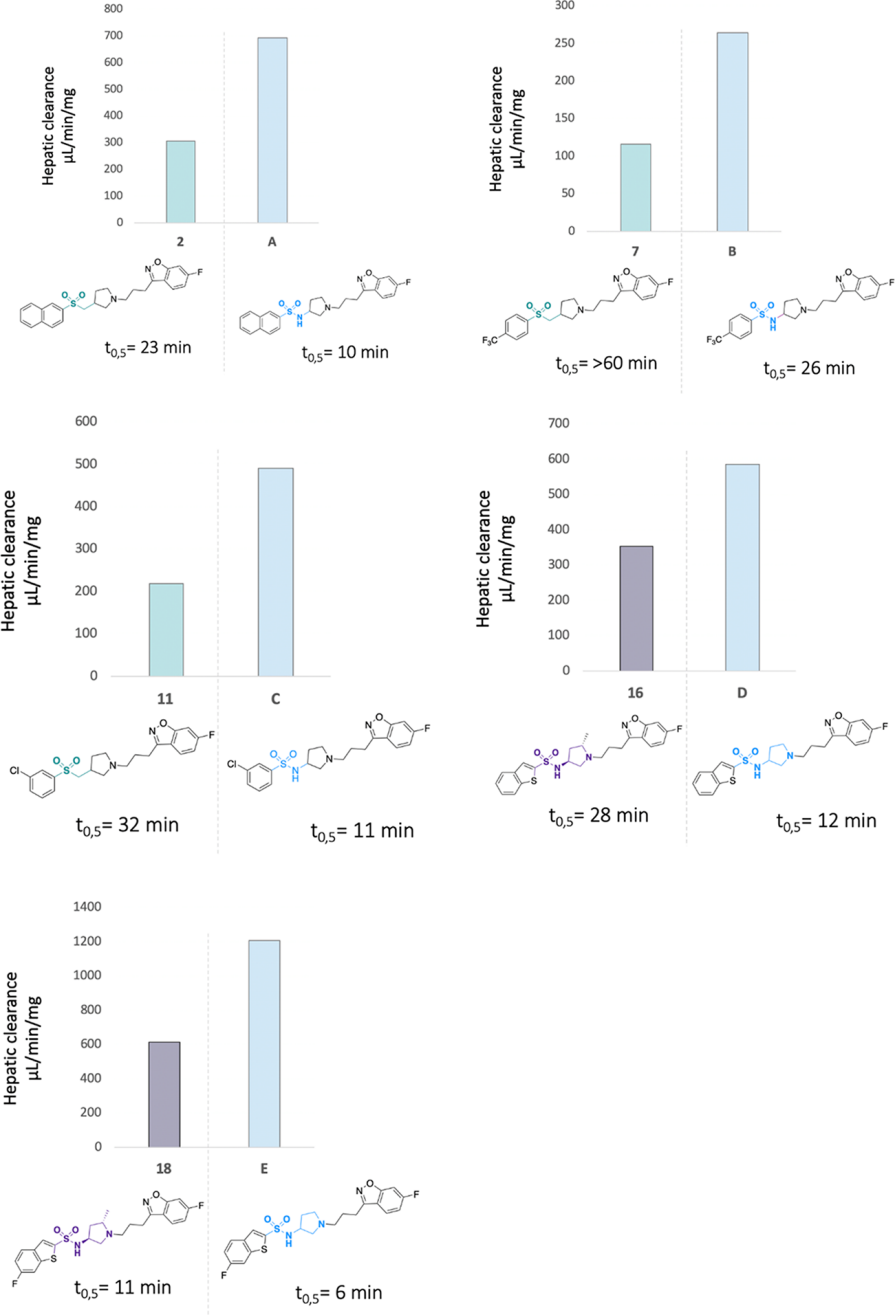
Metabolic stability of selected compounds in human liver microsomes. The study was performed at a concentration of 1.0 × 10^–7^ M. Determination of % of compound recovered after selected periods of time (0, 5, 15, 30, 45, and 60 min) was measured according to previously published protocols.^56^ Internal references: verapamil: *t*_0.5_ = 26 min, CL_int_ = 267 mL/min/mg, imipramine: *t*_0.5_ = >60 min, CL_int_ = <115.5 mL/min/mg.

The selected compounds, **2**, **7**, **11**, **16**, and **18**, were subsequently tested for metabolic stability using human liver microsomes. We determined the quality of these compounds by comparing them head-to-head with the previously developed sulfonamide counterparts.^36^ In general, the derivatives were less heavily metabolized than the parent sulfonamides (Figure 3). This was particularly true for the sulfone derivatives, which showed very promising biological results and were characterized by increased metabolic stability. The highest stability was observed for compound **7**, bearing a 4-CF_3_-phenyl moiety, which displayed desirable values, including a half-life of over 60 min and a hepatic clearance value below 115 μL/(min mg). The remaining sulfone derivatives composed of a naphthyl ring (compound **2**) and a 3-chlorophenyl substituent (compound **11**), also displayed a substantial increase in stability over the parent compounds, **A** and **C**, and half-life increased almost 2-fold, along with improved clearance values (CL_int_ = 304.3 μL/(min mg) for **2** and CL_int_ = 217.3 μL/(min mg) for **11**). Among the methylpyrrolidine series, compound **16**, bearing the 2-benzothiophene motif, showed prominent improvement of stability, and its clearance value was 2-fold lower (CL_int_ = 252.1 μL/(min mg)) in comparison to its sulfonamide analogue, **D** (CL_int_ = 584.5 μL/(min mg)). The addition of one fluorine atom into the 2-benzothiophene ring gave a modest improvement of stability, as compound **18** showed a half-life of only 11 min, with a metabolic clearance CL_int_ = 610.4 μL/(min mg). Although these values were improved in comparison to the corresponding sulfonamide analogue, **E** (CL_int_ = 1204.3 μL/(min mg)), compound **18** remained heavily metabolized and, consequently, **18** was ruled out from further evaluation.

Altogether, the above findings indicate that chemical modifications of the compounds aimed at blocking the predominant metabolic pathways, namely, sulfonamide bond cleavage and pyrrolidine N-dealkylation, resulted in a reduction of microsomal turnover. At this point of the study, compounds **7** and **11** emerged as the most promising compounds for further evaluation, because of their markedly superior metabolic stability. We decided also to include a methylpyrrolidine representative, compound **16**, and explore it further.

### *In Vivo* Studies: Pharmacokinetics

**Figure 4:**
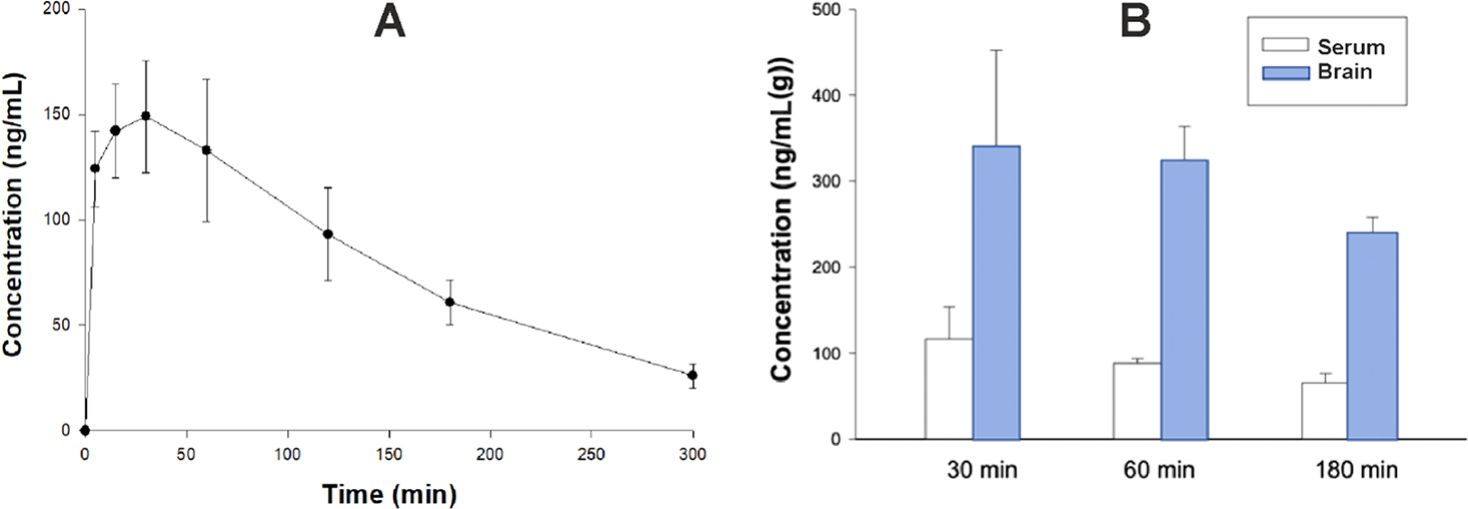
A. Time course of plasma concentrations (mean ± SD) of compound **7** administered intraperitoneally at a dose of 2 mg/kg to male Wistar rats (*n* = 3 per time point). B. Serum and brain concentrations of **7** administered at a dose of 2 mg/kg i.p. (*n* = 3).

**Figure 5:**
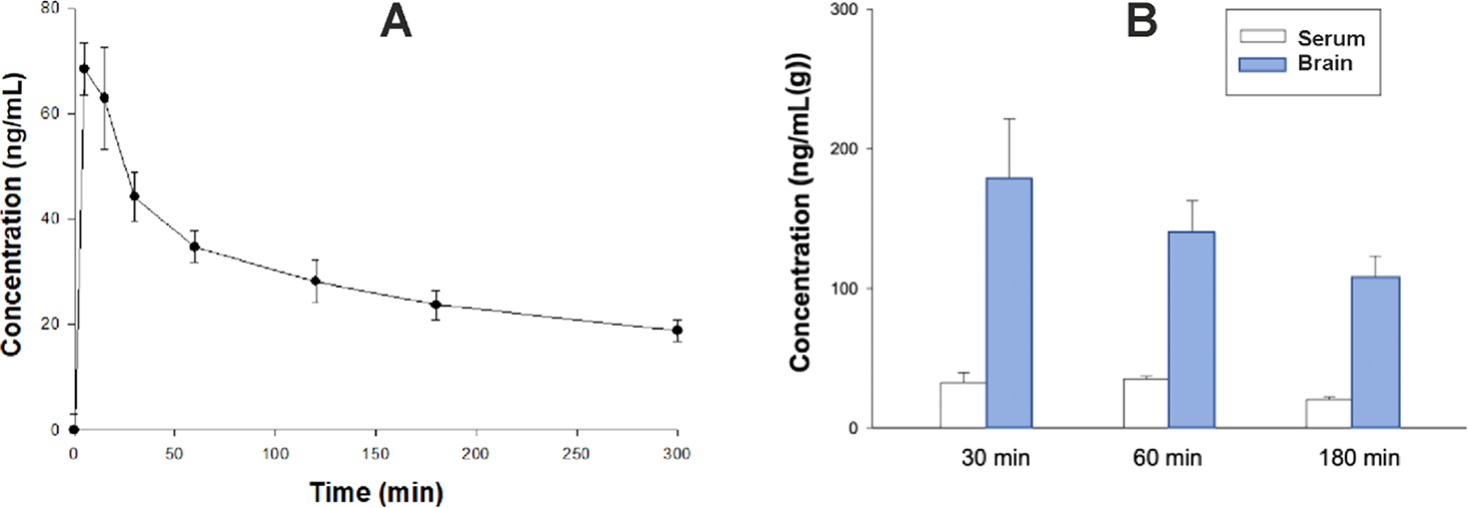
A. Time course of plasma concentrations (mean ± SD) of compound **11** administered intraperitoneally at a dose of 2 mg/kg to male Wistar rats (*n* = 3–4 per time point). B. Serum and brain concentrations of **11** administered at a dose of 2 mg/kg i.p. (*n* = 3).

**Figure 6:**
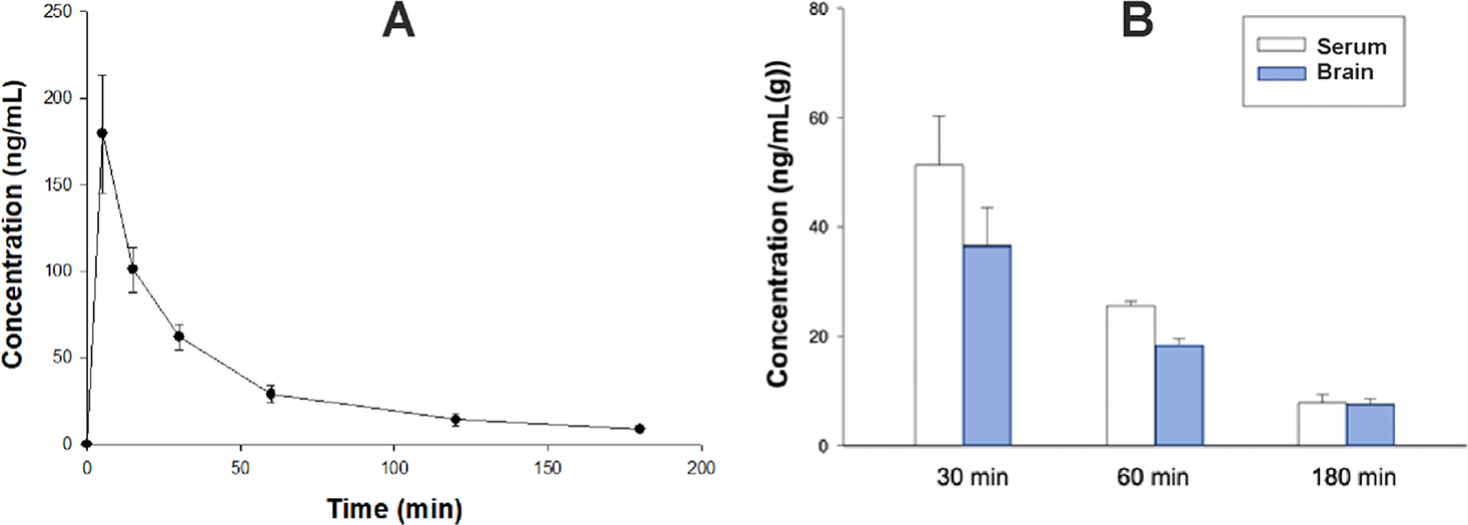
A. Time course of plasma concentration (mean ± SD) of compound **16** following i.p. administration of a dose of 2 mg/kg to rats (*n* = 3 per time point). B. Serum and brain concentrations of **16** administered at a dose of 2 mg/kg i.p. (*n* = 3).

The pharmacokinetic (PK) properties of key molecules, **7**, **11**, and **16**, were assessed *in vivo*. Plasma concentration values versus time were examined by a noncompartmental approach. Figures 4A, 5A, and 6A show that all of the studied compounds administered at a dose of 2 mg/kg i.p. were rapidly absorbed from the peritoneal cavity (*t*_max_ 5.0–22.5 min). Peak plasma concentrations of **7** and **16** were relatively high (*C*_max_ = 193.44 and 179.33 ng/mL, respectively), while *C*_max_ of **11** was lower (*C*_max_ = 69.68 ng/mL). All of the tested compounds were characterized by a slow terminal elimination, resulting in a favorable value for serum elimination half-time (*t*_0.5λz_ = 318.59 min for **11**, 96.09 min for **7**, and 55.67 min for **16**). The area under the concentration–time curve from the time of dosing to infinity (AUC_0–∞_) for serum was 36 269.95 ng·min/mL for **7**, 17 368.37 ng·min/mL for **11** and 7060.67 ng·min/mL for **16**. The apparent volume of distribution (*V*_z_/*F*) during the terminal phase was 7.96 L/kg for **7**, 52.99 L/kg for **11**, and 23.04 L/kg for **16**. Clearance (Cl/F) was 0.12 L/min for **11**, 0.06 L/min for **7** and 0.29 L/min for **16** (Table 6, Figures 4A, 5A, and 6A). Notably, the concentrations of sulfone derivatives **7** and **11** in brain tissue were particularly high, reaching up to 333.65 and 178.32 ng/g after 30 min, respectively (Figures 4B and 5B). The concentrations determined in plasma at this point ranged between 32.23 and 116.43 ng/mL. In the case of the methylpyrrolidine derivative, **16**, the plasma concentrations were in the mid-range of 51.35 ng/mL, whereas the brain concentrations were relatively low (36.62 ng/g), 30 min after administration (Figure 6B). Both the sulfone derivatives maintained high brain concentrations 180 min after administration (between 107.72 and 232.26 ng/mL), suggesting superior bioavailability and metabolic stability, compared to the corresponding parameter for the methylpyrrolidine derivative, which was noticeably lower (7.67 ng/g) (Figures 4B, 5B, and 6B). Collectively, these results suggest that all compounds displayed favorable druglike pharmacokinetic properties in the rat, which prompted their pharmacodynamics evaluation.

**Table 6:** Pharmacokinetic Parameters (Mean ± Standard Deviation (SD)) of the Studied Compounds Administered to Rats^a^ at a Dose of 2 mg/kg i.p. Using Noncompartment Analysis

parameter	**7**	**11**	**16**
*t*_max_ (min)	22.5 ± 10.61	8.3 ± 5.77	5.0 ± 0.00
*C*_max_ (ng/mL)	193.44 ± 49.76	69.68 ± 5.86	179.33 ± 34.11
_λz_ (min^–1^)	0.007 ± 0.0003	0.002 ± 0.0002	0.013 ± 0.0009
*t*_0.5λz_ (min)	96.09 ± 4.25	318.59 ± 26.46	55.67 ± 4.09
AUC_inf_ (ng·min/mL)	36 269.95 ± 8836.54	17 368.37 ± 1680.24	7060.67 ± 1056.47
*V*_z_/*F* (L/kg)	7.96 ± 2.34	52.99 ± 6.05	23.04 ± 3.21
CL/F (L/(min kg))	0.06 ± 0.01	0.12 ± 0.01	0.29 ± 0.05
MRT (min)	146.10 ± 4.67	435.68 ± 39.99	64.98 ± 4.06

a The PK studies and all of the in vivo studies were conducted using male Wistar rats (*n* = 3).

### *In Vivo* Pharmacological Profiling

The main goal of the behavioral studies was to obtain proof-of-concept at the *in vivo* level for the novel ligand activities in a head-to-head comparison to a series of reference drugs commonly prescribed to manage BPSD. Therefore, we selected a group of behavioral tests to evaluate the pharmacological profiles of **11** and **16** against comparator psychotropic drugs (Table 7). Given the multireceptor profile of the selected compounds, characterized by high affinities for 5-HT_2A_, 5-HT_7_, 5-HT_6_, and D_2_ receptors, we expected antidepressant, anxiolytic or antipsychotic activities to be revealed in *in vivo* models. Therefore, key compounds were surveyed in the forced swim test (FST, or Porsolt test), one of the most widely used procedures to assess antidepressant-like activities, as well as the Vogel conflict drinking test, to reflect potential anxiolytic-like activity and MK-801-induced hyperlocomotion, to examine antipsychotic-like activity (Table 7). In light of the fact that social skills and cognitive safety are crucial issues regarding the fragile population of geriatric patients, these results provide an indication that it could be possible to develop specifically tailored multifunctional pharmacophores that could form the basis of more advanced, safe, and selective therapeutics to address the critical unmet medical need of this patient group.

**Table 7:** Characterization of Selected Compounds in Behavioral *In Vivo* Pharmacological Tests in Comparison to Reference Drugs^a^

compd	FST MED [mg/kg]	Vogel test MED [mg/kg]	EPM MED [mg/kg]	MK-801-induced hyperactivity MED [mg/kg]	spontaneous locomotor activity MSD [mg/kg]
**7**	>1	>1		>3	
**11**	0.1	>10		>10	>0.3
**16**	>3	1	1	>10	>3
**Imipramine**	10				10
**S-citalopram**	20				
**Quetiapine**	100	3	1	>100	100
**Risperidone**	3			0.3	1
**Olanzapine**	3		1	3	1
**Diazepam**		5	2.5		3
**Buspirone**		1.25	1.25		
**Aripiprazole**(64)	3	10^64^	>3^64^	>100	10
**Pimavanserin**(68)				0.1^b^	0.1^b^

a Tests carried out on Wistar rats (*n* = 8) after intraperitoneal (i.p.) administration. MED: minimum effective dose; MSD: minimum sedative dose, blank spaces—compounds not tested in the assay.

b Experiments performed in mice.^68^

#### Forced Swim Test (FST)

Among the tested compounds, only **11** elicited pronounced antidepressant-like activity, as determined in the FST, at relatively low doses: 0.1 and 0.3 mg/kg, 100- and 66-fold lower than those observed for the reference drugs imipramine or escitalopram (MED = 10 and 20 mg/kg, respectively). Of note, alterations of mood manifested by dementia subjects, such as depressive symptoms, apathy, and irritability have been attempted to be managed with a “pharmacological cocktail” composed of antidepressants and adjunctive antipsychotics. In fact, it has been widely accepted that the addition of antipsychotic drugs to SSRI treatment produces a synergic effect in the treatment of unresponsive depressive patients.^57−59^ Antipsychotics like olanzapine or clozapine exert 5-HT_6_ and 5-HT_7_ receptor antagonism in the cortex and mesolimbic areas.^60,61^ Moreover, atypical antipsychotics block D_2_ and 5-HT_2A_ receptors, which may promote indirect enhanced dopamine release in medial prefrontal circuits.^62^ These characteristic features are believed to provide synergic antidepressant effects which help to reach therapeutically acceptable responses in depressed patients. Notably, 5-HT_6_, 5-HT_7_, 5-HT_2A_, and D_2_ receptors antagonism remain unreachable for most antidepressants.^63^ Compound **11** shares some similarities with antipsychotics in terms of visible affinity for the 5-HT_6_, 5-HT_7_, 5-HT_2A_, and D_2_ receptors. We decided to compare the performance of **11** in the FST against the selected antipsychotics: risperidone, quetiapine, and olanzapine. We observed that the reference drugs developed antidepressant-like activity at 10-fold to 100-fold higher doses, namely: 3 mg/kg (risperidone, olanzapine, and aripiprazole) and at 100 mg/kg (quetiapine), compared with **11**.^64^ Moreover, while being active in the FST, compound **11** did not influence the spontaneous locomotor activity at active doses (0.1 and 0.3 mg/kg), clearly suggesting that the observed results arise from a specific antidepressant effect of **11**. Furthermore, **11** is characterized by proportionate affinities and functional activities toward all four receptors. The observed results confirm the earlier assumption that the robust antidepressant-like effect of **11** might be attributed to the extensive blockade of 5-HT_6_ and 5-HT_7_ receptors, which is potentiated by synergic efficacy derived from its interaction with 5-HT_2A_ and D_2_ receptors. The functional interaction between 5-HT_6_, 5-HT_7_, 5-HT_2A_, and D_2_ receptors may affect the activity of prefrontal–subcortical circuits, in a similar manner that antipsychotics exert antidepressant responses.^62^ Compounds **7** and **16** did not show statistically significant efficacy in the FST in the range of tested doses (Table 7).

#### Vogel Conflict Drinking Test

Next, we investigated the potential anxiolytic activity of the key compounds in the Vogel conflict drinking test. Among the tested compounds, **16** produced statistically significant anxiolytic effects at a minimum effective dose of 1 mg/kg. In this regard, **16** showed similar efficacy to quetiapine (MED = 3 mg/kg) and the anxiolytic medication buspirone (MED = 1.25 mg/kg), while being more active than the multimodal antipsychotic, aripiprazole (MED = 10 mg/kg) and the gold standard anxiolytic, diazepam (MED = 5 mg/kg). Highly noteworthy, in contrast to diazepam,^65^ and aripiprazole, **16** did not influence spontaneous locomotor activity at a tested dose of 3 mg/kg (Table 7). The anxiolytic-like activity of **16** in the Vogel conflict drinking test seems to be specific, since the compound given at a dose of 1 mg/kg did not affect the pain reaction and unpunished water consumption in water-deprived rats.

The compounds’ efficacy was further explored in the elevated plus maze (EPM) test, in which **16** significantly and dose-dependently enhanced the total open arm time. Together, **16** showed consistent anxiolytic-like efficacy at a low dose (1 mg/kg) in two separate tests of anxiolytic activity in rats. Compound **16** displayed a distinctive high affinity for the 5-HT_2A_ receptor (1-fold p*K*_i_) compared to the other serotonin subtypes 5-HT_6_, 5-HT_7_, as well as D_2_ receptors. The 5-HT_2A_ receptor modulates a diverse array of behavioral responses related to fear and anxiety. Genetic studies with 5-HT_2A_ receptor knock-out mice showed anxiety-like symptoms with the absence of depression-related behaviors.^66^ Further studies suggested that pharmacological modulation of 5-HT_2A_ receptor activity may restore cortical signaling and normalize anxiety-like behavior. We believe that the dominant blockade of the 5-HT_2A_ receptor accounts for the pronounced anxiolytic effect displayed by **16**. These results are in line with other authors’ observations verifying the pronounced blockade of 5-HT_2A_ receptor activity mediates predominantly anxiolytic-like effect.^67^ Compounds **7** and **11** did not show statistically significant efficacy in the Vogel conflict drinking test in the range of tested doses (Table 7).

#### MK-801-Induced Hyperactivity

Key compounds were further probed in the MK-801-induced hyperactivity test. Compounds **7** at 1 mg/kg, **11** at 3 mg/kg, and **16** at 3 mg/kg tended to decrease the total distance animals traveled. However, the effects were not statistically significant.

Despite possessing an affinity for D_2_ and 5-HT_2A_ receptors, the tested compounds did not show a clear antipsychotic-like efficacy in terms of significant suppression of MK-801-induced hyperlocomotion. We compared the functional responses of **7**, **11**, and **16** at D_2_ receptor with structurally related sulfonamide series previously developed by our group.^36^ The lead structure of the latter series was characterized by pronounced antipsychotic-like activity and relatively high antagonistic properties at D_2_ receptors (p*K*_B_ = 9.03) compared to 5-HT_6_ (p*K*_B_ = 7.41), 5-HT_7_ (p*K*_B_ = 8.72), and 5-HT_2A_ (p*K*_B_ = 8.79) receptors. The structural modifications resulting in sulfone and methylpyrrolidine series were primarily introduced to improve metabolic stability and aqueous solubility compared to the original sulfonamides. The change in the in vivo pharmacological profile that was simultaneously encountered may be attributed to the comparatively lower antagonistic properties for D_2_ (p*K*_B_ = 6.73–8.21) and 5-HT_2A_ (p*K*_B_ = 7.79–7.99) receptors versus 5-HT_6_ (p*K*_B_ = 6.74–7.44) and 5-HT_7_ (p*K*_B_ = 6.40–7.42) receptors. These seemingly subtle differences might have possibly influenced the *in vivo* efficacy and resulted in nonsignificant alteration of MK-801-induced hyperlocomotion. Similar features have been observed by other authors who reported multifunctional ligands with surmountable antagonism for D_2_R and the lack of significant antipsychotic-like activity.^68^

The antipsychotic activity of small molecules may also be attributed to interaction with the 5-HT_2A_ receptor. A selective 5-HT_2A_ inverse agonist, pimavanserin exerts an antipsychotic-like effect in rodents and has been recently approved for the treatment of Parkinson’s disease-related psychosis. However, one has to notice that pimavanserin possesses a unique mechanism of action and outstandingly high affinity for 5-HT_2A_ receptor (p*K*_i_ = 10) and the lack of significant affinity for 5-HT_6_ and 5-HT_7_ receptors, which accounts for its pronounced antipsychotic-like effect.^69^ In contrast, compounds **7**, **11**, and **16** exert lower affinity for 5-HT_2A_ receptor compared to pimavanserin and high affinity for 5-HT_6_, 5-HT_7_ receptors, which may explain the diverse pharmacological profile of the novel chemotypes.

#### Social Interaction Test (SIT)

**Figure 7:**
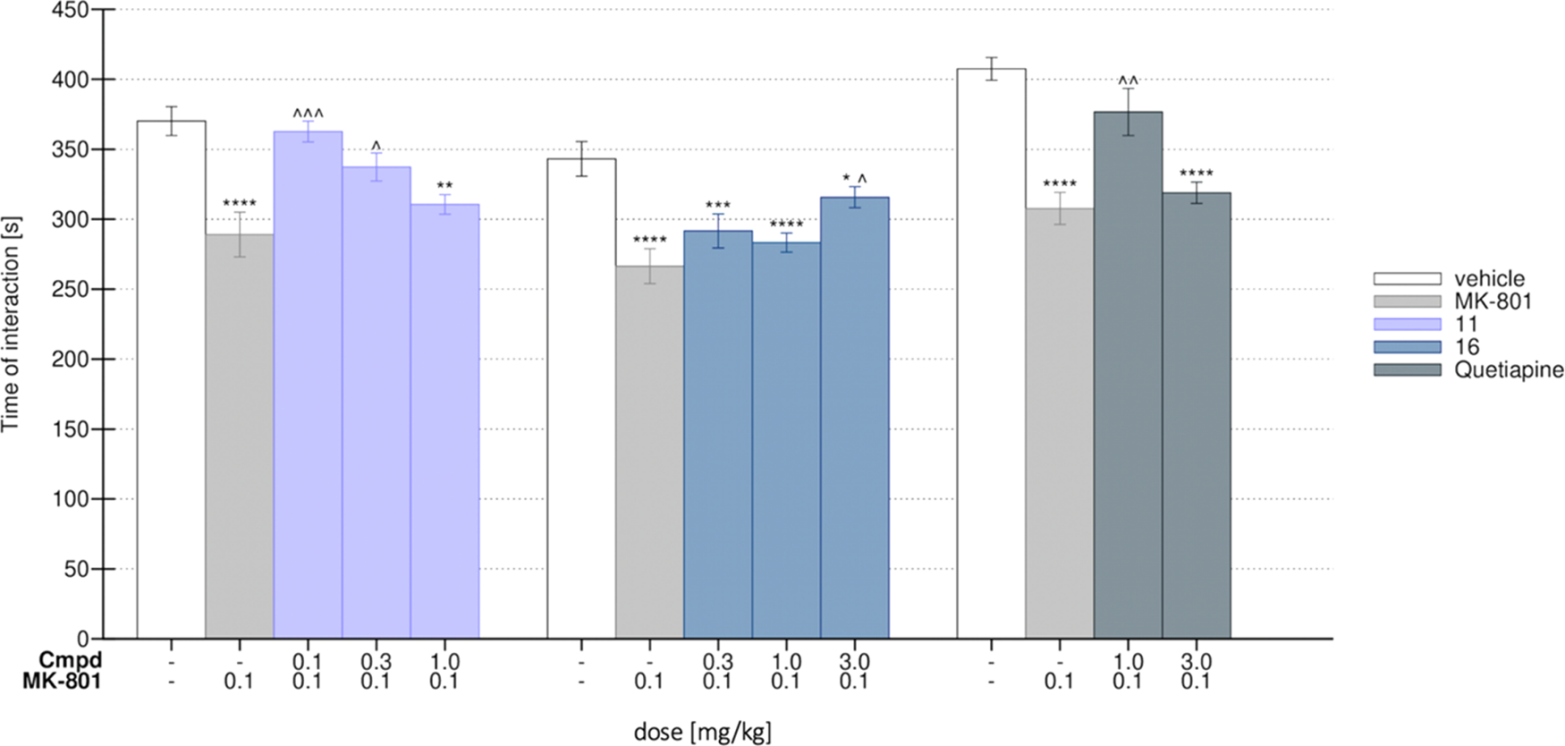
Effects of **11** and **16** on MK-801-induced deficits in the social interaction test (SIT) in Wistar rats. Control—vehicle-treated group (white bar), MK-801 group—after administration of 0.1 mg/kg (light gray bar), tested compound **11** (lavender blue), compound **16** (navy bar), quetiapine (dark gray). *N* = 7–8 pairs of rats per group Statistical analysis: one-way analysis of variance (ANOVA) (Bonferroni’s post hoc); *p < 0.05, **p < 0.01, ***p < 0.001, ****p < 0.0001 vs control (saline, white); ^∧^*p* < 0.05, ^∧∧^*p* < 0.01, ^∧∧∧^*p* < 0.001 vs MK-801-treated group (light gray).

One of the symptoms commonly observed in dementia subjects is social withdrawal that poses a great challenge for caregivers and family members. An animal model of impaired social functioning relies on a single administration of MK-801 to rats, which induces significant deficiencies in their social activities.^70^ We have used MK-801 to induce social withdrawal in rats and next examined the influence of the most promising compounds, **11** and **16**, on social skills. Remarkably, administration of **11** at 0.1 and 0.3 mg/kg significantly reversed the social deficit induced by MK-801. The group treated with compound **11** spent significantly more time exploring an unknown rat, compared to the MK-801-treated group. For comparison, quetiapine, the antipsychotic drug used in the therapy of BPSD, was active in the SIT at a dose of 1.0 mg/kg. Compound **16**, at 3 mg/kg, reversed social withdraw induced by MK-801 (Figure 7). These results suggest that both compounds display a favorable effect on social interactions, which is of particular significance in the view of the disrupted social functioning in BPSD patients.

#### Assessment of Cognitive Safety

**Figure 8:**
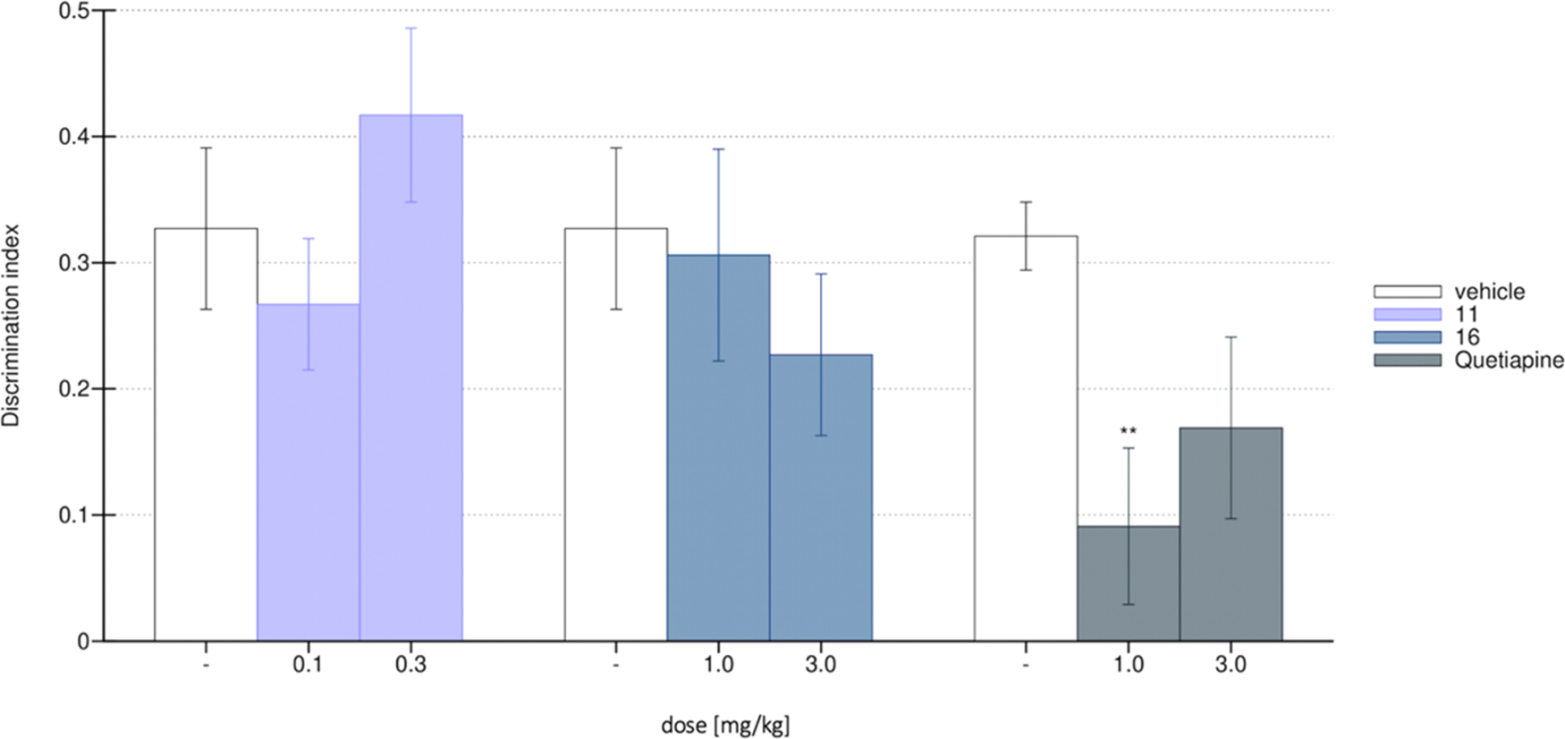
Novel object recognition task (NORT) with MK-801-induced memory deficit. Control group—white bar, compound **11**—lavender, **16**—blue, quetiapine—gray. *N* = 7–8 rats per group. Statistical analysis: one-way ANOVA (Bonferroni’s post hoc); **p < 0.01 vs control (saline, white).

Potential cognitive impairment has been recognized as a critical adverse reaction, which may be triggered by CNS-acting drugs. BPSD patients seem to be particularly susceptible to cognitive slowing induced by psychotropic medications. Therefore, assessing cognitive safety remains a crucial issue for compounds designed for the fragile population of geriatric BPSD patients. To assess the cognitive safety of the lead molecules developed in this study, we used the novel object recognition task (NORT), which is a commonly applied behavioral paradigm to examine cognitive performance.^71,72^ Administration of both compounds, **11** and **16**, at pharmacologically effective doses, namely: **11** at 0.1–0.3 mg/kg and **16** at 1–3 mg/kg, did not affect the NORT performance, suggesting their safety regarding cognitive performance (Figure 8). All of the groups explored the novel object for a similar amount of time. Under these conditions, the reference compound quetiapine, administered at a dose of 1 mg/kg, markedly decreased NORT performance, confirming its harmful effect on cognitive functions.

Altogether, these results suggest that compounds **11** and **16** appear to be useful for addressing depressive-like or anxiety-like symptoms, respectively, at relatively low doses (0.1 and 1 mg/kg, respectively). Both compounds do not impair social functioning. In contrast to the majority of psychotropic medications, both compounds do not influence spontaneous locomotor activity and do not induce cognitive deficits. These findings appear to be remarkably important regarding the targeted population of dementia patients and further validates our pharmacological strategy to treat BPSD.

#### Metabolic and Cardiac Safety

As stated in numerous clinical observations, dementia patients are highly sensitive to drug-induced metabolic syndrome and harmful cardiovascular events, particularly those that result from interaction with undesirable biological targets. The interesting pharmacological profile that emerged for **11** prompted us to choose it as a model anti-BPSD agent to investigate its effect on blood pressure, lipid profile, glucose levels, body fat, and weight as well as its effect on selected liver enzymes in plasma.

**Figure 9:**
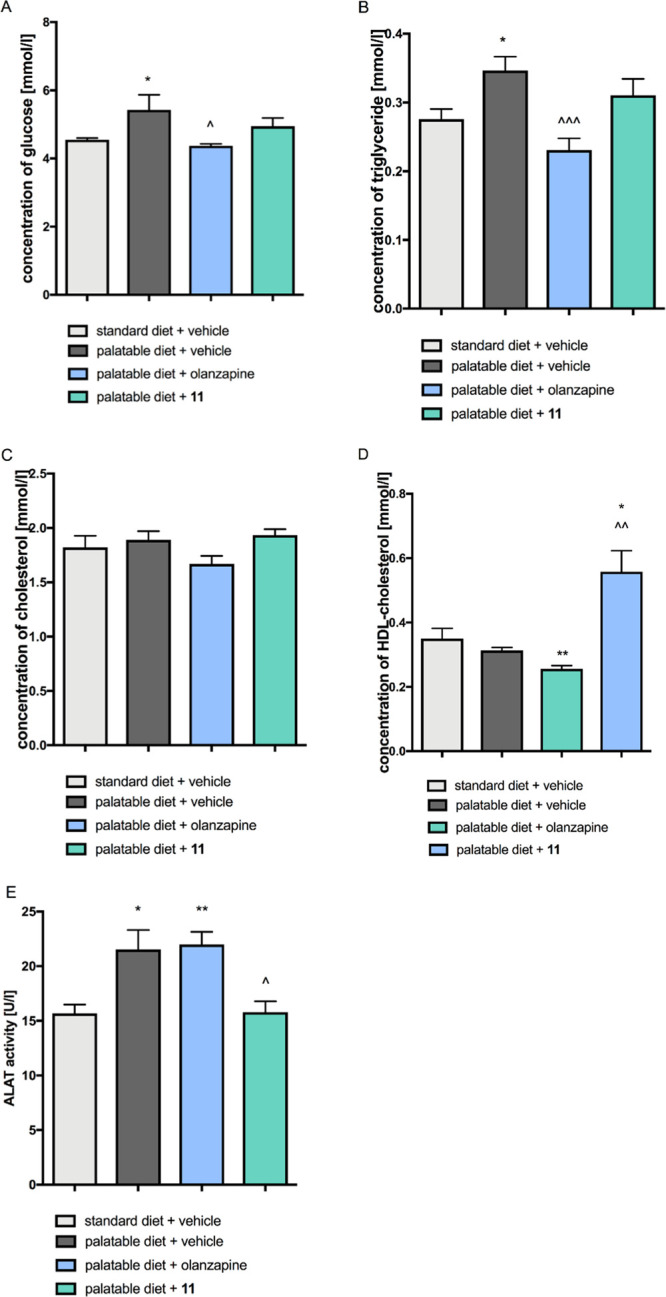
Effect of **11** and olanzapine on plasma biochemical parameters: A. glucose, B. triglyceride, C. total cholesterol, D. HDL-cholesterol, E. alanine aminotransferase. Statistical analysis: one-way ANOVA (Tukey *post hoc*); *p < 0.05, **p < 0.01 vs vehicle + standard diet; ^∧^*p* < 0.05, ^∧∧^*p* < 0.01 vs vehicle + palatable diet; mean ± SEM, *n* = 6.

**Figure 10:**
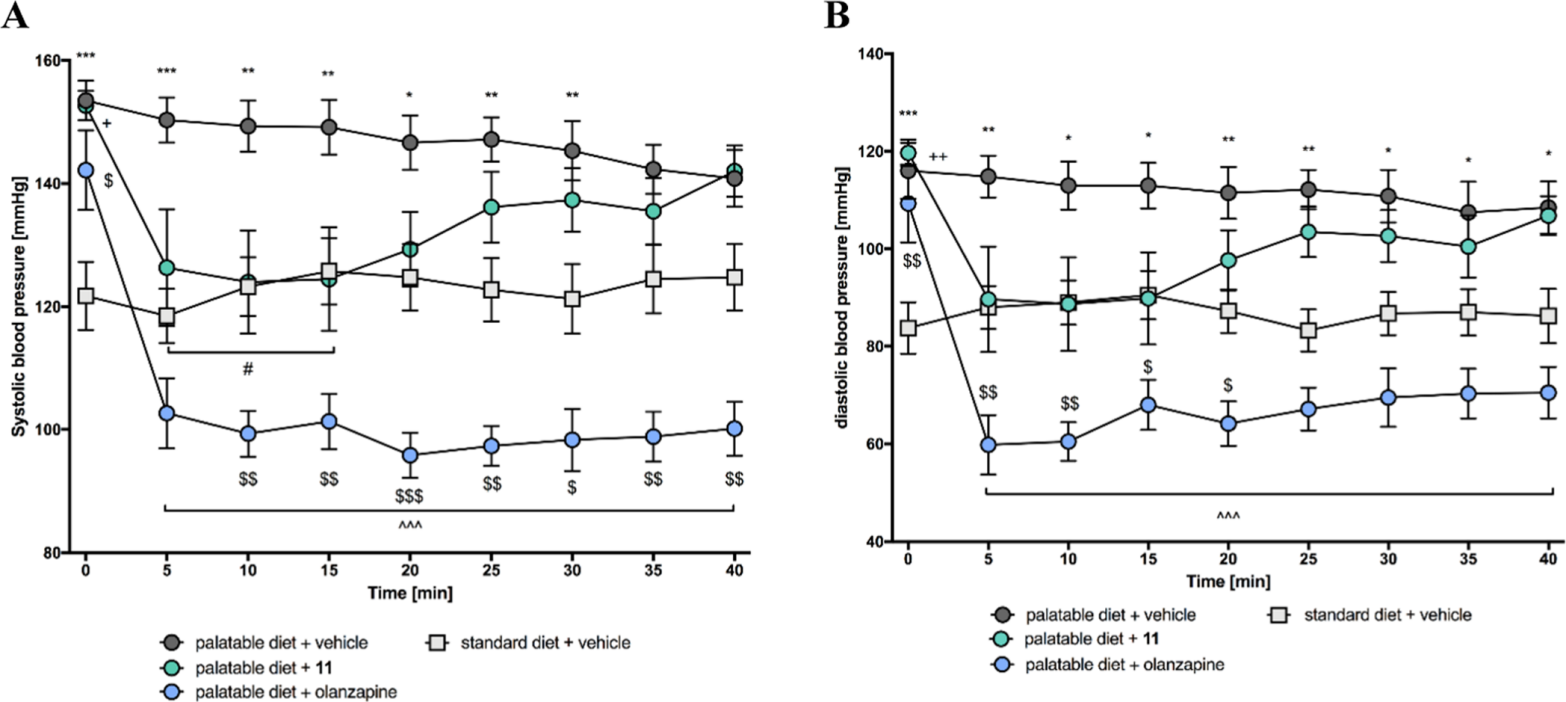
Time course of the effect of **11** or olanzapine on blood pressure: A. systolic, B. diastolic. Statistical analysis: two-way ANOVA with repeated measure (Bonferroni *post hoc*); *p < 0.05, **p < 0.01, ***p < 0.001 vehicle + standard diet vs vehicle + palatable diet; ^∧∧∧^*p* < 0.001 vehicle + palatable diet vs olanzapine + palatable diet; #*p* < 0.05 vehicle + palatable diet vs **11** + palatable diet; +*p* < 0.05 vehicle + standard diet vs **11** + palatable diet; $*p* < 0.05, $$*p* < 0.01, $$$*p* < 0.001 vehicle + standard diet vs olanzapine + palatable diet; mean ± SEM, *n* = 6.

During the 25-day study, the animals were fed according to a “cafeteria diet”, a protocol developed previously in our labs, which was successfully implemented in the evaluation of metabolic safety of novel compounds.^73−76^ The cafeteria diet relies on the animals’ free access and preference for palatable products, which they tend to consume in excess, and this paradigm facilitates the development of the potential metabolic syndrome. We used olanzapine (at a dose of 2 × 2 mg/kg b.w./day, i.p.) as a comparator in this study, due to its well-known ability to induce metabolic syndrome. After the 25-day study, we observed that the animals exposed to a palatable diet and chronic dosing of **11** (2 × 2 mg/kg b.w., i.p.), had significantly lower weight compared to the control group (animals fed with palatable diet and treated with vehicle) (Figure S15 Supporting Information). Notably, compound **11**, in contrast to olanzapine, did not significantly affect plasma glucose, triglyceride, or total cholesterol levels (Figure 9A–C), and favorably increased HDL-cholesterol levels (Figure 9D). Moreover, compound **11** did not affect the activity of alanine aminotransferase in rats that experienced chronic dosing, whereas olanzapine significantly increased its activity at the chronic dosing used (Figure 9E). At the end of the study (25th day), we observed a slight decrease in systemic blood pressure (systolic and diastolic) in the test group; however, the level of blood pressure was adjusted to the level of arterial pressure observed in lean rats, which clearly indicates the significant benefit of compound **11** at the dosage tested (Figure 10A,B). The effect of **11** was beneficially differentiated from olanzapine, which significantly reduced both systolic and diastolic blood pressure to a very large extent, when tested under the same experimental conditions.

In summary, these results indicate that, along with its promising therapeutic-like efficacy, compound **11** does not induce adverse reactions, such as significant metabolic disturbances nor fluctuations of blood pressure, in rats at the dosages tested. These results seem to be particularly important, considering that elderly patients are highly sensitive to drug-induced metabolic disturbances and orthostatic hypotension.

## Conclusions

The hypothesis that BPSD patients might benefit from specifically designed agents that interact with clinically relevant targets led us to construct multifunctional ligands, **2**–**22**, that interact simultaneously with a defined set of molecular targets: 5-HT_2A_, 5-HT_6_, 5-HT_7_, and D_2_ receptors, which are believed to provide therapeutically acceptable profiles. Using the framework combination strategy, we merged a 6-fluoro-3-propylbenzo[*d*]isoxazole core, as a privilege structure for achieving binding to D_2_ and 5-HT_2A_ receptors, with 3-((arylsulfonyl)methyl)pyrrolidine or *N*-(5-methylpyrrolidin-3-yl)benzenesulfonamide, an essential scaffold for providing interaction with 5-HT_6_ and 5-HT_7_ receptors. These hybrid chemophores enabled us to design a series of multifunctional ligands that exert pronounced interactions in the nanomolar range with essentially matched biological targets and also ensured adequate functional responses. The novel chemotypes were surveyed within the cascade of *in vitro* studies, which led us to select the most promising molecules characterized by a low affinity for off-targets: α_1_, H_1_, and M_1_ receptors and hERG channels, as well as showing favorable aqueous solubility. Further, thorough profiling revealed that novel chemotypes are characterized by appreciable metabolic stability, compared to parent sulfonamide counterparts previously reported on.

The present study also led to the conclusion that arylsulfone-based chemotypes, such as compound **11**, display pronounced antidepressant-like activity (MED = 0.1 mg/kg), outperforming marketed antidepressant medications in this regard. This feature is presumably attributed to the high affinity of **11** for 5-HT_6_, 5-HT_7_, and 5-HT_2A_ receptors and synergic activity for these serotonin subtypes. Compound **16**, featuring arylsulfonamide/methylpyrrolidine scaffolds, elicited robust anxiolytic activity, superior to reference anxiolytic drugs. In this regard, novel chemotypes differ substantially from the previously reported sulfonamide derivatives that exerted significant antipsychotic-like activity.^36^ The chemical modifications were introduced to solve two important issues—increase metabolic stability and obtain well-soluble druglike compounds of a given receptor profile. Therefore, the current chemical series constitutes a conscious extension of the previously reported one, which, in addition to achieving the above goals, resulted in a favorable and nonobvious pharmacological activity. In the previous series, the leading functional activity was D_2_R antagonism—it dominated 5-HT_6_ and 5-HT_7_ receptors function, and was even higher than the antagonism of the 5-HT_2A_R. The incremental structural alterations retained the general receptor profile, but introduced relative weakening of affinity and antagonist activity for D_2_R, and relatively stronger interactions with 5-HT_6_ and 5-HT_7_ receptors, making the 5-HT_2A_R antagonism the major mechanism of action. The receptor profile appears to be more balanced in the current series, even though the absolute values of the 5-HT_2A_R functional activity are lower. The above-mentioned in vitro activity differences caused an interesting change in the results of behavioral tests. The compounds lost antipsychotic-like activity (evidently mediated by antagonism of the D_2_ and 5-HT_2A_ receptors) and expressed pure antidepressant- and anxiolytic-like activity.

Alongside their therapeutic-like activities, both compounds did not induce severe side reactions, such as impoverished cognitive functioning, characteristic of most psychotropic medications. The most promising compound, **11**, did not impair glucose homeostasis and did not adversely affect weight and blood pressure. Given that dementia patients are highly vulnerable to severe drug-induced adverse reactions, the pharmacological approach encompassing the two key molecules described in this study, namely, **11** and **16**, may provide a competitive and promising alternative to psychotropic medications used currently in BPSD patients.

## Experimental Section

### Molecular Modeling

Molecular property analysis and prediction of interactions in the binding sites of the target ligands aided in the design of the two series of molecules described in this study. Instant JChem was used for structure database management, search and property prediction, Instant JChem 20.19.0, 2020, ChemAxon (http://www.chemaxon.com). The biological chemistry of the molecules was evaluated in terms of Lipinski’s rule of five criteria: Log *P* o/w (octanol/water partition coefficient) < 5, molecular weight (MW) < 500, H-bond acceptors (HBA) < 10, H-bond donors (HBD) < 5, and the Veber rule criteria: rotatable bonds, preferably RB < 10 and topological polar surface area (TPSA) < 140 A^2^. Metabolic pathway and clearance prediction was conducted using the ADMET Predictor (Simulations Plus, Inc.). The designed structures were examined using the SwissADME tool (http://www.swissadme.ch) for known classes of reactive assay interference compounds (PAINS), and toxicophore structures (Brenk alert).

Docking studies were carried out using the previously developed homology models of the D_2_, 5-HT_2A_, 5-HT_6_, and 5-HT_7_ receptors. The procedure for obtaining ligand-optimized models of high predictive value, utilizing Induced-fit docking (IFD), was characterized in detail previously.^36,77,78^ The dopamine D_2_ receptor homology model was based on the 3PBL structure, the serotonin 5-HT_2A_ receptor was built on 4IB4, the 5-HT_6_ receptor on 4IAR, and the 5-HT_7_ receptor—on the 2RH1 crystal structure.

Ligand structures were optimized using the LigPrep tool. The Glide SP flexible docking procedure was carried out using OPLS3 force field. H-bond constraint, as well as centroid of a grid box for docking to the receptor models were located on Asp3.32. The selection of the presented complexes was based on scoring function and interaction analysis. Molecular modeling studies were supported by the Small-Molecule Drug Discovery Suite (Schrödinger, Inc.).

### General Chemistry Information

Unless otherwise indicated, all of the starting materials were purchased from commercial suppliers and were used without further purification. Analytical thin-layer chromatography (TLC) was performed on Merck Kieselgel 60 F_254_ (0.25 mm) precoated aluminum sheets (Merck, Darmstadt, Germany). Visualization was performed with a 254 nm UV lamp. Column chromatography was performed using silica gel (particle size 0.063–0.200 mm; 70–230 Mesh ATM) purchased from Merck. The ultraperformance liquid chromatography-mass spectrometry (UPLC-MS) or UPLC-MS/MS analyzes were run on an UPLC-MS/MS system comprising a Waters ACQUITY UPLC (Waters Corporation, Milford, MA) coupled with a Waters TQD mass spectrometer (electrospray ionization mode ESI with tandem quadrupole). Chromatographic separations were carried out using an ACQUITY UPLC BEH (bridged ethyl hybrid) C_18_ column: 2.1 mm × 100 mm and 1.7 μm particle size. The column was maintained at 40 °C and eluted under gradient conditions using 95–0% of eluent A over 10 min, at a flow rate of 0.3 mL/min. Eluent A: 0.1% solution of formic acid in water (v/v); eluent B: 0.1% solution of formic acid in acetonitrile (v/v). A total of 10 μL of each sample were injected, and chromatograms were recorded using a Waters eλ PDA detector. The spectra were analyzed in the range of 200–700 nm with 1.2 nm resolution and at a sampling rate of 20 points/s. MS detection settings of the Waters TQD mass spectrometer were as follows: source temperature 150 °C, desolvation temperature 350 °C, desolvation gas flow rate 600 l/h, cone gas flow 100 l/h, a capillary potential 3.00 kV, and cone potential 20 V. Nitrogen was used for both nebulizing and drying. The data were obtained in a scan mode ranging from 50 to 1000 *m*/*z* at 0.5 s intervals; 8 scans were summed up to obtain the final spectrum. Collision activated dissociation (CAD) analyzes were carried out using an energy of 20 eV, and all of the fragmentations were observed in the source. Consequently, the ion spectra were obtained in the range from 50 to 500 *m*/*z*. MassLynx V 4.1 software (Waters) was used for data acquisition. Standard solutions (1 mg/mL) of each compound were prepared in a mixture comprising analytical grade acetonitrile/water (1/1, v/v). The UPLC/MS purity of all of the test compounds and key intermediates was determined to be >95%.

^1^H NMR, ^13^C NMR, and ^19^F NMR spectra were obtained in a Varian Mercury spectrometer (Varian, Inc., Palo Alto, CA), in CDCl_3_, CD_3_OD, or DMSO-*d*_6_, operating at 300 MHz (^1^H NMR), 75 MHz (^13^C NMR), and 282 MHz (^19^F NMR). Chemical shifts are reported in terms of δ values (ppm) relative to TMS δ = 0 (^1^H) as an internal standard. The *J* values are expressed in Hertz (Hz). Signal multiplicities are represented by the following abbreviations: s (singlet), br s (broad singlet), d (doublet), dd (doublet of doublets), ddd (doublet of doublets of doublets), dt (doublet of triplets), t (triplet), td (triplet of doublets), tdd (triplet of doublet of doublets), q (quartet), dq (doublet of quartets), qd (quartet of doublets), quin (quintet), m (multiplet) and related.

Enantiomeric purity was determined using a chiral high-performance liquid chromatography (HPLC) technique on a Shimadzu Prominence LC-2030C SD Plus system (Shimadzu Corporation, Kyoto, Japan) equipped with an Amylose-C (250 mm × 4.6 mm) chiral column. The analysis was performed under the following conditions; column temperature: 45 °C, mixture of eluents: hexane/i-PrOH/TFA = 93/6.9/0.1 (v/v), flow rate: 1.4 mL/min, injection volume: 10 μL, analysis time: 70 min. (isocratic), detection at the wavelength λ = 200–800 nm. Enantiomeric purity is expressed in %.Detailed procedures for preparation of the synthesis intermediates **I–VI** are provided in the Supporting Information.

#### General Procedure for the Synthesis of Final Compounds **2–22**

A mixture of appropriate amine **IIIa–k** (1 equiv), anhydrous potassium carbonate (3 equiv), 3-(3-chloropropyl)-6-fluorobenzo[*d*]isoxazole **IV** (1 equiv), and a catalytic amount of potassium iodide in acetonitrile (10 mL) was stirred at 60 °C for 24 h. After that time, the anhydrous potassium carbonate was filtered off and the solvent was evaporated under reduced pressure. The reaction mixture was purified by flash column chromatography using n-hexane/dichloromethane/methanol (60:35:5) (v/v/v) as eluent.

##### 6-Fluoro-3-(3-(3-(((naphthalene-2-yl-phenyl)sulfonyl)methyl)pyrrolidin-1-yl)propyl)benzo[*d*]isoxazole (**2**)

The title compound was prepared starting from 3-{[(naphthalene-2-yl-phenyl)sulfonyl]methyl}pyrrolidine hydrochloride (0.3 mmol, 1 equiv, 0.094 g), 3-(3-chloropropyl)-6-fluorobenzo[*d*]isoxazole (0.3 mmol, 1 equiv, 0.064 g), potassium carbonate (0.9 mmol, 3 equiv, 0.124 g), and a catalytic amount of potassium iodide. Yield: 32%, brown oil. ^1^H NMR (300 MHz, CDCl_3_, δ): 8.48 (s, 1H), 8.04–7.96 (m, 2H), 7.93 (d, *J* = 7.6 Hz, 1H), 7.86 (dd, *J* = 1.8, 8.8 Hz, 1H), 7.72–7.55 (m, 3H), 7.21 (dd, *J* = 2.1, 8.5 Hz, 1H), 7.05 (dt, *J* = 2.3, 8.8 Hz, 1H), 3.33–3.17 (m, 2H), 2.98 (t, *J* = 7.3 Hz, 2H), 2.85–2.76 (m, 1H), 2.73–2.41 (m, 6H), 2.15–1.91 (m, 3H), 1.54 (qd, *J* = 6.6, 14.7 Hz, 1H). ^13^C NMR (75 MHz, CDCl_3_, δ): 163.6 (d, *J* = 13.8 Hz), 164.2 (d, *J* = 252.0 Hz), 158.1, 136.4, 135.3, 132.2, 129.8, 129.7, 129.4, 129.3, 128.0, 127.8, 122.6, 122.2 (d, *J* = 11.5 Hz), 118.2, 112.4 (d, *J* = 25.3 Hz), 97.3 (d, *J* = 27.7 Hz), 61.3, 59.2, 55.0, 53.1, 31.9, 30.5, 26.4, 22.9. LC-MS (ESI) calcd for C_25_H_25_FN_2_O_3_S 452.54 [M + H^+^], found 453 [M + H^+^].

##### 6-Fluoro-3-(3-(3-(((3,4-dimethylphenyl)sulfonyl)methyl)-1-pyrrolidinyl)propyl)benzo[*d*]isoxazole (**3**)

The title compound was prepared starting from 3-{[(3,4-dimethylphenyl)sulfonyl]methyl}pyrrolidine hydrochloride (0.3 mmol, 1 equiv, 0.087 g), 3-(3-chloropropyl)-6-fluorobenzo[*d*]isoxazole (0.3 mmol, 1 equiv, 0.064 g), potassium carbonate (0.9 mmol, 3 equiv, 0.124 g) and catalytic amounts of potassium iodide. Yield: 27%, cream oil.^1^H NMR (300 MHz, CDCl_3_, δ): 7.69–7.55 (m, 3H), 7.33–7.27 (m, 1H), 7.22 (dd, *J* = 2.1, 8.5 Hz, 1H), 7.10–7.01 (m, 1H), 3.23–3.09 (m, 2H), 3.01 (t, *J* = 7.3 Hz, 2H), 2.88–2.79 (m, 1H), 2.73–2.48 (m, 5H), 2.33 (s, 6H), 2.27–2.18 (m, 1H), 2.10–1.99 (m, 3H), 1.65–1.54 (m, 1H). ^13^C NMR (75 MHz, CDCl_3_, δ): 163.8 (d, *J* = 18.4 Hz), 164.2 (d, *J* = 253.0 Hz), 158.0, 143.5, 138.2, 136.6, 130.4, 128.7, 125.5, 122.2 (d, *J* = 11.5 Hz), 118.2, 112.5 (d, *J* = 25.3 Hz), 97.3 (d, *J* = 27.7 Hz), 61.1, 59.1, 55.0, 53.2, 31.8, 30.5, 26.2, 22.9, 20.0, 19.8. LC-MS (ESI) calcd for C_23_H_27_FN_2_O_3_S 430.53 [M + H^+^], found 431 [M + H^+^].

##### 6-Fluoro-3-(3-(3-(((3,4-difluorophenyl)sulfonyl)methyl)pyrrolidin-1-yl)propyl)benzo[*d*]isoxazole (**4**)

The title compound was prepared starting from 3-{[(3,4-difluorophenyl)sulfonyl]methyl} pyrrolidine hydrochloride (0.3 mmol, 1 equiv, 0.089 g), 3-(3-chloropropyl)-6-fluorobenzo[*d*]isoxazole (0.3 mmol, 1 equiv, 0.064 g), potassium carbonate (0.09 mmol, 3 equiv, 0.124 g), and a catalytic amount of potassium iodide. Yield: 25%, yellowish oil. ^1^H NMR (300 MHz, CDCl_3_, δ): 7.80–7.65 (m, 2H), 7.59 (dd, *J* = 5.0, 8.5 Hz, 1H), 7.43–7.31 (m, 1H), 7.22 (dd, *J* = 2.1, 8.5 Hz, 1H), 7.10–7.00 (m, 1H), 3.26–3.09 (m, 2H), 2.99 (t, *J* = 7.3 Hz, 2H), 2.80–2.70 (m, 1H), 2.66–2.36 (m, 6H), 2.15–1.92 (m, 3H), 1.52 (tdd, *J* = 6.4, 8.2, 12.9 Hz, 1H). ^13^C NMR (75 MHz, CDCl_3_, δ): 163.6 (d, *J* = 13.8 Hz), 164.2 (d, *J* = 251.0 Hz), 158.2, 150.5 (d, *J* = 268.3 Hz), 150.3 (d, *J* = 270.0 Hz), 136.7–136.1 (m), 125.4–125.2 (m), 122.2 (d, *J* = 11.5 Hz), 118.6 (d, *J* = 18.4 Hz), 118.3, 118.0 (d, *J* = 20.0 Hz), 112.4 (d, *J* = 25.3 Hz), 97.3 (d, *J* = 26.5 Hz), 61.4, 59.2, 54.9, 53.0, 31.8, 30.5, 26.5, 22.9. LC-MS (ESI) calcd for C_21_H_21_F_3_N_2_O_3_S 438.46 [M + H^+^], found 439 [M + H^+^].

##### 6-Fluoro-3-(3-(3-(((3,4-dichlorophenyl)sulfonyl)methyl)-1-pyrrolidinyl)propyl)benzo[*d*]isoxazole (**5**)

The title compound was prepared starting from 3-{[(3,4-dichlorophenyl)sulfonyl]methyl}pyrrolidine hydrochloride (0.3 mmol, 1 equiv, 0.099 g), 3-(3-chloropropyl)-6-fluorobenzo[*d*]isoxazole (0.3 mmol, 1 equiv, 0.064 g), potassium carbonate (0.9 mmol, 3 equiv, 0.124 g), and a catalytic amount of potassium iodide. Yield: 57%, yellowish oil. ^1^H NMR (300 MHz, CDCl_3_, δ): 7.99 (d, *J* = 1.8 Hz, 1H), 7.77–7.69 (m, 1H), 7.67–7.63 (m, 1H), 7.60 (dd, *J* = 5.0, 8.5 Hz, 1H), 7.22 (dd, *J* = 2.1, 8.5 Hz, 1H), 7.06 (dt, *J* = 1.8, 8.8 Hz, 1H), 3.26–3.09 (m, 2H), 3.00 (t, *J* = 7.6 Hz, 2H), 2.82–2.72 (m, 1H), 2.68–2.39 (m, 6H), 2.18–1.93 (m, 3H), 1.53 (tdd, *J* = 6.5, 8.4, 13.1 Hz, 1H). ^13^C NMR (75 MHz, CDCl_3_, δ): 163.6 (d, *J* = 13.8 Hz), 164.2 (d, *J* = 251.0 Hz), 158.1, 139.3, 138.9, 134.2, 131.6, 130.0, 127.0, 122.2 (d, *J* = 11.5 Hz), 118.3, 112.5 (d, *J* = 25.3 Hz), 97.3 (d, *J* = 27.6 Hz), 61.4, 59.2, 54.9, 53.1, 31.7, 30.5, 26.5, 22.9. LC-MS (ESI) calcd for C_21_H_21_Cl_2_FN_2_O_3_S 471.37 [M + H^+^], found 471 [M + H^+^].

##### 6-Fluoro-3-(3-(3-(((3-chloro-4-fluorophenyl)sulfonyl)methyl)pyrrolidin-1-yl)propyl)benzo[*d*]isoxazole (**6**)

The title compound was prepared starting from 3-{[(3-chloro-4-fluorophenyl)sulfonyl]methyl}pyrrolidine hydrochloride (0.3 mmol, 1 equiv, 0.094 g), 3-(3-chloropropyl)-6-fluorobenzo[*d*]isoxazole (0.3 mmol, 1 equiv, 0.064 g), potassium carbonate (0.9 mmol, 3 equiv, 0.124 mg), and a catalytic amount of potassium iodide. Yield: 8%, yellowish oil. ^1^H NMR (300 MHz, CDCl_3_, δ): 7.98 (dd, *J* = 2.1, 6.7 Hz, 1H), 7.81 (ddd, *J* = 2.3, 4.4, 8.5 Hz, 1H), 7.60 (dd, *J* = 5.0, 8.5 Hz, 1H), 7.33 (t, *J* = 8.5 Hz, 1H), 7.22 (dd, *J* = 2.1, 8.5 Hz, 1H), 7.06 (dt, *J* = 1.8, 8.8 Hz, 1H), 3.27–3.09 (m, 2H), 3.00 (t, *J* = 7.6 Hz, 2H), 2.82–2.71 (m, 1H), 2.69–2.37 (m, 6H), 2.16–1.94 (m, 3H), 1.53 (tdd, *J* = 6.4, 8.2, 12.9 Hz, 1H). ^13^C NMR (75 MHz, CDCl_3_, δ): 163.6 (d, *J* = 13.8 Hz), 164.2 (d, *J* = 251.0 Hz), 161.4 (d, *J* = 258.3 Hz), 158.1, 136.7 (d, *J* = 3.5 Hz), 131.1, 128.6 (d, *J* = 8.1 Hz), 123.0, 122.2 (d, *J* = 11.4 Hz), 118.2, 117.7 (d, *J* = 23.0 Hz), 112.5 (d, *J* = 25.3 Hz), 97.3 (d, *J* = 27.7 Hz), 61.5, 59.2, 54.9, 53.1, 31.7, 30.5, 26.5, 22.9. LC-MS (ESI) calcd for C_21_H_21_ClF_2_N_2_O_3_S 454.91 [M + H^+^], found 455 [M + H^+^].

##### 6-Fluoro-3-(3-(3-(((4-(trifluoromethyl)phenyl)sulfonyl)methyl)pyrrolidin-1-yl)propyl)benzo[*d*]isoxazole (**7**)

The title compound was prepared starting from 3-{[(4-(trifluoromethyl)phenyl) sulfonyl] methyl}pyrrolidine hydrochloride (0.3 mmol, 1 equiv, 0.099 g), 3-(3-chloropropyl)-6-fluorobenzo[*d*]isoxazole (0.3 mmol, 1 equiv, 0.064 g), potassium carbonate (0.9 mmol, 3 equiv, 0.124 g), and a catalytic amount of potassium iodide. Yield: 67%, yellowish oil. ^1^H NMR (300 MHz, CDCl_3_, δ): 8.05 (d, *J* = 8.2 Hz, 2H), 7.84 (d, *J* = 8.2 Hz, 2H), 7.59 (dd, *J* = 5.0, 8.5 Hz, 1H), 7.22 (dd, *J* = 2.1, 8.5 Hz, 1H), 7.11–7.00 (m, 1H), 3.28–3.10 (m, 2H), 3.00 (t, *J* = 7.3 Hz, 2H), 2.82–2.71 (m, 1H), 2.69–2.38 (m, 6H), 2.17–1.93 (m, 3H), 1.53 (tdd, *J* = 6.7, 8.3, 13.0 Hz, 1H). ^13^C NMR (75 MHz, CDCl_3_, δ): 163.6 (d, *J* = 13.8 Hz), 164.2 (d, *J* = 249.9 Hz), 158.1, 143.1, 135.5 (q, *J* = 33.4 Hz), 128.6 (2C), 126.5 (q, *J* = 3.5 Hz, 2C), 122.2 (d, *J* = 10.4 Hz), 118.2, 123.1 (q, *J* = 275.0 Hz), 112.4 (d, *J* = 25.3 Hz), 97.3 (d, *J* = 27.0 Hz), 61.2, 59.2, 54.9, 53.0, 31.7, 30.5, 26.5, 22.9. LC-MS (ESI) calcd for C_22_H_22_F_4_N_2_O_3_S 470.48 [M + H^+^], found 471 [M + H^+^].

##### 6-Fluoro-3-(3-(3-(((3-(trifluoromethyl)phenyl)sulfonyl)methyl)pyrrolidin-1-yl)propyl)benzo[*d*]isoxazole (**8**)

The title compound was prepared starting from 3-{[(3-(trifluoromethyl)phenyl) sulfonyl] methyl}pyrrolidine hydrochloride (0.3 mmol, 1 equiv, 0.099 g), 3-(3-chloropropyl)-6-fluorobenzo[*d*]isoxazole (0.3 mmol, 1 equiv, 0.064 g), potassium carbonate (0.9 mmol, 3 equiv, 0.124 g), and a catalytic amount of potassium iodide. Yield: 63%, yellowish oil. ^1^H NMR (300 MHz, CDCl_3_, δ): 8.18 (s, 1H), 8.11 (d, J = 7.6 Hz, 1H), 7.92 (br d, J = 8.2 Hz, 1H), 7.79–7.67 (m, 1H), 7.59 (dd, J = 4.7, 8.8 Hz, 1H), 7.22 (dd, J = 2.1, 8.5 Hz, 1H), 7.05 (dt, J = 1.8, 8.8 Hz, 1H), 3.29–3.10 (m, 2H), 3.00 (t, J = 7.6 Hz, 2H), 2.822.73 (m, 1H), 2.71–2.38 (m, 6H), 2.17–1.94 (m, 3H), 1.54 (tdd, J = 6.4, 8.2, 12.9 Hz, 1H). ^13^C NMR (75 MHz, CDCl_3_, δ): 163.6 (d, J = 14.0 Hz), 164.2 (d, J = 249.9 Hz), 158.1, 140.9, 132.1 (q, J = 33.4 Hz), 131.3, 130.5 (q, J = 3.5 Hz), 130.2, 125.1 (q, J = 3.5 Hz), 122.2 (d, J = 11.5 Hz), 118.2, 123.0 (q, J = 273.0 Hz), 112.4 (d, J = 25.3 Hz), 97.3 (d, J = 27.7 Hz, 1C), 61.3, 59.2, 54.9, 53.1, 31.6, 30.5, 26.5, 22.9. LC-MS (ESI) calcd for C_22_H_22_F_4_N_2_O_3_S 470.48 [M + H^+^], found 471 [M + H^+^].

##### 6-Fluoro-3-(3-(3-(((4-fluorophenyl)sulfonyl)methyl)-1-pyrrolidinyl)propyl)benzo[*d*]isoxazole (**9**)

The title compound was prepared starting from the 3-{[(4-fluorophenyl)sulfonyl]methyl}pyrrolidine hydrochloride (0.3 mmol, 1 equiv, 0.084 g), 3-(3-chloropropyl)-6-fluorobenzo[*d*]isoxazole (0.3 mmol, 1 equiv, 0.064 g), potassium carbonate (0.9 mmol, 3 equiv, 0.124 g), and a catalytic amount of potassium iodide. Yield: 38%, yellowish oil. ^1^H NMR (300 MHz, CDCl_3_, δ): 7.92 (dd, *J* = 5.0, 9.1 Hz, 2H), 7.59 (dd, *J* = 5.3, 8.8 Hz, 1H), 7.29–7.19 (m, 3H), 7.10–7.00 (m, 1H), 3.24–3.09 (m, 2H), 2.99 (t, *J* = 7.3 Hz, 2H), 2.80–2.71 (m, 1H), 2.67–2.36 (m, 6H), 2.14–1.94 (m, 3H), 1.58–1.44 (m, 1H).^13^C NMR (75 MHz, CDCl_3_, δ): 166.3 (d, *J* = 13.8 Hz), 165.8 (d, *J* = 256.0 Hz), 164.7 (d, *J* = 180.8 Hz), 158.2, 135.6 (d, *J* = 3.5 Hz), 130.9 (d, *J* = 10.4 Hz, 2C), 122.2 (d, *J* = 10.4 Hz), 118.3, 116.7 (d, *J* = 21.9 Hz, 2C), 112.4 (d, *J* = 26.5 Hz), 97.3 (d, *J* = 26.5 Hz), 61.5, 59.3, 55.0 53.1, 31.8, 30.5, 26.6, 22.9. LC-MS (ESI) calcd for C_21_H_22_F_2_N_2_O_3_S 420.47 [M + H^+^], found 421 [M + H^+^].

##### 6-Fluoro-3-(3-(3-(((3-fluorophenyl)sulfonyl)methyl)-1-pyrrolidinyl)propyl)benzo[*d*]isoxazole (**10**)

The title compound was prepared starting from the 3-{[(3-fluorophenyl)sulfonyl]methyl} pyrrolidine hydrochloride (0.3 mmol, 1 equiv, 0.084 g), 3-(3-chloropropyl)-6-fluorobenzo[*d*]isoxazole (0.3 mmol, 1 equiv, 0.064 g), potassium carbonate (0.9 mmol, 3 equiv, 0.124 g), and a catalytic amount of potassium iodide. Yield: 82%, yellowish oil. ^1^H NMR (300 MHz, CDCl_3_, δ): 7.74–7.66 (m, 1H), 7.63–7.50 (m, 3H), 7.40–7.30 (m, 1H), 7.22 (dd, *J* = 2.1, 8.5 Hz, 1H), 7.05 (dt, *J* = 2.1, 8.9 Hz, 1H), 3.26–3.10 (m, 2H), 2.99 (t, *J* = 7.6 Hz, 2H), 2.76 (dd, *J* = 7.0, 9.4 Hz, 1H), 2.66–2.37 (m, 6H), 2.16–2.02 (m, 2H), 1.98–1.92 (m, 1H), 1.60–1.46 (m, 1H).^19^F NMR (282 MHz, CDCl_3_, δ): −108.85 (s, 1F), -109.57 (s, 1F). ^13^C NMR (75 MHz, CDCl_3_, δ): 172.8, 163.6 (d, *J* = 13.8 Hz), 164.2 (d, *J* = 251.1 Hz), 162.5 (d, *J* = 252.0 Hz), 158.1, 141.6 (d, *J* = 5.8 Hz), 131.3 (d, *J* = 8.1 Hz), 123.8 (d, *J* = 3.5 Hz), 122.2 (d, *J* = 11.5 Hz), 121.1 (d, *J* = 21.0 Hz), 115.4 (d, *J* = 24.2 Hz), 112.5 (d, *J* = 25.3 Hz), 97.3 (d, *J* = 27.6 Hz), 61.2, 59.2, 55.0, 53.0, 31.7, 30.5, 26.4, 22.9. LC-MS (ESI) calcd for C_21_H_22_F_2_N_2_O_3_S 420.47 [M + H^+^], found 421 [M + H^+^].

##### 6-Fluoro-3-(3-(3-(((3-chlorophenyl)sulfonyl)methyl)-1-pyrrolidinyl)propyl)benzo[*d*]isoxazole (**11**)

The title compound was prepared starting from the 3-{[(3-chlorophenyl)sulfonyl]methyl} pyrrolidine hydrochloride (0.3 mmol, 1 equiv, 0.089 g), 3-(3-chloropropyl)-6-fluorobenzo[*d*]isoxazole (0.3 mmol, 1 equiv, 0.064 g), potassium carbonate (0.9 mmol, 3 equiv, 0.124 g), and a catalytic amount of potassium iodide. Yield: 59%, yellowish oil. ^1^H NMR (300 MHz, CDCl_3_, δ): 7.89 (t, *J* = 1.8 Hz, 1H), 7.82–7.74 (m, 1H), 7.67–7.56 (m, 2H), 7.55–7.46 (m, 1H), 7.22 (dd, *J* = 2.1, 8.5 Hz, 1H), 7.06 (dt, *J* = 1.8, 8.8 Hz, 1H), 3.26–3.09 (m, 2H), 3.00 (t, *J* = 7.6 Hz, 2H), 2.77 (dd, *J* = 7.0, 9.4 Hz, 1H), 2.70–2.35 (m, 6H), 2.15–1.95 (m, 3H), 1.61–1.43 (m, 1H).^13^C NMR (75 MHz, CDCl_3_, δ): 163.6 (d, *J* = 13.8 Hz), 164.2 (d, *J* = 250.6 Hz), 158.2, 141.3, 135.6, 133.9, 130.7, 128.1, 126.1, 122.2 (d, *J* = 11.4 Hz), 118.2, 112.4 (d, *J* = 25.3 Hz), 97.3 (d, *J* = 27.6 Hz), 61.3, 59.3, 55.0, 53.1, 31.7, 30.5, 26.5, 22.9. LC-MS (ESI) calcd for C_21_H_22_FClN_2_O_3_S 436.93 [M + H^+^], found 437 [M + H^+^].

##### 6-Fluoro-3-(3-(3-(((3-methylphenyl)sulfonyl)methyl)-1-pyrrolidinyl)propyl)benzo[*d*]isoxazole (**12**)

The title compound was prepared starting from the 3-{[(3-methylphenyl)sulfonyl]methyl}pyrrolidine hydrochloride (0.3 mmol, 1 equiv, 0.083 g), 3-(3-chloropropyl)-6-fluorobenzo[*d*]isoxazole (0.3 mmol, 1 equiv, 0.064 g), potassium carbonate (0.9 mmol, 1 equiv, 0.124 g), and a catalytic amount of potassium iodide. Yield: 45%, cream oil. ^1^H NMR (300 MHz, CDCl_3_, δ): 7.73–7.65 (m, 2H), 7.60 (dd, *J* = 5.0, 8.5 Hz, 1H), 7.48–7.39 (m, 2H), 7.22 (dd, *J* = 2.1, 8.5 Hz, 1H), 7.09–7.01 (m, 1H), 3.24–3.08 (m, 2H), 2.99 (t, *J* = 7.3 Hz, 2H), 2.77 (dd, *J* = 7.6, 9.4 Hz, 1H), 2.68–2.46 (m, 5H), 2.43 (s, 3H), 2.39 (dd, *J* = 6.4, 9.4 Hz, 1H), 2.07–1.94 (m, 3H), 1.52 (qd, *J* = 6.8, 13.5 Hz, 1H).^13^C NMR (75 MHz, CDCl_3_, δ): 163.6 (d, *J* = 13.8 Hz), 164.2 (d, *J* = 251.0 Hz), 158.2, 139.6, 139.4, 134.5, 129.2, 128.2, 125.1, 122.2 (d, *J* = 11.5 Hz), 118.3, 112.4 (d, *J* = 25.3 Hz), 97.3 (d, *J* = 26.5 Hz), 61.3, 59.3, 55.0, 53.2, 31.8, 30.5, 26.5, 22.9, 21.3. LC-MS (ESI) calcd for C_22_H_25_FN_2_O_3_S 416.51 [M + H^+^], found 417 [M + H^+^].

#### General procedure for the preparation of final molecules **13–22**

Cesium carbonate (2.0 equiv), a catalytic amount of DMAP, and appropriate arylsulfonyl chloride (1.2 equiv) were added to a suspension of (3*S*,5*S*)-(1-(3-(6-fluorobenzo[*d*]isoxazol-3-yl)propyl)-5-methylpyrrolidin-3-amine hydrochloride (**VI**) (1 equiv) in dry dichloromethane at room temperature. The reaction mixture was stirred for 12 h, then the cesium carbonate was filtered, and the solvent was evaporated under reduced pressure. Crude reaction mixtures were purified by flash column chromatography over silica gel using dichloromethane/methanol 90:10 (v/v) as eluent.

##### 3-Chloro-*N*-((3*S*,5*S*)-1-(3-(6-fluorobenzo[*d*]isoxazol-3-yl)propyl)-5-methylpyrrolidin-3-yl)benzenesulfonamide (**13**)

The title compound was prepared using (3*S*,5*S*)-*tert*-(1-(3-(6-fluorobenzo[*d*]isoxazol-3-yl)propyl)-5-methylpyrrolidin-3-amine hydrochloride **VI** (0.44 mmol, 1 equiv, 0.137 g), 3-chlorobenzene-1-sulfonyl chloride (0.53 mmol, 1.2 equiv, 0.112 g), DMAP (catalytic amount), and cesium carbonate (0.88 mmol, 2 equiv, 0.288 g) in DCM (5 mL). Yield 48%, yellowish oil. ^1^H NMR (300 MHz, CDCl_3_, δ): 7.86 (t, *J* = 2.1 Hz, 1H), 7.74 (td, *J* = 1.3, 7.9 Hz, 1H), 7.61–7.51 (m, 2H), 7.47–7.42 (m, 1H), 7.25 (dd, *J* = 2.1, 8.5 Hz, 1H), 7.07 (dt, *J* = 2.3, 8.8 Hz, 1H), 3.77 (quin, *J* = 7.03 Hz, 1 H), 3.35–3.25 (m, 1 H), 3.04–2.86 (m, 2 H), 2.78 (dt, *J* = 12.16 Hz, 2 H), 2.66–2.58 (m, 1 H), 2.30–2.19 (m, 1 H), 2.12–1.89 (m, 3 H), 1.75–1.62 (m, 2 H), 0.96 (d, *J* = 5.86 Hz, 3 H). ^13^C NMR (75 MHz, CDCl_3_, δ): 165.9 (d, *J* = 13.8 Hz), 163.5 (d, *J* = 250.2 Hz), 158.1, 142.6, 135.3, 132.7, 130.4, 127.1, 125.1, 122.1 (d, *J* = 10.4 Hz), 118.2 (d, *J* = 1.7 Hz), 112.5 (d, *J* = 26.0 Hz), 97.4 (d, *J* = 27.0 Hz), 60.5, 54.5, 52.9, 51.8, 32.4, 26.1, 22.8, 18.32. LC-MS (ESI) calcd for C_21_H_23_ClFN_3_O_3_S 451.94 [M + H^+^], found 452 [M + H^+^].

##### 3-Chloro-4-fluoro-*N*-((3*S*,5*S*)-1-(3-(6-fluorobenzo[*d*]isoxazol-3-yl)propyl)-5-methylpyrrolidin-3-yl)benzenesulfonamide (**14**)

The title compound was prepared using (3*S*,5*S*)-*tert*-(1-(3-(6-fluorobenzo[*d*]isoxazol-3-yl)propyl)-5-methylpyrrolidin-3-amine hydrochloride **VI** (0.44 mmol, 1 equiv, 0.137 g), 3-chloro-4-fluorobenzene-1-sulfonyl chloride (0.528 mmol, 1.2 equiv, 0.120 g), DMAP (catalytic amount), and cesium carbonate (0.88 mmol, 2 equiv, 0.288 g) in DCM (5 mL). Yield 52%, yellowish oil. ^1^H NMR (300 MHz, CDCl_3_, δ): 7.98–7.90(m, 1H), 7.77 (ddd, *J* = 8.65, 4.25, 2.34 Hz, 1H), 7.58 (dd, *J* = 8.79, 5.27 Hz, 1H), 7.32–7.19 (m, 2H), 7.06 (dt, *J* = 2.3, 8.8 Hz, 1H), 3.83–3.59 (m, 2H), 3.56–3.40 (m, 1H), 3.35–3.23 (m, 1H), 3.21–3.12 (m, 1H), 2.98 (dq, *J* = 15.53, 7.62 Hz, 2H), 2.86–2.58 (m, 2H), 2.336–2.24 (m, 1H), 2.20–1.91 (m, 3H), 1.86–1.59 (m, 2H), 1.00–0.96 (m, 3H). ^13^C NMR (75 MHz, CDCl_3_, δ): 165.9 (d, *J* = 13.8 Hz), 163.5 (d, *J* = 250.2 Hz), 162.0, 158.1, 142.6, 135.3, 132.7, 130.4, 127.1, 125.1, 122.1 (d, *J* = 10.4 Hz), 118.2 (d, *J* = 1.7 Hz), 112.5 (d, *J* = 26.0 Hz), 97.4 (d, *J* = 27.0 Hz), 60.5, 54.5, 52.9, 51.8, 32.4, 26.1, 22.8, 18.32. LC-MS (ESI) calcd for C_21_H_22_ClF_2_N_3_O_3_S 469.93 [M + H^+^], found 469 [M + H^+^].

##### *N*-((3*S*,5*S*)-1-(3-(6-Fluorobenzo[*d*]isoxazol-3-yl)propyl)-5-methylpyrrolidin-3-yl)benzo[*b*]thiophene-3-sulfonamide (**15**)

The title compound was prepared using (3*S*,5*S*)-*tert*-(1-(3-(6-fluorobenzo[*d*]isoxazol-3-yl)propyl)-5-methylpyrrolidin-3-amine hydrochloride **VI** (0.44 mmol, 1 equiv, 0.137 g), benzo[*b*]thiophene-3-sulfonyl chloride (0.528 mmol, 1.2 equiv, 0.122 g), DMAP (catalytic amount), and cesium carbonate (0.88 mmol, 2 equiv, 0.288 g) in DCM (5 mL). Yield 79%, yellowish oil, chiral HPLC ee > 99%. ^1^H NMR (300 MHz, CDCl_3_, δ): 8.23 (s, 1H), 8.15 (d, *J* = 8.2 Hz, 1H), 7.87 (d, *J* = 8.8 Hz, 1H), 7.55 (dd, *J* = 5.3, 8.8 Hz, 1H), 7.47 – 7.35 (m, 2H), 7.25 7.22 (m, 1H), 7.06 (dt, *J* = 2.1, 8.9 Hz, 1H), 3.99–3.84 (m, 1H), 3.36 (dd, *J* = 9.67, 6.74 Hz, 2H), 3.03–2.50 (m, 4H), 2.25 (ddd, *J* = 12.16, 7.18, 5.27 Hz, 2H), 2.15–2.05 (m, 3H), 2.00–1.86 (m, 2H), 0.96 (d, *J* = 5.86 Hz, 2H). ^19^F NMR (282 MHz, CDCl_3_, δ): −109.6 (s, 1F); ^13^C NMR (75 MHz, CDCl_3_, δ): 165.9 (d, *J* = 250.2 Hz), 163.5 (d, *J* = 13.8 Hz), 157.8, 140.2, 134.3, 133.8, 125.7, 122.9, 122.1 (d, *J* = 11.5 Hz), 118.2, 112.5 (d, *J* = 26.0 Hz), 110.0, 97.5 (d, *J* = 26.5 Hz), 97.1, 60.2, 54.5, 52.8, 51.9, 32.3, 29.7, 25.9, 22.8, 18.22. LC-MS (ESI) calcd for C_23_H_24_FN_3_O_3_S_2_ 473.58 [M + H^+^], found 474 [M + H^+^].

##### *N*-((3*S*,5*S*)-1-(3-(6-Fluorobenzo[*d*]isoxazol-3-yl)propyl)-5-methylpyrrolidin-3-yl)benzo[*b*]thiophene-2-sulfonamide (**16**)

The title compound was prepared using (3*S*,5*S*)-*tert*-(1-(3-(6-fluorobenzo[*d*]isoxazol-3-yl)propyl)-5-methylpyrrolidin-3-amine hydrochloride **VI** (0.44 mmol, 1 equiv, 0.137 g), benzo[*b*]thiophene-2-sulfonyl chloride (0.528 mmol, 1.2 equiv, 0.122 g), DMAP (catalytic amount), and cesium carbonate (0.67 mmol, 2 equiv, 0.288 g) in DCM (5 mL). Yield 87%, yellowish oil, chiral HPLC ee > 99%. ^1^H NMR (300 MHz, CDCl_3_, δ): 8.25 (s, 1H), 8.16 (d, *J* = 8.2 Hz, 1H), 7.90 (d, *J* = 8.8 Hz, 1H), 7.49–7.35 (m, 3H), 7.26–7.22 (m, 1H), 7.04 (dt, *J* = 2.1, 8.9 Hz, 1H), 3.99–3.82 (m, 1H), 3.21 (dd, *J* = 9.67, 6.74 Hz, 2H), 3.03–2.92 (m, 4H), 2.73 (ddd, *J* = 12.16, 7.18, 5.27 Hz, 2H), 2.19–2.05 (m, 3H), 2.00–1.91 (m, 2H), 0.89 (d, *J* = 5.86 Hz, 2H). ^19^F NMR (282 MHz, CDCl_3_, δ): −109.6 (s, 1F); ^13^C NMR (75 MHz, CDCl_3_, δ): 165.9 (d, *J* = 250.2 Hz), 163.5 (d, *J* = 13.8 Hz), 157.8, 140.2, 134.3, 133.8, 125.7, 122.9, 122.1 (d, *J* = 11.5 Hz), 118.2, 112.5 (d, *J* = 26.0 Hz), 110.0, 97.5 (d, *J* = 26.5 Hz), 97.1, 60.2, 54.5, 52.8, 51.9, 32.3, 29.7, 25.9, 22.8, 18.22. LC-MS (ESI) calcd for C_23_H_24_FN_3_O_3_S_2_ 473.58 [M + H^+^], found 474 [M + H^+^].

##### *N*-((3*S*,5*S*)-1-(3-(6-Fluorobenzo[*d*]isoxazol-3-yl)propyl)-5-methylpyrrolidin-3-yl)benzofuran-2-sulfonamide (**17**)

The title compound was prepared using (3*S*,5*S*)-*tert*-(1-(3-(6-fluorobenzo[*d*]isoxazol-3-yl)propyl)-5-methylpyrrolidin-3-amine hydrochloride **VI** (0.44 mmol, 1 equiv, 0.137 g), benzofuran-2-sulfonyl chloride (0.528 mmol, 1.2 equiv, 0.113 g), DMAP (catalytic amount), and cesium carbonate (0.88 mmol, 2 equiv, 0.288 g) in DCM (5 mL). Yield 66%, yellowish oil. ^1^H NMR (300 MHz, CDCl_3_, δ): 8.62–8.53 (m, 1H), 7.69–7.63 (m, 1H), 7.61–7.03 (m, 6H), 4.18–3.90 (m, 1H), 3.68–3.37 (m, 2H), 3.34 (br s, 1H), 3.28–3.13 (m, 1H), 3.09–2.84 (m, 1H), 2.84–2.73 (m, 1H), 2.48–2.15 (m, 3H), 2.05 (dt, *J* = 14.51, 7.11 Hz, 2H), 1.43–1.13 (m, 1H), 1.08–1.06 (m, 3H); ^13^C NMR (75 MHz, CDCl_3_, δ): 165.9 (d, *J* = 250.2 Hz), 163.6 (d, *J* = 13.8 Hz), 162.6, 158.1, 155.6, 150.6, 127.6, 126.0, 124.2, 122.9, 122.2 (d, *J* = 11.5 Hz), 118.2, 112.5 (d, *J* = 25.4 Hz), 112.1, 97.5 (d, *J* = 26.5 Hz), 60.5, 54.6, 53.1, 52.0, 32.5, 26.1, 22.9, 18.9. LC-MS (ESI) calcd for C_23_H_24_FN_3_O_4_S 457.51 [M + H^+^], found 458 [M + H^+^].

##### 6-Fluoro-*N*-((3*S*,5*S*)-1-(3-(6-fluorobenzo[*d*]isoxazol-3-yl)propyl)-5-methylpyrrolidin-3-yl)benzo[*b*]thiophene-2-sulfonamide (**18**)

The title compound was prepared using (3*S*,5*S*)-*tert*-(1-(3-(6-fluorobenzo[*d*]isoxazol-3-yl)propyl)-5-methylpyrrolidin-3-amine hydrochloride **VI** (0.44 mmol, 1 equiv, 0.137 g), 6-fluorobenzo[*b*]thiophene-2-sulfonyl chloride (0.528 mmol, 1.2 equiv, 0.131 g), DMAP (catalytic amount), and cesium carbonate (0.88 mmol, 2 equiv, 0.288 g) in DCM (5 mL). Yield 69%, yellowish oil. ^1^H NMR (300 MHz, CDCl_3_, δ): 7.85–7.79 (m, 2H), 7.58–7.49 (m, 2H), 7.26–7.16 (m, 2H), 7.07–7.00 (m, 1H), 3.94–3.85 (m, 1H), 3.38–3.32 (m, 1H), 3.04–2.85 (m, 2H), 2.78 (t, *J* = 7.6 Hz, 2H), 2.64–2.54 (m, 1H), 2.26 (ddd, *J* = 2.9, 10.0 Hz, 1H), 2.13 (dd, *J* = 9.96, 7.03 Hz, 1H), 2.00–1.87 (m, 2H), 1.84–1.68 (m, 2H), 0.98-0.96 (m, 3H); ^13^C NMR (75 MHz, CDCl_3_, δ): 171.8 (d, *J* = 250.2 Hz), 163.6 (d, *J* = 13.8 Hz), 162.6 (d, *J* = 249.0 Hz), 158.1, 154.0 (d, *J* = 2.2 Hz), 137.6, 128.7, 127.1, 126.9 (d, *J* = 9.3 Hz), 122.0 (d, *J* = 11.5 Hz), 118.2, 115.1 (d, *J* = 24.3 Hz), 112.5 (d, *J* = 26.0 Hz), 108.8 (d, *J* = 25.2 Hz), 97.4 (d, *J* = 26.5 Hz), 60.4, 54.5, 53.2, 51.9, 32.4, 26.2, 22.9, 18.4. LC-MS (ESI) calcd for C_23_H_23_F_2_N_3_O_3_S_2_ 491.57 [M + H^+^], found 492 [M + H^+^].

##### 6-Chloro-*N*-((3*S*,5*S*)-1-(3-(6-fluorobenzo[*d*]isoxazol-3-yl)propyl)-5-methylpyrrolidin-3-yl)benzo[*b*]thiophene-2-sulfonamide (**19**)

The title compound was prepared using (3*S*,5*S*)-*tert*-(1-(3-(6-fluorobenzo[*d*]isoxazol-3-yl)propyl)-5-methylpyrrolidin-3-amine hydrochloride **VI** (0.44 mmol, 1 equiv, 0.137 g), 6-chlorobenzo[*b*]thiophene-2-sulfonyl chloride (0.528 mmol, 1.2 equiv, 0.140 g), DMAP (catalytic amount), and cesium carbonate (0.88 mmol, 2 equiv, 0.288 g) in DCM (5 mL). Yield 71%, yellowish oil. ^1^H NMR (300 MHz, CDCl_3_, δ): 7.84–7.75 (m, 2H), 7.56 (dd, *J* = 8.50, 4.98 Hz, 1H), 7.41 (dd, *J* = 8.79, 1.76 Hz, 1H), 7.26–7.16 (m, 2H), 7.07–7.00 (m, 1H), 3.95–3.86 (m, 1H), 3.49–3.31 (m, 1H), 3.04–2.86 (m, 2H), 2.83–2.72 (m, 2H), 2.66–2.55 (m, 1H), 2.31–2.10 (m, 1H), 2.03–1.87 (m, 2H), 1.82–1.68 (m, 2H), 0.98-0.96 (m, 4H); ^13^C NMR (75 MHz, CDCl_3_, δ): 171.8 (d, *J* = 250.2 Hz), 163.6 (d, *J* = 13.8 Hz), 162.6 (d, *J* = 249.0 Hz), 158.1, 154.0 (d, *J* = 2.2 Hz), 137.6, 128.7, 127.1, 126.9 (d, *J* = 9.3 Hz), 122.0 (d, *J* = 11.5 Hz), 118.2, 115.1 (d, *J* = 24.3 Hz), 112.5 (d, *J* = 26.0 Hz), 108.8 (d, *J* = 25.2 Hz), 97.4 (d, *J* = 26.5 Hz), 60.4, 54.5, 53.2, 51.9, 32.4, 26.2, 22.9, 18.4. LC-MS (ESI) calcd for C_23_H_23_ClFN_3_O_3_S_2_ 508.02 [M + H^+^], found 509 [M + H^+^].

##### 5-Chloro-*N*-((3*S*,5*S*)-1-(3-(6-fluorobenzo[*d*]isoxazol-3-yl)propyl)-5-methylpyrrolidin-3-yl)-3-methylbenzo[*b*]thiophene-2-sulfonamide (**20**)

The title compound was prepared using (3*S*,5*S*)-*tert*-(1-(3-(6-fluorobenzo[*d*]isoxazol-3-yl)propyl)-5-methylpyrrolidin-3-amine hydrochloride **VI** (0.44 mmol, 1 equiv, 0.137 g), 5-chloro-3-methylbenzo[*b*]thiophene-2-sulfonyl chloride (0.528 mmol, 1.2 equiv, 0.148 g), DMAP (catalytic amount), and cesium carbonate (0.88 mmol, 2 equiv, 0.288 g) in DCM (5 mL). Yield 92%, yellowish oil. ^1^H NMR (300 MHz, CDCl_3_, δ): 7.78–7.72 (m, 2H), 7.55 (dd, *J* = 5.3, 8.8 Hz, 1H), 7.42 (dd, *J* = 2.1, 8.5 Hz, 1H), 7.19 (dd, *J* = 1.8, 8.8 Hz, 1H), 7.07–7.01 (m, 1H), 3.94 – 3.92 (m, 1H), 3.36–3.30 (m, 1H), 3.33 (dd, *J* = 9.67, 6.74 Hz, 2H), 3.03–2.90 (m, 2H), 2.73 (ddd, *J* = 12.16, 7.18, 5.27 Hz, 2H), 2.63 (s, 3H), 2.12–2.08 (m, 1H), 1.96–1.92 (m, 2H), 1.74–1.70 (m, 2H), 0.95 (d, *J* = 5.86 Hz, 3H). ^19^F NMR (282 MHz, CDCl_3_, δ): −109.6 (s, 1F); ^13^C NMR (75 MHz, CDCl_3_, δ): 165.9 (d, *J* = 250.2 Hz), 163.5 (d, *J* = 13.8 Hz), 158.0, 140.8, 137.5, 135.8, 131.5, 127.8, 123.7, 123.3 (d, *J* = 11.5 Hz), 118.2, 112.5 (d, *J* = 26.0 Hz), 122.2, 97.5 (d, *J* = 26.5 Hz), 97.1, 59.8, 57.8, 51.9, 51.04, 40.5, 26.0, 22.9;18.35, 12.4. LC-MS (ESI) calcd for C_24_H_25_ClFN_3_O_3_S_2_ 521,10 [M + H^+^], found 522 [M + H^+^].

##### *N*-((3*S*,5*S*)-1-(3-(6-Fluorobenzo[*d*]isoxazol-3-yl)propyl)-5-methylpyrrolidin-3-yl)naphthalene-2-sulfonamide (**21**)

The title compound was prepared using (3*S*,5*S*)-*tert*-(1-(3-(6-fluorobenzo[*d*]isoxazol-3-yl)propyl)-5-methylpyrrolidin-3-amine hydrochloride **VI** (0.44 mmol, 1 equiv, 0.137 g), naphthalene-2-sulfonyl chloride (0.58 mmol, 1.2 equiv, 0.118 g), DMAP (catalytic amount), and cesium carbonate (0.88 mmol, 2 equiv, 0.288 g) in DCM (5 mL). Yield 95%, yellowish oil, chiral HPLC ee > 99%. ^1^H NMR (300 MHz, CDCl_3_, δ): 8.44 (s, 1H), 7.95–7.83 (m, 1H), 7.65–7.49 (m, 1H), 7.19 (d, *J* = 1.76 Hz, 2H), 7.16 (d, *J* = 2.34 Hz, 1H), 7.03 (d, *J* = 1.76 Hz, 1H), 7.00 (d, *J* = 1.76 Hz, 2H), 6.97 (d, *J* = 1.76 Hz, 1H), 3.85–3.79 (m, 1H), 3.33–3.29 (m, 1H), 2.97–2.86 (m, 2H), 2.84–2.72 (m, 1H), 2.09–2.06 (m, 2H), 2.14 (dd, *J* = 9.67, 7.33 Hz, 1H), 1.96–1.86 (m, 2H), 1.78–1.59 (m, 2H), 1.25–1.19 (m, 1H), 0.93 (d, *J* = 6.45 Hz, 3H); ^13^C NMR (75 MHz, CDCl_3_, δ): 165.8 (d, *J* = 250.2 Hz), 163.5 (d, *J* = 13.8 Hz), 158.0, 137.5, 135.3, 132.9, 130.7, 130.4, 128.6, 128.1, 126.7, 123.5, 122.1 (d, *J* = 11.5 Hz), 118.1, 112.5 (d, *J* = 26.0 Hz), 97.4 (d, *J* = 26.5 Hz), 59.8, 57.9, 52.0, 50.7, 40.46, 29.7, 26.0, 22.9, 18.4. LC-MS (ESI) calcd for C_25_H_26_FN_3_O_3_S 467.55 [M + H^+^], found 468 [M + H^+^].

##### 6-Chloro-*N*-((3*S*,5*S*)-1-(3-(6-fluorobenzo[*d*]isoxazol-3-yl)propyl)-5-methylpyrrolidin-3-yl)naphthalene-2-sulfonamide (**22**)

The title compound was prepared using (3*S*,5*S*)-*tert*-(1-(3-(6-fluorobenzo[*d*]isoxazol-3-yl)propyl)-5-methylpyrrolidin-3-amine hydrochloride **VI** (0.44 mmol, 1 equiv, 0.137 g), 6-chloronaphthalene-2-sulfonyl chloride (0.528 mmol, 1.2 equiv, 0.137 g), DMAP (catalytic amount), and cesium carbonate (0.88 mmol, 2 equiv, 0.288 g) in DCM (5 mL). Yield 91%, yellowish oil. ^1^H NMR (300 MHz, CDCl_3_, δ): 8.41 (s, 1H), 7.90–7.80 (m, 4H), 7.54–7.50 (m, 2H), 7.20–7.17 (m, 1H), 7.02–6.98 (m, 1H), 3.81–3.79 (m, 1H), 3.29–3.23 (m, 1H), 2.94–2.86 (m, 2H), 2.75–2.71 (m, 1H), 2.56–2.54 (m, 1H), 2.09–2.06 (m, 2H), 1.96–1.92 (m, 2H), 1.74–1.70 (m, 2H), 1.27–1.17 (m, 1H), 0.95 (d, *J* = 5.86 Hz, 3H); ^13^C NMR (75 MHz, CDCl_3_, δ): 165.8 (d, *J* = 250.2 Hz), 163.5 (d, *J* = 13.8 Hz), 158.0, 137.5, 135.3, 132.9, 130.7, 130.4, 128.6, 128.1, 126.7, 123.5, 122.1 (d, *J* = 11.5 Hz), 118.1, 112.5 (d, *J* = 26.0 Hz), 97.4 (d, *J* = 26.5 Hz), 59.8, 57.9, 52.0, 50.7, 40.46, 29.7, 26.0, 22.9, 18.4. LC-MS (ESI) calcd for C_25_H_25_ClFN_3_O_3_S 502.0 [M + H^+^], found 503 [M + H^+^].

### *In Vitro* Studies

The tested compounds were examined for known classes of assay interference compounds. None of the compounds contains substructural features recognized as pan assay interference compounds (PAINS), according to the SwissADME tool.^79^

### Radioligand *Binding* Studies

#### Preparation of Solutions of Test and Reference Compounds

Stock solutions (10 mM) of tested compounds were prepared in 100% dimethyl sulfoxide (DMSO). Serial dilutions of tested compounds were prepared in 96-well microplate in assay buffers using an automated pipetting system epMotion 5070 (Eppendorf). Each compound was tested in six concentrations from 10^–5^ to 10^–10^ M (final concentration).

### 5-HT_2A_ Receptor Binding Assay

Radioligand binding assay was performed using membranes from CHO-K1 cells stably transfected with the human 5-HT_2A_ receptor provided by PerkinElmer. All tests were carried out in duplicate. First, 50 μL of working solution of the tested compounds, 50 μL of [3*H*]-ketanserin (final concentration 1 nM), and 150 μL of diluted membranes (7 μg protein per well) prepared in assay buffer (50 mM Tris, pH 7.4, 4 mM CaCl_2_, 0.1% ascorbic acid) were transferred to a polypropylene 96-well microplate using a Rainin Liquidator (Mettler Toledo) 96-well pipetting station. Mianserin (10 μM) was used to define nonspecific binding. The microplate was covered with sealing tape, mixed, and incubated for 60 min at 27 °C. The reaction was stopped by fast filtration through a GF/B filter mat presoaked with 0.5% polyethylenimine for 30 min. Ten rapid washes with 200 μL of 50 mM Tris buffer (4 °C, pH 7.4) were performed using a Harvester-96 MACH III FM automated harvester system (Tomtec). The filter mats were dried at 37 °C in a forced air fan incubator, and then solid scintillator MeltiLex was melted onto the filter mats at 90 °C for 5 min. Radioactivity was measured in a MicroBeta2 scintillation counter (PerkinElmer). Data were fitted to a one-site curve-fitting equation with Prism 6 (GraphPad Software), and *K*_i_ values were estimated from the Cheng–Prusoff equation.^80^

### 5-HT_6_ Receptor Binding Assay

Radioligand binding was performed using membranes from CHO-K1 cells stably transfected with the human 5-HT_6_ receptor (PerkinElmer). All assays were carried out in duplicate. First, 50 μL of working solution of the tested compounds, 50 μL of [3*H*]-LSD (final concentration 1 nM), and 150 μL of diluted membranes (8 μg protein per well) prepared in assay buffer (50 mM Tris, pH 7.4, 10 mM MgCl_2_, 0.1 mM ethylenediamine tetraacetic acid (EDTA)) were transferred to a polypropylene 96-well microplate using a Rainin Liquidator 96-well pipetting station (Mettler Toledo). Methiothepin (10 μM) was used to define nonspecific binding. The microplate was covered with sealing tape, mixed, and incubated for 60 min at 37 °C. The reaction was completed by rapid filtration through a GF/A filter mat presoaked with 0.5% polyethylenimine for 30 min. Ten rapid washes with 200 μL of 50 mM Tris buffer (4 °C, pH 7.4) were performed using a Harvester-96 MACH III FM automated harvester system (Tomtec). The filter mats were dried at 37 °C in a forced air fan incubator, and then solid scintillator MeltiLex was melted onto the filter mats at 90 °C for 5 min. Radioactivity was counted in a MicroBeta2 scintillation counter (PerkinElmer). Data were fitted to a one-site curve-fitting equation with Prism 6 (GraphPad Software), and *K*_i_ values were estimated from the Cheng–Prusoff equation.^80^

### 5-HT_7_ Receptor Binding Assay

Radioligand binding was performed using membranes from CHO-K1 cells stably transfected with the human 5-HT7 receptor (PerkinElmer). All assays were carried out in duplicate. First, 50 μL of working solution of the tested compounds, 50 μL of [3*H*]-LSD (final concentration 3 nM), and 150 μL of diluted membranes (17 μg protein per well) prepared in assay buffer (50 mM Tris, pH 7.4, 10 mM MgSO_4_, 0.5 mM EDTA) were transferred to a polypropylene 96-well microplate using a Rainin Liquidator 96-well pipetting station (Mettler Toledo). Methiothepin (10 μM) was used to define nonspecific binding. The microplate was covered with sealing tape, mixed, and incubated for 120 min at 27 °C. The reaction was terminated by rapid filtration through a GF/A filter mat presoaked with 0.3% polyethylenimine for 30 min. Ten rapid washes with 200 μL of 50 mM Tris buffer (4 °C, pH 7.4) were performed using a Harvester-96 MACH III FM an automated harvester system (Tomtec). The filter mats were dried at 37 °C in a forced air fan incubator, and then solid scintillator MeltiLex was melted onto the filter mats at 90 °C for 5 min. Radioactivity was counted in a MicroBeta2 scintillation counter (PerkinElmer). Data were fitted to a one-site curve-fitting equation with Prism 6 (GraphPad Software), and *K*_i_ values were estimated from the Cheng–Prusoff equation.^80^

### D_2_ Receptor Binding Assay

Radioligand binding was performed using membranes from CHO-K1 cells stably transfected with the human D_2_ receptor (PerkinElmer). All assays were carried out in duplicate. First, 50 μL of working solution of the tested compounds, 50 μL of [3*H*]-methylspiperone (final concentration 0.4 nM), and 150 μL of diluted membranes (3 μg protein per well) prepared in assay buffer (50 mM Tris, pH 7.4, 50 mM HEPES, 50 mM NaCl, 5 mM MgCl_2_, 0.5 mM EDTA) were transferred to a polypropylene 96-well microplate using a Rainin Liquidator 96-well pipetting station (Mettler Toledo). Haloperidol (10 μM) was used to define nonspecific binding. The microplate was covered with sealing tape, mixed, and incubated for 60 min at 37 °C. The reaction was terminated by rapid filtration through a GF/B filter mat presoaked with 0.5% polyethylenimine for 30 min. Ten rapid washes with 200 μL of 50 mM Tris buffer (4 °C, pH 7.4) were performed using a Harvester-96 MACH III FM automated harvester system (Tomtec). The filter mats were dried at 37 °C in a forced air fan incubator, and then solid scintillator MeltiLex was melted onto the filter mats at 90 °C for 5 min. Radioactivity was counted in a MicroBeta2 scintillation counter (PerkinElmer). Data were fitted to a one-site curve-fitting equation with Prism 6 (GraphPad Software), and *K*_i_ values were estimated from the Cheng–Prusoff equation.^80^

### M_1_ Receptor Binding Assay

Radioligand binding was performed using membranes from CHO-K1 cells stably transfected with the human M_1_ receptor (PerkinElmer). All assays were carried out in duplicate. The working solution (50 μL) of the tested compounds, [^3^H]-Scopolamine (50 μL) (final concentration 0.14 nM), and diluted membranes (150 μL) (35 μg protein per well) prepared in PBS (pH 7.4) were transferred to a polypropylene 96-well microplate using a Rainin Liquidator 96-well pipetting station (Mettler Toledo). Pirenzepine (10 μM) was used to define nonspecific binding. The microplate was covered with sealing tape, mixed, and incubated for 120 min at 27 °C. The reaction was terminated by rapid filtration through a GF/B filter mat presoaked with 0.3% polyethyleneimine for 30 min. Ten rapid washes with 200 μL of 50 mM Tris buffer, 154 mM NaCl (4 °C, pH 7.4) were performed using a 96-well FilterMate harvester (PerkinElmer). The filter mats were dried at 37 °C in a forced air fan incubator and then solid scintillator MeltiLex was melted onto the filter mats at 90 °C for 4 min. The radioactivity on the filters was measured in a MicroBeta TriLux 1450 scintillation counter (PerkinElmer). Data were fitted to a one-site curve-fitting equation with Prism 6 (GraphPad Software). Results from the assay are represented as a percentage of [^3^H]-scopolamine inhibition by tested compounds.

### H_1_ Receptor Binding Assay

Radioligand binding was performed using membranes from CHO-K1 cells stably transfected with the human H_1_ receptor (PerkinElmer). All assays were carried out in duplicate. The working solution of the tested compounds (50 μL), [^3^H]-Pyrilamine (50 μL) (final concentration 1.5 nM), and diluted membranes (150 μL) (5 μg protein per well) prepared in assay buffer (50 mM Tris, pH 7.4, 5 mM MgCl_2_) were transferred to a polypropylene 96-well microplate using a Rainin Liquidator 96-well pipetting station (Mettler Toledo). Mepyramine (10 μM) was used to define nonspecific binding. The microplate was covered with sealing tape, mixed, and incubated for 60 min at 27 °C. The reaction was terminated by rapid filtration through a GF/B filter mat presoaked with 0.5% polyethyleneimine for 30 min. Ten rapid washes with 200 μL of 50 mM Tris buffer (4 °C, pH 7.4) were performed using a 96-well FilterMate harvester (PerkinElmer). The filter mats were dried at 37 °C in a forced air fan incubator and then solid scintillator MeltiLex was melted onto the filter mats at 90 °C for 4 min. The radioactivity on the filters was measured in a MicroBeta TriLux 1450 scintillation counter (PerkinElmer). Data were fitted to a one-site curve-fitting equation with Prism 6 (GraphPad Software). Results from the assay are represented as a percentage of [^3^H]-Pyrilamine inhibition by tested compounds.

### α_1_-Adrenergic Receptor Binding Assay

Radioligand binding was performed using rat cortex tissue. All assays were carried out in duplicate. The working solution of the tested compounds (50 μL), [^3^H]-prazosin (50 μL) (final concentration 0.3 nM), and tissue suspension prepared in assay buffer (150 μL) (50 mM Tris-HCl, pH 7.6) were transferred to a polypropylene 96-well microplate using a Rainin Liquidator 96-well pipetting station (Mettler Toledo). Phentolamine (10 μM) was used to define nonspecific binding. The microplate was covered with sealing tape, mixed, and incubated for 30 min at 30 °C. The reaction was terminated by rapid filtration through a GF/B filter mat. Ten rapid washes with 200 μL of 50 mM Tris buffer (4 °C, pH 7.6) were performed using a 96-well FilterMate harvester (PerkinElmer). The filter mats were dried at 37 °C in a forced air fan incubator and then solid scintillator MeltiLex was melted onto the filter mats at 90 °C for 4 min. The radioactivity on the filter was measured in a MicroBeta TriLux 1450 scintillation counter (PerkinElmer). Data were fitted to a one-site curve-fitting equation with Prism 6 (GraphPad Software). Results from the assay are represented as a percentage of [^3^H]-prazosin inhibition by tested compounds.^81^

### Functional Assay for the 5-HT_2A_ Receptor

Test and reference compounds were dissolved in dimethyl sulfoxide (DMSO) at a concentration of 1 mM. Serial dilutions were prepared in a 96-well microplate in assay buffer and 8 to 10 different concentrations of each compound were tested. A cellular aequorin-based functional assay was performed with recombinant CHO-K1 cells expressing mitochondrially targeted aequorin, human GPCR, and the promiscuous G protein α16 for 5-HT2A. The assay was executed according to the previously described protocol. After thawing, the cells were transferred to assay buffer (DMEM/HAM’s F12 with 0.1% protease-free bovine serum albumin (BSA)) and centrifuged. The cell pellet was resuspended in assay buffer and coelenterazine h was added at a final concentration of 5 μM. The cell suspension was incubated at 16 °C, protected from light, with constant agitation for 16 h and then diluted with assay buffer to a concentration of 100 000 cells/mL. After 1 h of incubation, 50 μL of the cell suspension was dispensed using automatic injectors built into the MicroBeta2 LumiJET radiometric and luminescence plate counter (PerkinElmer) into white opaque 96-well microplates preloaded with test compounds. Immediate light emission generated following calcium mobilization was recorded for 30 s. In the antagonist mode, after 30 min of incubation, the reference agonist was added to the above assay mix and light emission was recorded again. The final concentration of the reference agonist was equal to EC80 (30 nM serotonin).

### Functional Assay for the 5-HT_6_ Receptor

Test and reference compounds were dissolved in dimethyl sulfoxide (DMSO) at a concentration of 1 mM. Serial dilutions were prepared in a 96-well microplate in assay buffer and 8 to 10 different concentrations of each compound were tested. A cellular aequorin-based functional assay was performed with recombinant CHO-K1 cells expressing mitochondrially targeted aequorin, human GPCR, and the promiscuous G protein α16 for 5-HT6. The assay was executed according to the previously described protocol. After thawing, the cells were transferred to assay buffer (DMEM/HAM’s F12 with 0.1% protease-free BSA) and centrifuged. The cell pellet was resuspended in assay buffer and coelenterazine h was added at a final concentration of 5 μM. The cell suspension was incubated at 16 °C, protected from light, with constant agitation for 16 h and then diluted with assay buffer to a concentration of 100 000 cells/mL. After 1 h of incubation, 50 μL of the cell suspension was dispensed using the automatic injectors built into the MicroBeta2 LumiJET radiometric and luminescence plate counter (PerkinElmer) into white opaque 96-well microplates preloaded with test compounds. Immediate light emission generated following calcium mobilization was recorded for 60 s. In the antagonist mode, after 30 min of incubation, the reference agonist was added to the above assay mix and light emission was recorded again. The final concentration of the reference agonist was equal to EC80 (40 nM serotonin).

### Functional Assay for the 5-HT_7_ Receptor

Test and reference compounds were dissolved in dimethyl sulfoxide (DMSO) at a concentration of 1 mM. Serial dilutions were prepared in a 96-well microplate in assay buffer and 8 to 10 different concentrations of each compound were tested. For the 5-HT_7_, adenylyl cyclase activity was monitored using cryopreserved CHO-K1 cells expressing the human serotonin 5-HT_7_ receptor. A functional assay based on cells expressing the human 5-hydroxytryptamine (serotonin) receptor 7 was performed. CHO-K1 cells were transfected with a β-lactamase reporter gene under the control of the cyclic AMP response element (CRE) (Life Technologies). Thawed cells were resuspended in stimulation buffer (HBSS, 5 mM HEPES, 0.5 IBMX, and 0.1% BSA at pH 7.4) at 2 × 10^5^ cells/mL for the 5-HT_7_ receptor. The same volume (10 μL) of cell suspension was added to tested compounds for the 5-HT7 receptor. Samples were loaded onto a white opaque half-area 96-well microplate. The antagonist response experiment was performed with 10 nM serotonin as the reference agonist for the 5-HT_7_ receptor. The agonist and antagonist were added simultaneously. Cell stimulation was performed for 1 h at room temperature. After incubation, cAMP measurements were performed with the homogeneous time-resolved fluorescence resonance energy transfer (TR-FRET) immunoassay using the LANCE Ultra cAMP kit (PerkinElmer). 10 μL of EucAMP Tracer Working Solution and 10 μL of ULight-anti-cAMP Tracer Working Solution were added, mixed, and incubated for 1 h. The TR-FRET signal was read on an EnVision microplate reader (PerkinElmer). IC50 and EC50 were determined by nonlinear regression analysis using GraphPad Prism 6.0 software.

### Functional Assay for the D_2_ Receptor

Test and reference compounds were dissolved in dimethyl sulfoxide (DMSO) at a concentration of 1 mM. Serial dilutions were prepared in 96-well microplate in assay buffer and 8 to 10 different concentrations of each compound were tested. A cellular aequorin-based functional assay was performed with recombinant CHO-K1 cells expressing mitochondrially targeted aequorin, human GPCR, and the promiscuous G protein Gαqi/5 for the D2 receptor. The assay was executed according to the previously described protocol.^1^ After thawing, the cells were transferred to assay buffer (DMEM/HAM’s F12 with 0.1% protease-free BSA) and centrifuged. The cell pellet was resuspended in assay buffer and coelenterazine h was added at a final concentration of 5 μM. The cell suspension was incubated at 16 °C, protected from light, with constant agitation for 16 h and then diluted with assay buffer to a concentration of 100 000 cells/mL. After 1 h of incubation, 50 μL of the cell suspension was dispensed using the automatic injectors built into the MicroBeta2 LumiJET radiometric and luminescence plate counter (PerkinElmer) into white opaque 96-well microplates preloaded with test compounds. Immediate light emission generated following calcium mobilization was recorded for 30 s. In the antagonist mode, after 30 min of incubation, the reference agonist was added to the above assay mix and light emission was recorded again. The final concentration of the reference agonist was equal to EC80 (30 nM apomorphine).

### Thermodynamic Solubility Assay

LC/MS grade methanol, LC/MS grade acetonitrile, Dulbecco’s phosphate-buffered saline (DPBS), and formic acid (>98%) were from Sigma-Aldrich. HPLC grade water was obtained from the HLP 5 apparatus (HYDROLAB Poland) and was filtered through a 0.2 μm filter before use. Each of the analyzed compounds (1.0 mg) was weighed in a volumetric flask using an analytical balance. The volume was brought to 1 mL with methanol to obtain a 1.0 mg mL^–1^ solution. A series of dilutions of the standard solutions of the investigated compounds were prepared by diluting 500 μL of the stock solutions with methanol to make 1 mL and, afterward, diluting 500 μL of the obtained solutions again with water to make 1 mL. The procedure was repeated several times to obtain solutions with the concentration of the compounds in the range of 15.6–1000 μg mL^–1^. Each of the resulting solutions was analyzed in triplicate using 1 μL injections, and the results were used to obtain calibration curves. Each of the analyzed compounds (1.0 mg) was weighed in a SEPARA vial (GVS, Italy) using an analytical balance. The volume was brought to 0.5 mL with DPBS and the mixture was constantly agitated at 20 °C for 24 h in a thermoshaker. Afterward, the mixture was filtered directly in the vial and the filtrate was analyzed. Each sample was injected in triplicate in two series: using 1 and 10 μL. For the determination of the thermodynamic solubility of the investigated compounds, the quantitative UPLC method was developed. Chromatograms were recorded using a Waters eλ PDA detector. Spectra were analyzed in the 200–700 nm range with 1.2 nm resolution and a sampling rate of 20 points s^–1^. For calibration and quantification, areas under the peaks of the investigated compounds on DAD chromatograms were used. All analytical data were processed using MassLynx V4.1 software (Waters Corporation, Milford, MA). Calibration and quantitative methods’ validation were made using Statistica v. 13. The thermodynamic solubility of the investigated compounds was calculated using the formula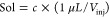 where Sol is the thermodynamic solubility in DPBS in 20 °C in μg mL^–1^, *c* is the concentration of the sample in μg mL^–1^ calculated using the obtained calibration curve, and *V*_inj_ is the volume of the injection in μL.

### Metabolic Stability Studies

The metabolic stability studies were performed by Eurofins Pharma Discovery Services, according to well-established procedures. Further methodological details are available on the company website (www.eurofinsdiscoveryservices.com) and the appropriate publications.

### Pharmacological Evaluation

#### Behavioral Studies

The experiments were performed on male Wistar rats (220–300 g) purchased from an accredited animal facility, the Jagiellonian University Medical College (Krakow, Poland). The animals were kept in a dark–light 12/12 h cycle (lights on at 8.00 a.m.), in groups of 4 per polycarbonate Makrolon type 3 cage (dimensions 26.5 × 15 × 42 cm^3^), at an ambient temperature of 21 ± 2 °C and relative humidity of 50–60%. Standard laboratory food (LSM-B) and filtered water was provided ad libitum. All of the experiments were carried out during the light phase, between 9.00 and 14.00. Each experimental group consisted of 6–8 rats/dose (with the exception of the social interaction test (SIT), where one group consisted of 7–8 pairs of rats). The animals were used only once and were killed immediately after experiments. All of the experimental procedures involving animals were carried out in full accordance with the ethical standards laid down by respective Polish and European (Directive no. 86/609/EEC) regulations and were approved by the I Local Ethics Commission at the Jagiellonian University Medical College in Krakow (Approval No 40/2018). The assays using reference drugs were performed at Jagiellonian University Medical College (Krakow, Poland) and the Institute of Psychiatry and Neurology, and the experimental procedures were approved by the IV Local Ethics Commission in Warsaw (Approval No 40/2008).

#### Drugs and Treatment

The following compounds were used: **7**, **11**, **16**, imipramine (hydrochloride, Adamed, Pienkow, Poland), escitalopram (hydrochloride; Adamed, Pienkow, Poland), diazepam (Adamed, Pienkow, Poland), quetiapine (Adamed, Pienkow, Poland), dizocilpine ((+)-MK-801, hydrogen maleate, Sigma-Aldrich, U.K.). Compounds **7**, **11**, and **16** and quetiapine were suspended in a 1.5% solution of Tween 80 (Sigma-Aldrich, U.K.), while diazepam, imipramine, escitalopram, and MK-801 were dissolved in saline immediately before administration and injected intraperitoneally (i.p.) at a volume of 2 mL/kg body mass. All of the compounds, with the exception of imipramine and MK-801, which were injected 15 min before the test, were administered 60 min before the tests. Control animals received a vehicle (1.5% Tween 80 or saline) according to the same injection regime.

#### Forced Swim Test (FST) in Rats

The test was carried out according to the previously described method of Porsolt et al.^82^ On the first day of the experiment, rats were individually placed in Plexiglas cylinders of height 40 cm and diameter 18 cm, filled with water up to 15 cm maintained at 25 °C. After a 15 min period, rats were removed from the water and dried under a 60 W light bulb. On the following day, rats were placed again in the cylinders and the total time of immobility was recorded throughout the 5 min test period. The water was changed after each animal.

#### MK-801-Induced Hyperlocomotor Activity Test in Rats

The experiment was performed in a darkened room using a Motor Monitor System (Campden Instruments, Ltd., U.K.) consisting of two Smart Frame Open Field stations (40 × 40 × 38 cm^3^) with 16 × 16 beams, located in a sound-attenuating chamber and connected to a PC software via a control chassis; 60 min after vehicle or compound administration and 15 min after MK-801 (0.2 mg/kg), the treated rats were individually placed in the center of the station. An automated Motor Monitor System recorded ambulation (in both the *X* and *Y* axes), the number of rearing and the total distance covered by each rat for 30 min, with data registered every 5 min.^65^

#### Vogel Conflict Drinking Test in Rats

The testing procedure, based on a method described by Vogel et al.,^83^ was performed using the Anxiety Monitoring System “Vogel test” produced by TSE Systems. The apparatus consisted of a polycarbonate cage (dimensions 26.5 × 15 × 42 cm^3^), equipped with a grid floor made from stainless steel bars and a drinking bottle containing tap water. Experimental chambers (two) were connected to PC software by a control chassis and a device that generates electric shocks. In this “conditional” model an electric shock is applied as a noxious stimulus. The testing procedure consisted of a 2-day habituation/adaptation period and an actual test. On the first day of the experiment, the rats were adapted to the test chamber for a 10 min adaptation period, during which they had free access to the water drinking bottle, followed by a 24 h water deprivation period. Afterward, they were allowed a 30 min free-drinking session in their home cages. This protocol of 24 h deprivation and adaptation period was repeated on the second day. On the third day, the animals were placed again in the test chamber 60 min after administration of the vehicle or the test compound and were given free access to the water drinking bottle for 5 min. Recording data started immediately after the first lick and after every 20 licks, rats were punished with an electric shock (0.5 mA, lasting 1 s). The impulses were released via the spout of the drinking bottle. The number of shocks received throughout a 5 min experimental session was recorded automatically and was used as an indication of anticonflict activity.

#### Hot Plate and Free-Drinking Tests in Rats

To exclude possible drug-induced changes in shock sensitivity or an increasing influence on thirst drive, which can lead to false-positive results in the Vogel conflict drinking test, stimulus threshold and water consumption during a free-drinking session were determined in separate groups of rats. In both of these two studies, the rats were manipulated similarly to the Vogel conflict drinking test, including two 24 h water deprivation periods separated by a 10 min adaptation session in experimental cages and 30 min of water availability in their home cages. In the free-drinking test, each animal was allowed to freely drink from the drinking bottle and the amount of water (g) consumed during 5 min was recorded for each rat. The pain threshold was evaluated using a hot plate test (Commat Ltd, Turkey) in rats. The plate was enclosed with a transparent Plexiglass cylinder (35 cm high) to keep the animal on the heated surface of the plate. The latency to pain reaction (lick a hind paw or jump) when the rat was placed on a hot plate (52.5 ± 0.5 °C, 19 cm diameter) was measured. The rat was removed from the plate immediately upon visible pain reaction or if no response occurred within 30 s.

#### Elevated Plus-Maze (EPM) Test in Rats

The testing procedure was based on a method described by Pellow and File.^84^ The Plus Maze apparatus (an automated device produced by Campden Instruments Ltd., United Kingdom) made of durable, high density, nonporous black plastic, elevated to a height of 50 cm, consisted of two open arms (50 × 10 cm^2^) and two closed arms (50 cm × 10 cm, and 30 cm high walls), arranged so that the two arms of each type were opposite each other. The floor of the Plus Maze was made of infrared transparent material which means that there are no visible sensors. The Plus Maze apparatus was connected to PC software via a control chassis. The experiments were conducted in a darkened room, with only the center of the maze illuminated with low-intensity light (30 lux measured on the maze level). Each rat was gently placed in the center of the Plus Maze, facing one of the closed arms, immediately after a 5 min adaptation period in a plastic black box (60 × 60 × 35 cm^3^), to increase the overall activity in the EPM. During a 5 min test period, the automated Motor Monitor System recorded the number of entries into the closed and open arms and the time spent in either type of arm. The device counted an effective arm-entry when the four paws of a rat were into any arm. The maze was thoroughly cleaned after each trial. The number of open-arms entries, total time spent in open arms, and the percentages of these parameters were used as indications of anxiolytic-like activity.

#### Exploratory Activity Measured in the EPM in Rats

To assess the influence of the tested compounds on the general exploratory activity of rats and control possible changes within, total ambulation (the total distance covered by a rat, and ambulation along both *X* and *Y* axes) and the total number of entries (into open and closed arms) were taken during a 5 min test period (i.e., the time equal to the observation period in the EPM test). The experiment was performed using EPM apparatus (details see above).

#### Social Interaction Test

Five days before the experiment, male Wistar rats were placed individually in home cages (dimensions 26.5 × 15 × 42 cm^3^) and exposed daily with the touch and presence of the experimenter. The social interaction test was conducted in opaque black boxes with dimensions of 60 × 60 × 60 cm^3^ in an experimental room dimly illuminated with the diffused light of 30–40 Lx. On the fifth day of social isolation, 24 h before the test, rats were individually placed in experimental cages for 10 min and allowed to freely explore the arena. After this time, the rats were transferred to their home cages. Each social interaction experiment involving two rats was carried out during the light phase of the light/dark cycle. The rats were selected from separate housing cages to make a pair for the study. The paired rats were matched for body weight within 15 g. The social interaction was measured 30 min after i.p. administration of MK-801 at a dose of 0.1 mg/kg, and 60 min after administration of the tested compound. Rats in pair received the same treatment. Each pair of rats was diagonally placed in opposite corners of the box facing away from each other. The behavior of the animals was measured over a 10 min period. The test box was wiped clean between each trial. Social interaction between two rats was expressed as the total time spent in social behavior, such as sniffing, genital investigation, chasing, and fighting with each other.

#### Novel Object Recognition Test (NORT) in Rats

The protocol was adapted from the original work of Ennaceur and Delacour.^85^ The experiment was conducted in opaque black boxes with dimensions of 60 × 60 × 60 cm^3^. The 2-day procedure consists of habituation to the test arena (without any objects) for 5 min on the first day and a test session comprising two 3 min trials separated by a 1 h intertrial interval on the second day. During the first trial (familiarization, T1), two identical objects (A1 and A2) were presented in opposite corners of the arena, approximately 10 cm from the walls. During the second trial (recognition, T2), one of the A objects was replaced by a novel object B so that the animals were presented with A (familiar) and B (novel) objects. Both trials lasted for 3 min and the animals were returned to their home cages after T1. Metal Coca-Cola cans and glass jars filled with sand were used as the objects. The heights of the objects were comparable (approximately 12 cm) and the objects were heavy enough so that the animals could not displace them. The sequence of presentations and the location of the objects were randomly assigned to each rat. The animals explored the objects by looking, licking, sniffing, or touching them while sniffing, but not when leaning against, standing, or sitting on the objects. Any rat exploring the two objects for <5 s within the 3 min duration of T1 or T2 was eliminated from the study. An experimenter, blind to the drug treatment, measured the exploration time of the objects. Based on the exploration time (*E*) of the two objects during T2, the discrimination index (DI) was calculated according to the formula: DI = (EB – EA)/(EA + EB). Using this metric, scores approaching zero reflect no preference, while positive values reflect a preference for the novel object and negative numbers reflect a preference for the familiar object. In the test variant, which used MK-801 to induce memory impairment, MK-801 was administered at a dose of 0.1 mg/kg (i.p.) 30 min before the familiarization phase (T1).

#### Open Field Test (OFT) in Rats

The compounds active in FST, SIT, and/or MK-801-induced hyperlocomotor activity test were tested for their impact on spontaneous locomotor activity at doses that were active in these previous studies. The experiment was performed as described above. Individual vehicle- or drug-injected animals were gently placed in the center of the station. An automated Motor Monitor System recorded ambulation (in *X* and *Y* axes), the number of rearing, and total distance covered by a rat for 30 min, with data registered every 5 min.

#### Statistical Analysis

All of the data are presented as the mean ± SEM. The statistical significance of the results was evaluated by a one-way ANOVA, followed by a Bonferroni’s Comparison Test.

#### Metabolic Safety

The experiments were carried out on female Wistar rats, with an initial body weight of 185–195 g. The animals were housed in pairs in plastic cages in constant temperature facilities exposed to a light–dark cycle; water and food were available ad libitum. Both control and experimental groups consisted of six animals. All experiments were conducted according to the guidelines of the Animal Use and Care Committee of the Jagiellonian University and were approved for realization (2018, Poland; Permissions No 126/2018). Statistical calculations were performed using GraphPad Prism 6 software. Results are given as arithmetic means with a standard error of the mean. Statistical significance was calculated using a two-way variance analysis (ANOVA) with repeated measures, followed by a Bonferroni post hoc test (daily changes in body weight and spontaneous activity) or a one-way variance analysis (ANOVA) followed by a Tukey post hoc test (biochemical analysis, differences were considered statistically significant at: *p ≤ 0.05, **p ≤ 0.01, ***p ≤ 0.001).

#### Palatable Western-Style Cafeteria Diet as a Reliable Method for Metabolic Syndrome Development

Female Wistar rats were housed in pairs. Four groups of 6 rats were fed on diets consisting of milk chocolate with nuts, cheese, salted peanuts, and 7% condensed milk with additional access to standard feed (Labofeed B, Morawski Manufacturer Feed, Poland) and water ad libitum, for 25 days. The palatable control group (palatable diet + vehicle) received vehicle (1% Tween 80, i.p.), while the palatable test groups (palatable diet + compound) received **11** or olanzapine injected intraperitoneally (i.p.) at a dose of 2 × 2 mg/kg b.w./day dissolved in 1% Tween 80. The body weights were measured daily immediately prior to administration of drugs.

On the 26th day, 20 min after i.p. administration of heparin (1000 units/rat) and thiopental (70 mg/kg b.w.), blood was collected from the animals.

The palatable diet contained: peanuts—100 g, 614 kcal; condensed milk—100 mL, 131 kcal; milk chocolate with hazelnuts—100 g, 195 kcal; cheese (Greek type)—100 g, 270 kcal. The standard diet contained 100 g of feed—280 kcal.

#### Biochemical Analysis

On the 25th day of the experiment, 20 min after intraperitoneal administration of heparin (5000 units/rat) and thiopental (70 mg/kg b.w.), blood was collected from the left carotid artery and then centrifuged at 600*g* (15 min, 4 °C) to obtain the plasma. The influence of the tested compounds on glucose, total cholesterol, HDL-cholesterol or triglyceride levels, and activity of alanine aminotransferase activity in plasma was analyzed. To determine the glucose, total cholesterol, HDL-cholesterol or triglyceride levels, and activity of alanine aminotransferase in plasma, standard enzymatic and spectrophotometric tests (Biomaxima S.A. Lublin, Poland) were used.

#### Determination of the Effect on Blood Pressure

On the 26th day, rats were anesthetized with thiopental (70 mg/kg) by i.p. injection. The left carotid artery was cannulated with tubing filled with heparin solution in saline to facilitate pressure measurements using Apparatus PowerLab 4/35 (ADInstruments, Australia). Blood pressure was measured: before intraperitoneal administration of AK37, olanzapine or 1% Tween 80—time 0 min (control pressure) and during the next 40 min.^75,86,87^

### Pharmacokinetics Study in Rats

#### Extraction Procedure

Plasma samples (150 μL) containing compounds **7**, **11**, and **16** were transferred to Eppendorf tubes and 10 μL of internal standard solution ((3-chloro-4-fluorophenyl)(4-fluoro-4-((((5-methylpyridin-2-yl)methyl)amino)methyl)piperidin-1-yl)methanone—50 ng/mL for compounds **7** and **11**, and 3-chloro-4-fluorophenyl)(4-fluoro-4-(((2-(2-(methylamino)phenoxy) ethyl)amino)methyl)piperidin-1-yl)methanone—500 ng/mL for **16** was added. The samples were alkalized with 20 μΛ of 4 M sodium hydroxide solution and after vortex-mixed were extracted with 1 mL of ethyl acetate/hexane (30:/, v/v) mixture for 20 min on a shaker (VXR Vibrax, IKA, Germany). Then, the samples were centrifuged (TDx Centrifuge, Abbott Laboratories), and the organic layers were transferred into new Eppendorf tubes containing 100 μL of methanol and 0.1 M sulfuric acid mixture (10:90, v/v). The samples were shaken and centrifuged again. Finally, 85–90 μL of each acidic layer was subjected to analysis via the HPLC system.

Frozen rat brains were thawed at room temperature, weighed, and then homogenized (4 mL/g) in distilled water with a tissue homogenizer TH220 (Omni International, Inc.). The tissue homogenates (2 mL) were transferred to glass tubes. Then, 10 μL of proper internal standard solution was added and samples were alkalized with 100 μL of 4 M sodium hydroxide solution. After vortex-mixing, samples were extracted with 6 mL of extraction mixture for 20 min on a shaker and centrifuged (Universal 32, Hettich, Germany). Subsequently, the organic layers were transferred into new glass tubes containing 150 μL of methanol and 0.1 M sulfuric acid mixture (10:90, v/v). Then, after shaking, the samples were centrifuged and 90 μL of each acidic layers was injected into the HPLC system.

#### Equipment and Chromatographic Conditions

The analysis of all tested compounds was performed in an HPLC system consisting of a P100 pump (Thermo Separation Products, San Jose, CA), an L-2200 autosampler, and an L-2420 UV/VIS detector (Merck-Hitachi). EZChrome Elite v. 3.2 software (Merck-Hitachi) was used for data acquisition. The chromatographic separation of all compounds and the internal standard was achieved at room temperature (22 ± 1 °C) on the Supelcosil PCN column 250 mm × 4.6 mm (Sigma-Aldrich, Germany) with 5 μm particles, protected with the Supelcosil LC-PCN guard column (Sigma-Aldrich, Germany) with the same packing material under isocratic conditions. The mobile phase consisted of 25 mM potassium dihydrogen phosphate buffer, pH 4.6: methanol: acetonitrile (51:40:9, v/v/v). The flow rate was 1.0 mL/min, and the detector wavelength was 203 nm. Validation of the chromatographic method was performed in accordance with the FDA guideline (Bioanalytical method validation, 2013). This included selectivity, linearity, accuracy and precision, limit of detection (LOD) and limit of quantitation (LOQ), recovery, and stability.

#### Pharmacokinetics Study

The compounds were administered via the intraperitoneal (i.p.) route to permit a direct comparison to the reference compounds, which have been previously studied following i.p. administration. Male Wistar rats weighing 250–300 g were housed in conditions of constant temperature with a 12:12 h light–dark cycle with access to food and water ad libitum. The animals were divided into two groups. In the first group (*n* = 3 for each compound), the animals were implanted with catheters (SAI Infusion Technologies) in the jugular vein under ketamine/xylazine anesthesia at least 2 days prior to the experiment. The animals (*n* = 3) received a single dose of 2 mg/kg (as a free base) of all tested compounds intraperitoneally and at 5, 15, 30, 60, 120, 180, and 300 min after dosing, blood samples were collected from the cannulated jugular vein into heparinized tubes. Plasma was separated by centrifugation at 3000 rpm for 10 min and stored at −80 °C until analysis. In the second group (*n* = 9 for each compound) animals (without catheters) were exsanguinated 30, 60, and 180 min post-dose (*n* = 3 per time point). Plasma and brains were harvested and stored at −80 °C until analysis. All animal procedures were approved by the 2^nd^ Local Institutional Animal Care and Use Committee in Krakow.

#### Pharmacokinetics Analysis

Plasma concentration versus time data was analyzed by a noncompartmental approach (NCA) in Phoenix WinNonlin v. 7.0 (Pharsight Corporation, a Certara Company, Princeton, NJ). The maximum concentration (*C*_max_) and the time to reach peak concentration (*t*_max_) in plasma following intraperitoneal dosing were obtained directly from the concentration versus time data. The terminal elimination rate constant (λ_z_) was assessed by linear regression and terminal half-life (*t*_0.5λz_) was calculated as ln 2/λ_z_. The area under the concentration–time curve from the time of dosing to infinity (AUC_0–∞_) was calculated by the linear trapezoidal rule. The extrapolated terminal area was defined as *C_n_*/λ_z_, where *C_n_* is the last data point. Clearance (CL/*F*) was calculated as *D*/AUC_0–∞_, the volume of distribution based on the terminal phase (*V*_z_/*F*) was estimated as *D*/(λ_z_·AUC_0–∞_), where *F* is the fraction absorbed, and mean residence time (MRT) as calculated as AUC_0–∞_/AUMC_0–∞_, where AMUC_0–∞_ is the area under the first moment curve from the time of dosing to infinity. Data were presented as mean ± SD.

## Supporting Information Available

In silico predictions of biotransformation routes, molecular druglike properties, and the detailed characteristics of binding modes in the target receptors; detailed procedures for the preparation of the intermediates **I**–**VI**; HPLC traces, ^1^H NMR, and ^13^C NMR spectra of the final products **7**, **11**, and **16**; and the effect of **11** and olanzapine on body weight (PDF)
5-HT_2A_R homology model (PDB)
5-HT_6_R homology model (PDB)
5-HT_7_R homology model (PDB)
D2R homology model (PDB)
Molecular Formula Strings (CSV)

## Supplementary Material

jm1c00497_si_001.pdf

jm1c00497_si_002.pdb

jm1c00497_si_003.pdb

jm1c00497_si_004.pdb

jm1c00497_si_005.pdb

## Abbreviations Used

BPSD: behavioral and psychological symptoms of dementia
CNS MPO: Central Nervous System Multiparameter Optimization
EPS: elevated plus maze test
FST: forced swimming test (Porsolt test)
SIT: social interaction test
NORT: novel object recognition task
MK-801: dizocilpine
SB214111: 4-bromo-*N*-[4-methoxy-3-(4-methylpiperazin-1-yl)phenyl]benzenesulfonamide
SB258719: 3-methyl-*N*-[(1*R*)-1-methyl-3-(4-methyl-1-piperidinyl)propyl]-*N*-methylbenzenesulfonamide hydrochloride
PAINS: pan assay interference compounds
