# Supplementary material for: Multifunctional Arylsulfone and Arylsulfonamide-Based Ligands with Prominent Mood-Modulating Activity and Benign Safety Profile, Targeting Neuropsychiatric Symptoms of Dementia
Source: J Med Chem. 2021 Aug 26;64(17):12603–29. doi: 10.1021/acs.jmedchem.1c00497 (PMC8436213; doi:10.1021/acs.jmedchem.1c00497)
Supplement: Supplementary file 1 — jm1c00497_si_001.pdf [file jm1c00497_si_001.pdf]

Supporting Information

Multifunctional arylsulfone and arylsulfonamide-based ligands with prominent mood-modulating activity and benign safety profile, targeting neuropsychiatric symptoms of dementia

Monika Marcinkowska [a], Adam Bucki [a], Joanna Sniecikowska [a], Agnieszka Zagórska [a], Nikola Fajkis-Zajączkowska [a], Agata Siwek [a], Monika Gluch-Lutwin [a], Paweł Żmudzki [a], Magdalena Jastrzebska-Wiesek [a], Anna Partyka [a], Anna Wesołowska [a], Michał Abram[a], Katarzyna Przejczowska-Pomierny [a], Agnieszka Cios [a], Elżbieta Wyska [a], Kamil Mika [a], Magdalena Kotańska [a], Paweł Mierzejewski [b], Marcin Kolaczkowski [a,c]*

[a] Jagiellonian University Medical College, Faculty of Pharmacy, 9 Medyczna St., 30-688 Krakow, Poland

[b] Institute of Psychiatry and Neurology, 9 Sobieskiego Street, 02-957 Warsaw, Poland

[c] Adamed Pharma S.A., 6A Mariana Adamkiewicza Street, Pienkow, 05-152 Czosnow, Poland

*Corresponding Author Information:
Phone: (+48)126205460
E-mail: adam.bucki@uj.edu.pl

Contents **Page number**

1. Metabolic pathways prediction. 3

    Figure S1. Biotransformation prediction for compounds 1 (A) and 9 (B) - ADMET Predictor.... 3

2. Drug-like properties. 4

    Table S1. Molecular properties of compounds 2-22. 4

3. The binding mode characteristics in the target receptors. 5

    Figure S2. The proposed binding mode of compound 7 and 16 in the 5-HT2A receptor 5

    Figure S3. The proposed binding mode of compound 7 and 16 in the 5-HT6 receptor............ 6

    Figure S4. The proposed binding mode of compound 7 and 16 in the 5-HT7 receptor............ 7

    Figure S5. The proposed binding mode of compound 7 and 16 in the D2 receptor................. 8

4. Detailed procedures for preparation of the intermediates I-VI 9

    a) General procedure for the synthesis of intermediates I a-k. 9

    b) General procedure for the synthesis of sulfone derivatives II a-k... 12

    c) General procedure for the synthesis of amine intermediates III a-k 16

    d) Synthesis of (3S,5S)-tert-(1-(3-(6-fluorobenzo[d]isoxazol-3-yl)propyl)-5-methylpyrrolidin-3-yl)carbamate (V) 18

    e) Synthesis of (3S,5S)-1-(3-(6-fluorobenzo[d]isoxazol-3-yl)propyl)-5-methylpyrrolidin-3-amine hydrochloride (VI)... 19

5. HPLC traces and 1H and 13C NMR spectra of compounds 7, 11 and 16 20

    a) Characterizations of compound 7 20

    Figure S6. 1H NMR (300 MHz, CDCl3) spectrum of compound 7.. 20

    Figure S7. ^{13}C NMR (75 MHz, $CDCl_3$) spectrum of compound 7. 20

    Figure S8. HPLC spectrum of compound 7 (99.45%). 21

    b) Characterizations of compound 11 22

    Figure S9. ^{1}H NMR (300 MHz, $CDCl_3$) spectrum of compound 11. 22

    Figure S10. ^{13}C NMR (75 MHz, $CDCl_3$) spectrum of compound 11. 22

    Figure S11. HPLC spectrum of compound 11 (100.00%). 23

    c) Characterizations of compound 16 24

    Figure S12. ^{1}H NMR (300 MHz, $CDCl_3$) spectrum of compound 16. 24

    Figure S13. ^{13}C NMR (75 MHz, $CDCl_3$) spectrum of compound 16. 24

    Figure S14. HPLC spectrum of compound 16 (96.87%). 25

6. Results of in vivo studies (compound 11)...26

    Figure S15. Effect of 11 and olanzapine on body weight 26

1. Metabolic pathways prediction.

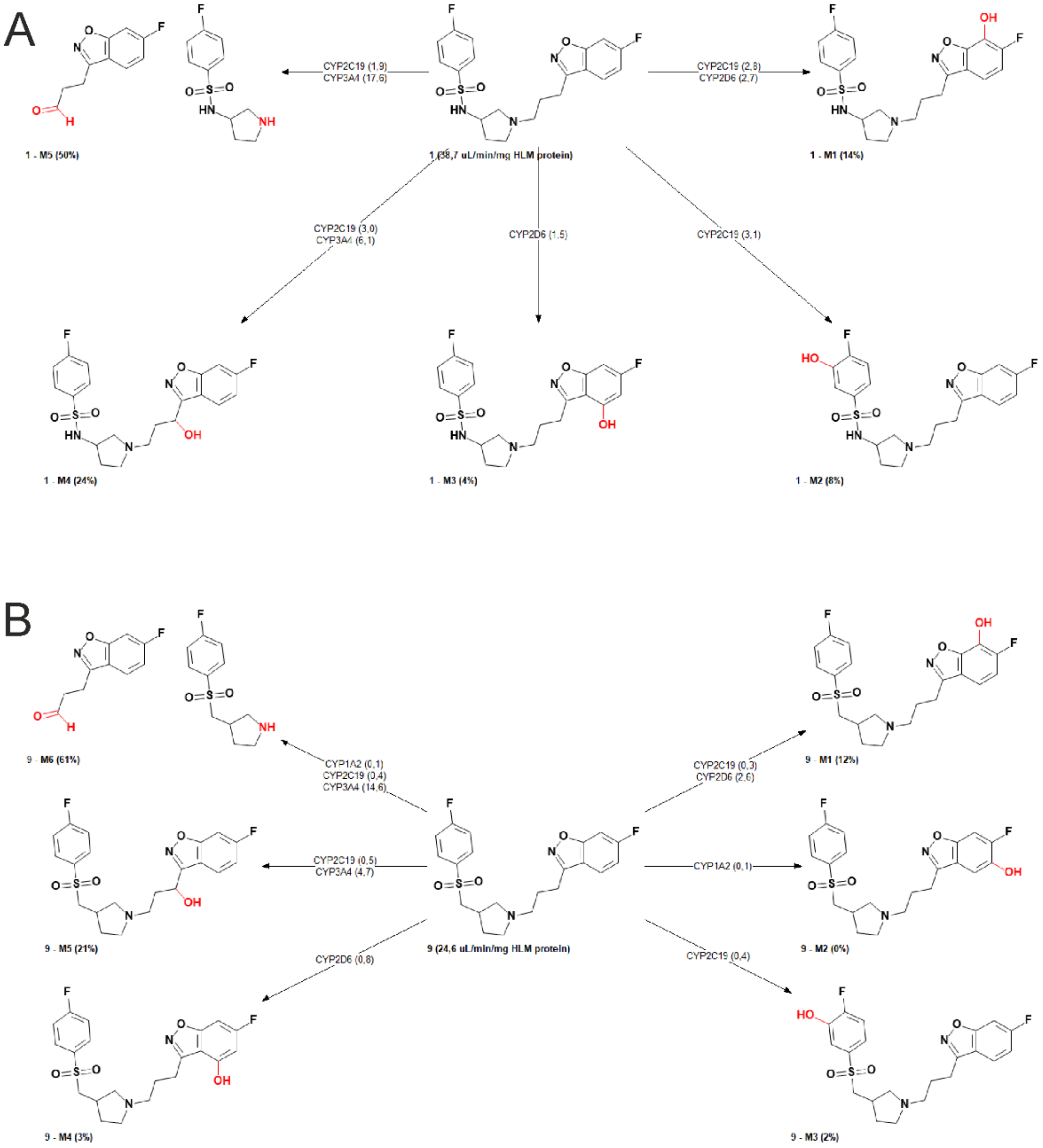

Figure S1. Biotransformation prediction for compounds 1 (A) and 9 (B) - ADMET Predictor. CYP3A4-dependent N-dealkylation appears to be the main route of metabolism of compound 1 (17.6 µL/min/mg, 50% of metabolites) and to a lower extent compound 9 (14.6 µL/min/mg, 61 % of metabolites). The remaining metabolic activity of a variety of CYP isoforms is focused on hydroxylation reactions in the area of the aliphatic chain (24% and 21% of metabolites, respectively) and the aromatic rings (26% - 17% of metabolites, respectively). The predicted total value of CYP-mediated clearance was 38.7 µL/min/mg HLM protein (compound **1**) and 24.6 µL/min/mg HLM protein (compound **9**), according to ADMET Predictor.

2. Drug-like properties.

Table S1. Molecular properties of compounds 2-22.

Compound	pK_a	LogD$_{7.4}$	Lipinski's rule of 5				Veber filter		CNS
			LogP	MW	HBD	HBA	RB	TPSA	MPO
**2**	7.4	3.6	3.9	452.5	0	4	7	63.4	4.1
**3**	7.4	3.6	3.9	430.5	0	4	7	63.4	4.2
**4**	7.3	2.9	3.2	438.5	0	4	7	63.4	4.9
**5**	7.3	3.8	4.1	471.4	0	4	7	63.4	3.8
**6**	7.3	3.4	3.7	454.9	0	4	7	63.4	4.3
**7**	7.4	3.5	3.8	470.5	0	4	8	63.4	4.1
**8**	7.3	3.5	3.8	470.5	0	4	8	63.4	4.1
**9**	7.4	2.8	3.1	420.5	0	4	7	63.4	5.1
**10**	7.3	2.8	3.1	420.5	0	4	7	63.4	5.1
**11**	7.3	3.2	3.5	436.9	0	4	7	63.4	4.6
**12**	7.4	3.1	3.4	416.5	0	4	7	63.4	4.8
**13**	7.8	3.1	3.5	451.9	1	4	6	75.4	4.4
**14**	7.7	3.3	3.6	469.9	1	4	6	75.4	4.1
**15**	7.8	3.4	3.7	473.6	1	4	6	75.4	4.0
**16**	7.4	3.8	3.9	473.6	1	4	6	75.4	3.7
**17**	7.4	2.8	2.9	457.5	1	4	6	88.6	4.7
**18**	7.4	3.9	4.1	491.6	1	4	6	75.4	3.4
**19**	7.4	4.4	4.5	508.0	1	4	6	75.4	3.1
**20**	7.5	4.9	5.1	522.1	1	4	6	75.4	2.8
**21**	7.8	3.5	4.0	467.6	1	4	6	75.4	3.8
**22**	7.8	4.1	4.6	502.0	1	4	6	75.4	3.0

Properties calculated using InstantJChem software (ChemAxon): pKa – negative log of the strongest basic dissociation constant; LogD$_{7.4}$ – Predicted distribution coefficient at pH = 7.4; LogP - Predicted octanol/water partition coefficient; MW – molecular weight; HBD – hydrogen bond donor count; HBA – hydrogen bond acceptor count; RB – rotatable bonds; TPSA – total polar surface area. CNS MPO – Multi-Parameter Optimization for Central Nervous System-active drugs (value 0–6).

3. The binding mode characteristics in the target receptors.

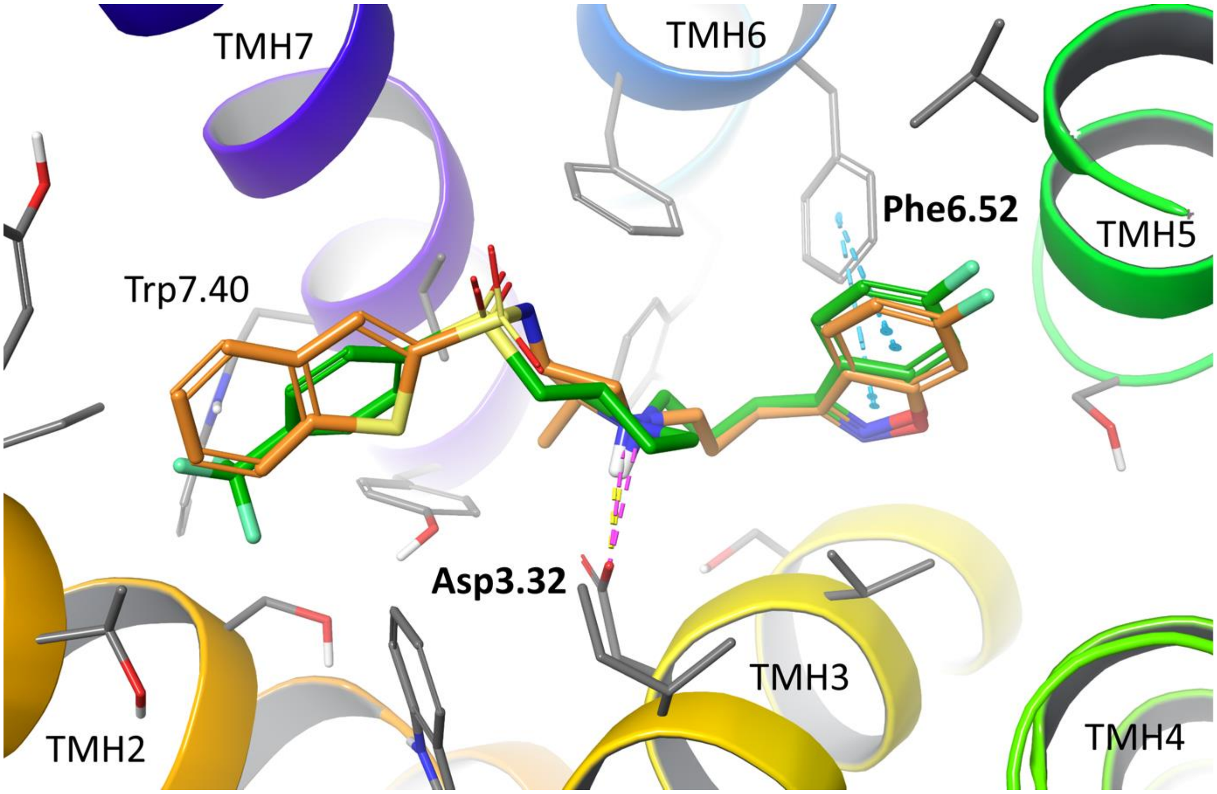

Figure S2. The proposed binding mode of compounds 7 and 16 in the 5-HT2A receptor homology model based on 4IB4. The (methyl)pyrrolidin-1-yl-propyl moiety interacts with Asp3.32 (salt bridge/charge-reinforced hydrogen bond) and the 6-fluoro-1,2-benzoxazole with Phe6.52 (π-π stacking) in the orthosteric binding site. The 1-sulfonyl-4-(trifluoromethyl)benzene moiety of compound **7** (green) and 1-benzothiophene-2-sulfonamide moiety of compound **16** (orange) form non-specific interactions in the additional binding cavity. Amino acid residues engaged in ligand binding (within 4 Å from the ligand atoms) are represented as thick sticks.

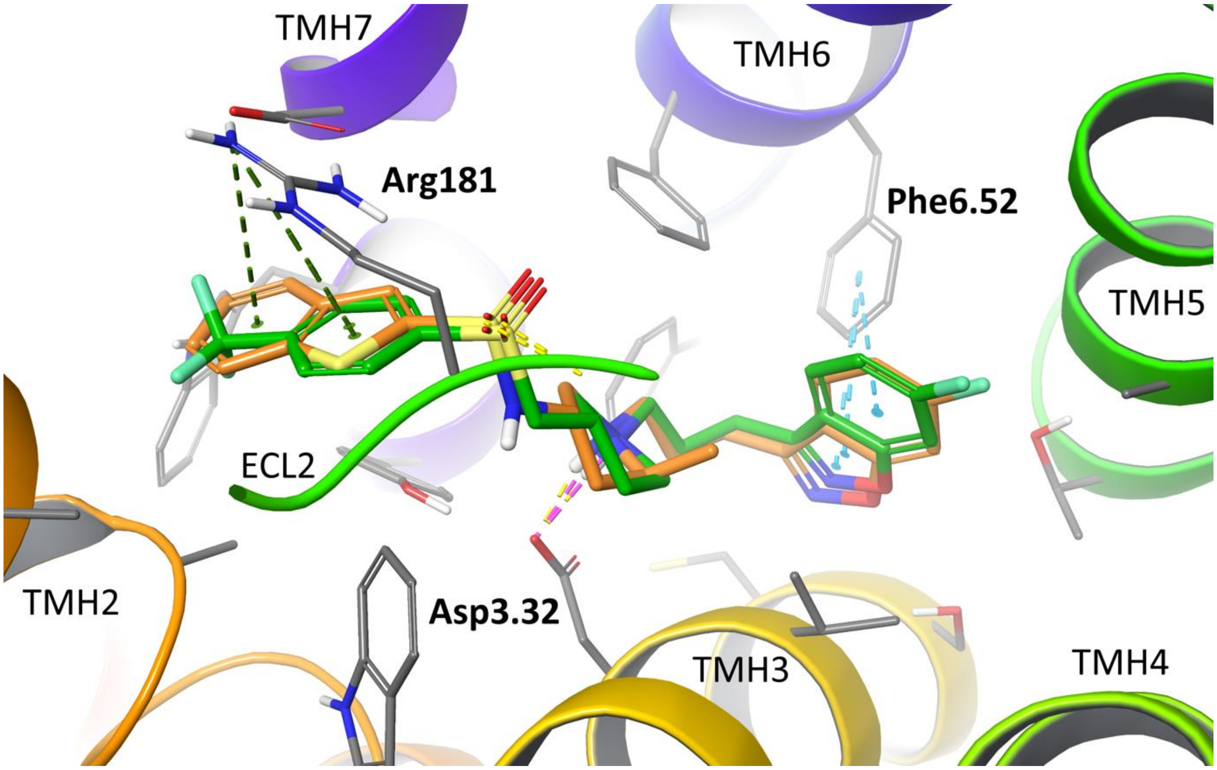

Figure S3. The proposed binding mode of compounds 7 and 16 in the 5-HT6 receptor homology model based on 4IAR. The (methyl)pyrrolidin-1-yl-propyl moiety interacts with Asp3.32 (salt bridge/charge-reinforced hydrogen bond) and the 6-fluoro-1,2-benzoxazole with Phe6.52 (π-π stacking) in the orthosteric binding site. Whereas the 1-sulfonyl-4-(trifluoromethyl)benzene moiety of compound **7** (green) and 1-benzothiophene-2-sulfonamide moiety of compound **16** (orange) interact with Arg181 (cation-π) in the additional binding cavity. Amino acid residues engaged in ligand binding (within 4 Å from the ligand atoms) are represented as thick sticks.

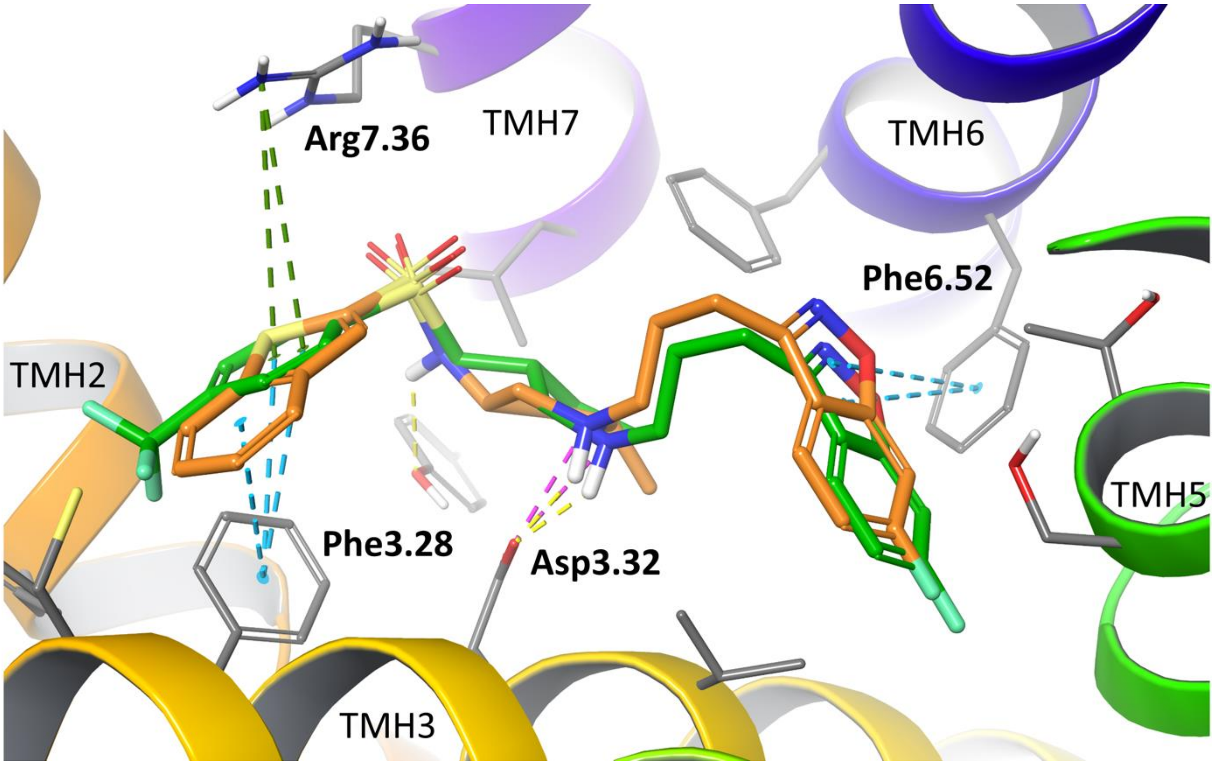

Figure S4. The proposed binding mode of compounds 7 and 16 in the 5-HT7 receptor homology model based on 2RH1. The (methyl)pyrrolidin-1-yl-propyl moiety interacts with Asp3.32 (salt bridge/charge-reinforced hydrogen bond) and the 6-fluoro-1,2-benzoxazole with Phe6.52 (π-π stacking) in the orthosteric binding site. Whereas the 1-sulfonyl-4-(trifluoromethyl)benzene moiety of compound **7** (green) and 1-benzothiophene-2-sulfonamide moiety of compound **16** (orange) interacts with Phe3.28 (CH-π stacking) and with Arg7.36 (cation-π) in the additional binding cavity. Amino acid residues engaged in ligand binding (within 4 Å from the ligand atoms) are represented as thick sticks.

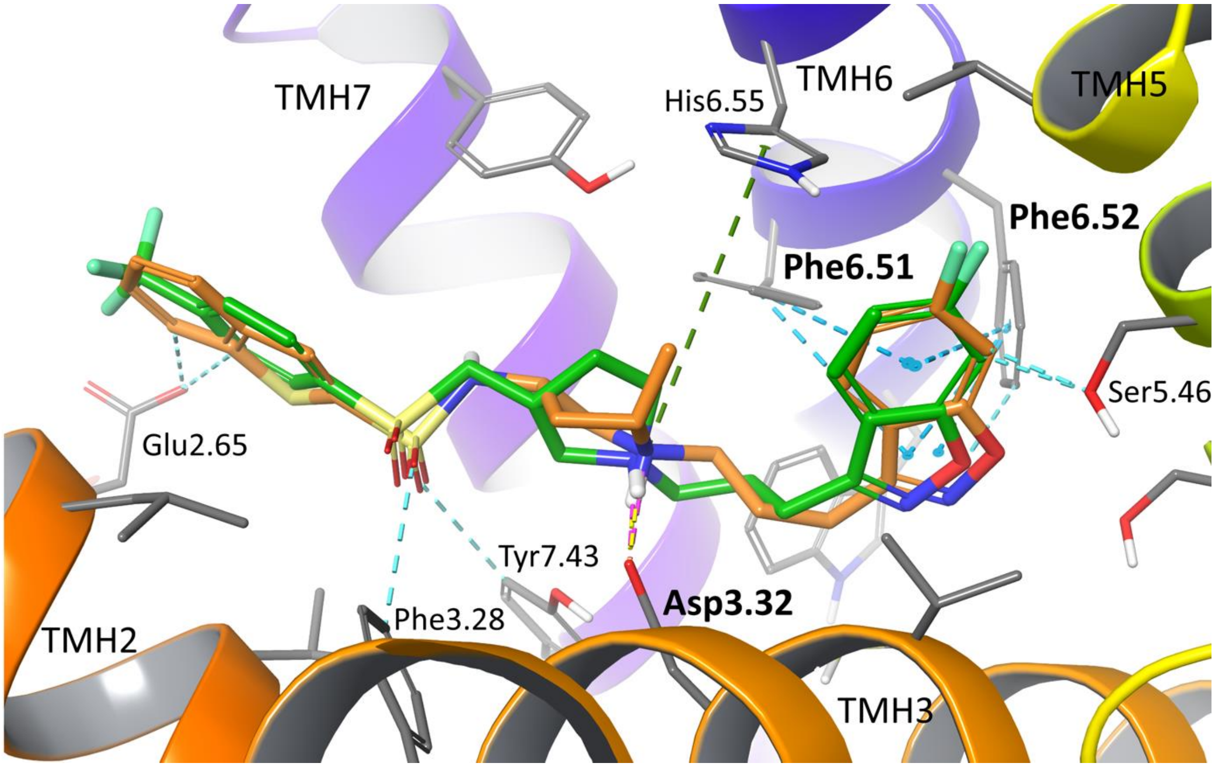

Figure S5. The proposed binding mode of compounds 7 and 16 in the D2 receptor homology model based on 3PBL. The (methyl)pyrrolidin-1-yl-propyl moiety interacts with Asp3.32 (salt bridge/charge-reinforced hydrogen bond) and the 6-fluoro-1,2-benzoxazole with Phe6.51/6.52 (π-π stacking) in the orthosteric binding site. The 1-sulfonyl-4-(trifluoromethyl)benzene moiety of compound **7** (green) and 1-benzothiophene-2-sulfonamide moiety of compound **16** (orange) form weaker aromatic H-bonds in the additional binding cavity. Amino acid residues engaged in ligand binding (within 4 Å from the ligand atoms) are represented as thick sticks.

4. Detailed procedures for preparation of the intermediates I-VI

a) General procedure for the synthesis of intermediates I a-k.

A mixture of appropriate thiol derivative (1 equiv) anhydrous potassium carbonate (1 equiv) in 5mL of absolute ethanol was stirred under the reflux for 10 min. Next the *N*-Boc-3-(chloromethyl) pyrrolidine (1 equiv) was added and the resulting mixture was stirred for 2-6 hours at 80 °C. After this time, the solvent was evaporated, 20 ml of water was added and extracted with dichloromethane (20 ml). The collected organic phases were dried over anhydrous sodium sulfate, filtered, and the solvent was removed in vacuo. The reaction mixture was purified by column chromatography using n-hexane/ethyl acetate 85:15 (v/v) as eluent.

*tert-butyl 3-[(naphthalen-2-ylsulfanyl)methyl]pyrrolidine-1-carboxylate (**Ia**)*

The title compound was prepared starting from naphthalene-2-thiol (1.4 mmol, 1 equiv, 0.224 g), tert-butyl-3-(chloromethyl)pyrrolidine-1-carboxylate (1.4 mmol, 1 equiv, 0.308 g) and potassium carbonate (1.4 mmol, 1 equiv, 0.193 g). Yield: 81 %, yellowish oil. ^{1}H NMR (300 MHz, $CDCl_3$, δ): 7.83–7.70 (m, 4H), 7.54–7.39 (m, 3H), 3.58–3.46 (m, 2H), 3.37–3.22 (m, 1H), 3.16–3.01 (m, 3H), 2.53–2.38 (m, 1H), 2.14–2.00 (m, 1H), 1.78–1.64 (m, 1H), 1.44 (s, 9H). Formula: $C_{20}H_{25}NO_2S$

*tert-butyl 3-{[(3,4-dimethylphenyl)sulfanyl]methyl}pyrrolidine-1-carboxylate (**Ib**)*

The title compound was prepared starting from 3,4-dimethylbenzene-1-thiol (1.4 mmol, 1 equiv, 0.194 g), tert-butyl-3-(chloromethyl)pyrrolidine-1-carboxylate (1.4 mmol, 1 equiv, 0.308 g) and potassium carbonate (1.4 mmol, 1 equiv, 0.193 g). Yield: 84 %, colorless oil. ^{1}H NMR (300 MHz, $CDCl_3$) δ: 7.36–7.24 (m, 2H), 7.23-–7.12 (d, *J* = 7.6 Hz, 1H), 3.51 (br dd, *J* = 4.7, 6.4 Hz, 2H), 3.36–3.25 (m, 1H), 3.15–3.05 (m, 1H), 2.99–2.92 (m, 2H), 2.43 (br dd, *J* = 6.7, 14.4 Hz, 1H), 2.32 (s, 6H), 2.15–2.03 (m, 1H), 1.75–1.62 (m, 1H), 1.45 (s, 9H). Formula: $C_{18}H_{27}NO_2S$

*tert-butyl 3-{[(3,4-difluorophenyl)sulfanyl]methyl}pyrrolidine-1-carboxylate (**Ic**)*

The title compound was prepared starting from 3,4-difluorobenzene-1-thiol (1.4 mmol, 1 equiv, 0.205 g), tert-butyl-3-(chloromethyl)pyrrolidine-1-carboxylate (1.4 mmol, 1 equiv, 0.308 g) and potassium carbonate (1.4 mmol, 1 equiv, 0.193 g). Yield: 87%, colorless oil. ^{1}H NMR (300 MHz, $CDCl_3$, δ): 7.17 (ddd, *J* = 1.8, 7.3, 10.8 Hz, 1H), 7.12–7.03 (m, 2H), 3.57–3.49 (m, 1H), 3.48–3.40 (m, 1H), 3.38–3.21 (m, 1H), 3.16–2.99 (m, 1H), 2.91 (dd, *J* = 2.6, 7.3 Hz, 2H), 2.36 (quin, *J* = 7.3, 14.8 Hz, 1H), 2.11–1.98 (m, 1H), 1.71–1.61 (m, 1H), 1.45 (s, 9H). Formula: $C_{16}H_{21}F_2NO_2S$

tert-butyl 3-{[(3,4-dichlorophenyl)sulfanyl]methyl}pyrrolidine-1-carboxylate (***Id***)

The title compound was prepared starting from 3,4-dichlorobenzene-1-thiol (1.4 mmol, 1 equiv, 0.251 g), tert-butyl-3-(chloromethyl)pyrrolidine-1-carboxylate (1.4 mmol, 1 equiv, 0.308 g) and potassium carbonate (1.4 mmol, 1 equiv, 0.193 g). Yield: 91%, yellowish oil. ^{1}H NMR (300 MHz, $CDCl_3$, δ): 7.40 (d, *J* = 2.3 Hz, 1H), 7.37–7.30 (m, 1H), 7.15 (dd, *J* = 2.3, 8.2 Hz, 1H), 3.61–3.44 (m, 2H), 3.38–3.23 (m, 1H), 3.07 (br dd, *J* = 7.9, 10.3 Hz, 1H), 2.98–2.87 (m, 2H), 2.39 (td, *J* = 7.2, 14.9 Hz, 1H), 2.13–1.97 (m, 1H), 1.76–1.59 (m, 1H), 1.45 (s, 9H). Formula: $C_{16}H_{21}Cl_2NO_2S$

*tert-butyl 3-{[(3-chloro-4-fluorophenyl)sulfanyl]methyl}pyrrolidine-1-carboxylate (**Ie**)*

The title compound was prepared starting from 3-chloro-4-fluorobenzene-1-thiol (1.4 mmol, 1 equiv, 0.228 g), tert-butyl-3-(chloromethyl)pyrrolidine-1-carboxylate (1.4 mmol, 1 equiv, 0.308 g) and potassium carbonate (1.4 mmol, 1 equiv, 0.193 g). Yield: 68 %, yellowish oil. ^{1}H NMR (300 MHz, $CDCl_3$, δ): 7.45–7.35 (m, 1H), 7.28–7.19 (m, 1H), 7.12–7.00 (m, 1H), 3.65–3.39 (m, 2H), 3.38–3.21 (m, 1H), 3.15–2.98 (m, 1H), 2.91 (dd, *J* = 2.9, 7.6 Hz, 2H), 2.45–2.27 (m, 1H), 2.13–1.96 (m, 1H), 1.75–1.56 (m, 1H), 1.45 (s, 9H). Formula: $C_{16}H_{21}ClFNO_2S$

*tert-butyl-3-({[4-(trifluoromethyl)phenyl]sulfanyl}methyl)pyrrolidine-1-carboxylate (**If**)*

The title compound was prepared starting from 4-(trifluoromethyl)benzene-1-thiol (1.4 mmol, 1 equiv, 0.249 g), tert-butyl-3-(chloromethyl)pyrrolidine-1-carboxylate (1.4 mmol, 1 equiv, 0.308 g) and potassium carbonate (1.4 mmol, 1 equiv, 0.193 g). Yield: 84%, whitish solid. ^{1}H NMR (300

MHz, $CDCl_3$, δ):7.52 (d, *J* = 8.2 Hz, 2H), 7.37 (d, *J* = 8.2 Hz, 2H), 3.61–3.43 (m, 2H), 3.37–3.22 (m, 1H), 3.17–3.04 (m, 1H), 3.00 (d, *J* = 7.0 Hz, 2H), 2.44 (td, *J* = 7.5, 14.8 Hz, 1H), 2.15–1.98 (m, 1H), 1.78–1.64 (m, 1H), 1.45 (s, 9H). Formula: $C_{17}H_{22}F_3NO_2S$

*tert-butyl-3-({[3-(trifluoromethyl)phenyl]sulfanyl}methyl)pyrrolidine-1-carboxylate (**Ig**)*

The title compound was prepared starting from 3-(trifluoromethyl)benzene-1-thiol (1.4 mmol, 1 equiv, 0.249 g), tert-butyl-3-(chloromethyl)pyrrolidine-1-carboxylate (1.4 mmol, 1 equiv, 0.308 g) and potassium carbonate (1.4 mmol, 1 equiv, 0.193 g). Yield: 74 %, yellowish oil. ^{1}H NMR (300 MHz, $CDCl_3$, δ): 7.55 (s, 1H), 7.52–7.35 (m, 3H), 3.58–3.45 (m, 2H), 3.37–3.24 (m, 1H), 3.15–3.05 (m, 1H), 3.00 (br dd, *J* = 4.4, 6.7 Hz, 2H), 2.48–2.35 (m, 1H), 2.13–2.00 (m, 1H), 1.76–1.62 (m, 1H), 1.45 (s, 9H). Formula: $C_{17}H_{22}F_3NO_2S$

*tert-butyl 3-{[(4-fluorophenyl)sulfanyl]methyl}pyrrolidine-1-carboxylate (**Ih**)*

The title compound was prepared starting from 4-fluorobenzene-1-thiol (1.4 mmol, 1 equiv, 0.179 g), tert-butyl-3-(chloromethyl)pyrrolidine-1-carboxylate (1.4 mmol, 1 equiv, 0.308 g) and potassium carbonate (1.4 mmol, 1 equiv, 0.193 g). Yield: 90 %, yellowish oil. ^{1}H NMR (300 MHz, $CDCl_3$, δ): 7.40–7.31 (m, 2H), 6.99 (t, *J* = 8.5 Hz, 2H), 3.51 (br dd, *J* = 4.4, 6.7 Hz, 2H), 3.34–3.22 (m, 1H), 3.14–2.99 (m, 1H), 2.88 (br dd, *J* = 4.1, 7.0 Hz, 2H), 2.42–2.28 (m, 1H), 2.08–1.98 (m, 1H), 1.66 (br s, 1H), 1.44 (s, 9H). Formula: $C_{16}H_{22}FNO_2S$

*tert-butyl 3-{[(3-fluorophenyl)sulfanyl]methyl}pyrrolidine-1-carboxylate (**Ii**)*

The title compound was prepared starting from 3-fluorobenzene-1-thiol (1.4 mmol, 1 equiv, 0.179 g), tert-butyl-3-(chloromethyl)pyrrolidine-1-carboxylate (1.4 mmol, 1 equiv, 0.308 g) and potassium carbonate (1.4 mmol, 1 equiv, 0.193 g). Yield: 87 %, yellowish oil. ^{1}H NMR (300 MHz, $CDCl_3$, δ): 7.27–7.19 (m, 1H), 7.11–7.06 (m, 1H), 7.02 (td, *J* = 2.1, 9.4 Hz, 1H), 6.87 (dt, *J* = 2.1, 8.4 Hz, 1H), 3.56–3.47 (m, 2H), 3.37–3.25 (m, 1H), 3.14–3.04 (m, 1H), 2.99–2.91 (m, 2H), 2.50–2.34 (m, 1H), 2.13–2.04 (m, 1H), 1.71 (br d, *J* = 8.2 Hz, 1H), 1.45 (s, 9H). Formula: $C_{16}H_{22}FNO_2S$

*tert-butyl 3-{[(3-chlorophenyl)sulfanyl]methyl}pyrrolidine-1-carboxylate (**Ij**)*

The title compound was prepared starting from 3-chlorobenzene-1-thiol (1.4 mmol, 1 equiv, 0.202 g), tert-butyl-3-(chloromethyl)pyrrolidine-1-carboxylate (1.4 mmol, 1 equiv, 0.308 g) and potassium carbonate (1.4 mmol, 1 equiv, 0.193 g). Yield: 81 %, yellowish oil. ^{1}H NMR (300 MHz, $CDCl_3$, δ): 7.32–7.28 (m, 1H), 7.23-–7.12 (m, 3H), 3.51 (br dd, *J* = 4.7, 6.4 Hz, 2H), 3.36–3.25 (m, 1H), 3.13–3.01 (m, 1H), 2.99–2.91 (m, 2H), 2.40 (br dd, *J* = 6.7, 14.4 Hz, 1H), 2.13–2.05 (m, 1H), 1.74–1.64 (m, 1H), 1.45 (s, 9H); Formula: $C_{16}H_{22}ClNO_2S$

*tert-butyl 3-{[(3-methylphenyl)sulfanyl]methyl}pyrrolidine-1-carboxylate (**Ik**)*

The title compound was prepared starting from 3-methylbenzene-1-thiol (1.4 mmol, 1 equiv, 0.174 g), tert-butyl-3-(chloromethyl)pyrrolidine-1-carboxylate (1.4 mmol, 1 equiv, 0.308 g) and potassium carbonate (1.4 mmol, 1 equiv, 0.193 g). Yield: 69 %, colorless oil. ^{1}H NMR (300 MHz, $CDCl_3$) δ: 7.31–7.22 (m, 1H), 7.232–7.10 (m, 3H), 3.50 (br dd, *J* = 4.7, 6.4 Hz, 2H), 3.33–3.22 (m, 1H), 3.12–3.04 (m, 1H), 2.97–2.93 (m, 2H), 2.47 (s, 3H), 2.42 (br dd, *J* = 6.7, 14.4 Hz, 1H), 2.11–2.05 (m, 1H), 1.73–1.66 (m, 1H), 1.45 (s, 9H). Formula: $C_{17}H_{25}NO_2S$

b) General procedure for the synthesis of sulfone derivatives II a-k

A mixture of appropriate intermediate **Ia-h** (1 equiv) and meta-chloroperoxybenzoic acid (3 equiv) in 5 ml of anhydrous dichloromethane was stirred for 1 hour at room temperature. Next, the reaction mixture was heated to 35 °C and stirred for 12 hours. After that time, the solvent was evaporated, 20 ml of saturated $NaHCO_3$ was added and extracted with dichloromethane (20 ml). The organic layer was separated from the aqueous phase, then dried over anhydrous sodium sulfate, filtered and the solvent was evaporated under reduced pressure. The reaction mixture was purified by flash column chromatography over silica gel using n-hexane/ethyl acetate 60:40 (v/v) as eluent.

*tert-butyl 3-[(naphthalene-2-sulfonyl)methyl]pyrrolidine-1-carboxylate (**IIa**)*

The title compound was prepared starting from *tert-butyl 3-[(naphthalen-2-ylsulfanyl)methyl] pyrrolidine-1-carboxylate* **Ia** (0.9 mmol, 1 equiv, 0.308 g) and meta-chloroperbenzoic acid (2.7 mmol, 3 equiv, 0.466 g). Yield: 91 %, whitish solid. ^{1}H NMR (300 MHz, $CDCl_3$, δ): 8.51 (s, 1H), 8.08–7.98 (m, 2H), 7.94 (br d, *J* = 7.6 Hz, 1H), 7.87 (dd, *J* = 1.8, 8.8 Hz, 1H), 7.74–7.59 (m, 2H), 3.71–3.56 (m, 1H), 3.52–3.40 (m, 1H), 3.33–3.17 (m, 3H), 3.06–2.93 (m, 1H), 2.74–2.59 (m, 1H), 2.27–2.01 (m, 1H), 1.83–1.58 (m, 1H), 1.41 (br s, 9H). LC-MS (ESI) calcd for $C_{20}H_{25}NO_4S$ 375.48 [M + H^+], found 376 [M + H^+].

*tert-butyl 3-[(3,4-dimethylbenzenesulfonyl)methyl]pyrrolidine-1-carboxylate (**IIb**)*

The title compound was prepared starting from tert-butyl 3-{[(3,4-dimethylphenyl) sulfanyl]methyl}pyrrolidine-1-carboxylate **Ib** (0.9 mmol, 1 equiv, 0.289 g) and meta-chloroperbenzoic acid (2.7 mmol, 3 equiv, 0.466 g). Yield: 65 %, whitish solid. ^{1}H NMR (300 MHz, $CDCl_3$, δ): 7.69–7.59 (m, 2H), 7.32 (d, *J* = 7.6 Hz, 1H), 3.61 (dd, *J* = 7.0, 11.1 Hz, 1H), 3.43 (br s, 1H), 3.32–3.04 (m, 3H), 2.97 (br dd, *J* = 8.5, 10.8 Hz, 1H), 2.70–2.54 (m, 1H), 2.35 (s, 6H), 2.24–2.03 (m, 1H), 1.78–1.58 (m, 1H), 1.43 (s, 9H). LC-MS (ESI) calcd for $C_{18}H_{27}NO_4S$ 353.47 [M + H^+], found 354 [M + H^+].

*tert-butyl 3-[(3,4-difluorobenzenesulfonyl)methyl]pyrrolidine-1-carboxylate (**IIc**)*

The title compound was prepared starting from *tert-butyl 3-{[(3,4-difluorophenyl)sulfanyl] methyl}pyrrolidine-1-carboxylate* **Ic** (0.9 mmol, 1 equiv, 0.296 g) and meta-chloroperbenzoic acid (2.7 mmol, 3 equiv, 0.466 g). Yield: 85 %, whitish solid. ^{1}H NMR (300 MHz, $CDCl_3$, δ): 7.86–7.63 (m, 2H), 7.39 (dt, *J* = 7.3, 8.9 Hz, 1H), 3.64 (br dd, *J* = 7.9, 10.8 Hz, 1H), 3.55–3.41 (m, 1H), 3.35–3.22 (m, 1H), 3.17 (br t, *J* = 6.2 Hz, 2H), 3.06–2.91 (m, 1H), 2.77–2.49 (m, 1H), 2.29–2.06 (m, 1H), 1.79–1.64 (m, 1H), 1.44 (s, 9H) . LC-MS (ESI) calcd for $C_{16}H_{21}F_2NO_4S$ 361.40 [M + H^+], found 362 [M + H^+].

*tert-butyl 3-[(3,4-dichlorobenzenesulfonyl)methyl]pyrrolidine-1-carboxylate (**IId**)*

The title compound was prepared starting from *tert-butyl 3-{[(3,4-dichlorophenyl)sulfanyl] methyl}pyrrolidine-1-carboxylate* **Id** (0.9 mmol, 1 equiv, 0.326 g) and meta-chloroperbenzoic acid (2.7 mmol, 3 equiv, 0.466 g). Yield: 65 %, whitish solid. ^{1}H NMR (300 MHz, $CDCl_3$, δ): 8.01 (d, *J* = 2.3 Hz, 1H), 7.79–7.72 (m, 1H), 7.71–7.63 (m, 1H), 3.64 (br t, *J* = 8.2 Hz, 1H), 3.46 (br s, 1H), 3.35–3.09 (m, 3H), 3.01 (dd, *J* = 8.2, 11.1 Hz, 1H), 2.65 (br s, 1H), 2.17 (br s, 1H), 1.70 (br s, 1H), 1.44 (s, 9H). LC-MS (ESI) calcd for $C_{16}H_{21}Cl_2NO_4S$ 394.31 [M + H^+], found 395 [M + H^+].

*tert-butyl 3-[(3-chloro-4-fluorobenzenesulfonyl)methyl]pyrrolidine-1-carboxylate (**IIe**)*

The title compound was prepared starting from *tert-butyl 3-{[(3-chloro-4-fluorophenyl)sulfanyl]methyl}pyrrolidine-1-carboxylate* **Ie** (0.9 mmol, 1 equiv, 0.311 g) and meta-chloroperbenzoic acid (2.7 mmol, 3 equiv, 0.466 g). Yield: 63 %, whitish solid. ^{1}H NMR (300 MHz, $CDCl_3$, δ): 8.01 (dd, *J* = 2.1, 6.7 Hz, 1H), 7.83 (ddd, *J* = 2.1, 4.5, 8.6 Hz, 1H), 7.35 (t, *J* = 8.5 Hz, 1H), 3.64 (br dd, *J* = 7.6, 10.5 Hz, 1H), 3.52–3.37 (m, 1H), 3.35–3.09 (m, 3H), 3.01 (dd, *J* = 8.2, 11.1 Hz, 1H), 2.76–2.56 (m, 1H), 2.16 (br s, 1H), 1.69 (br s, 1H), 1.44 (s, 9H). LC-MS (ESI) calcd for $C_{16}H_{21}ClFNO_4S$ 377.86 [M + H^+], found 378 [M + H^+].

*tert-butyl 3-{[4-(trifluoromethyl)benzenesulfonyl]methyl}pyrrolidine-1-carboxylate (**IIf**)*

The title compound was prepared starting from *tert-butyl-3-({[4-(trifluoromethyl) phenyl]sulfanyl}methyl)pyrrolidine-1-carboxylate* **If** (0.9 mmol, 1 equiv, 0.325 g) and meta-chloroperbenzoic acid (2.7 mmol, 3 equiv, 0.466 g). Yield: 76 %, whitish solid.^{1}H NMR (300 MHz, $CDCl_3$, δ): 8.08 (d, *J* = 8.2 Hz, 2H), 7.87 (d, *J* = 8.8 Hz, 2H), 3.72–3.57 (m, 1H), 3.53–3.38 (m, 1H), 3.35–3.10 (m, 3H), 3.02 (dd, *J* = 8.2, 11.1 Hz, 1H), 2.66 (br s, 1H), 2.17 (s, 1H), 1.65 (br s, 1H), 1.44 (s, 9H). LC-MS (ESI) calcd for $C_{17}H_{22}F_3NO_4S$ 393.42 [M + H^+], found 394 [M + H^+].

*tert-butyl 3-{[3-(trifluoromethyl)benzenesulfonyl]methyl}pyrrolidine-1-carboxylate (**IIg**)*

The title compound was prepared starting from *tert-butyl-3-({[3-(trifluoromethyl) phenyl]sulfanyl}methyl)pyrrolidine-1-carboxylate* **Ig** (0.9 mmol, 1 equiv, 0.325 g) and meta-chloroperbenzoic acid (2.7 mmol, 3 equiv, 0.466 g). Yield: 69 %, yellowish oil. ^{1}H NMR (300 MHz, $CDCl_3$, δ): 8.20 (s, 1H), 8.13 (d, *J* = 7.6 Hz, 1H), 7.95 (br d, *J* = 8.2 Hz, 1H), 7.81–7.72 (m, 1H), 3.65 (dd, *J* = 7.3, 10.8 Hz, 1H), 3.54–3.44 (m, 1H), 3.37–3.24 (m, 1H), 3.23–3.14 (m, 2H), 3.02 (dd, *J* = 8.2, 11.1 Hz, 1H), 2.79–2.58 (m, 1H), 2.27–2.09 (m, 1H), 1.80–1.63 (m, 1H), 1.44 (s, 9H). LC-MS (ESI) calcd for $C_{17}H_{22}F_3NO_4S$ 393.42 [M + H^+], found 394 [M + H^+].

*tert-butyl 3-[(4-fluorobenzenesulfonyl)methyl]pyrrolidine-1-carboxylate (**IIh**)*

The title compound was prepared starting from *tert-butyl 3-{[(4-fluorophenyl)sulfanyl] methyl}pyrrolidine-1-carboxylate* **Ih** (0.9 mmol, 1 equiv, 0.280 g) and meta-chloroperbenzoic acid (2.7 mmol, 3 equiv, 0.466 g). Yield: 55 %, whitish solid. ^{1}H NMR (300 MHz, $CDCl_3$, δ): 7.94 (dd, *J* = 5.3, 8.8 Hz, 2H), 7.32–7.20 (m, 2H), 3.62 (br dd, *J* = 7.6, 10.5 Hz, 1H), 3.55–3.40 (m, 1H), 3.34–3.20 (m, 1H), 3.16 (br t, *J* = 5.9 Hz, 2H), 2.98 (br t, *J* = 9.4 Hz, 1H), 2.72–2.54 (m, 1H), 2.25–2.03 (m, 1H), 1.79–1.58 (m, 1H), 1.43 (s, 9H). LC-MS (ESI) calcd for $C_{16}H_{22}FNO_4S$ 343.41 [M + H^+], found 344 [M + H^+].

*tert-butyl-3-[(3-fluorobenzenesulfonyl)methyl]pyrrolidine-1-carboxylate (**IIi**)*

The title compound was prepared starting from tert-butyl 3-{[(3-fluorophenyl)sulfanyl] methyl}pyrrolidine-1-carboxylate **Ii** (0.9 mmol, 1 equiv, 0.280 g) and meta-chloroperbenzoic acid (2.7 mmol, 3 equiv, 0.466 g). Yield: 67 %, whitish solid. ^{1}H NMR (300 MHz, $CDCl_3$, δ): 7.76–7.69 (m, 1H), 7.68–7.53 (m, 2H), 7.38 (dt, *J* = 2.1, 8.4 Hz, 1H), 3.63 (dd, *J* = 7.6, 11.1 Hz, 1H), 3.45 (br d, *J* = 7.0 Hz, 1H), 3.33–3.10 (m, 3H), 2.99 (br t, *J* = 9.4 Hz, 1H), 2.65 (br s, 1H), 2.16 (br s, 1H), 1.73 (br s, 1H), 1.44 (s, 9H). LC-MS (ESI) calcd for $C_{16}H_{22}FNO_4S$ 343.41 [M + H^+], found 344 [M + H^+].

*tert-butyl-3-[(3-chlorobenzenesulfonyl)methyl]pyrrolidine-1-carboxylate (**IIj**)*

The title compound was prepared starting from tert-butyl 3-{[(3-chlorophenyl)sulfanyl] methyl}pyrrolidine-1-carboxylate **Ij** (0.9 mmol, 1 equiv, 0.295 g) and meta-chloroperbenzoic acid (2.7 mmol, 3 equiv, 0.466 g). Yield: 70 %, whitish solid. ^{1}H NMR (300 MHz, $CDCl_3$, δ): 7.92 (t, *J* = 1.8 Hz, 1H), 7.86–7.79 (m, 1H), 7.70–7.62 (m, 1H), 7.59–7.50 (m, 1H), 3.59–3.40 (m, 2H), 3.38–3.22 (m, 1H), 3.17 (br d, *J* = 6.4 Hz, 2H), 3.00 (dd, *J* = 8.2, 11.1 Hz, 1H), 2.72–2.52 (m, 1H), 2.26–2.10 (m, 1H), 1.77–1.64 (m, 1H), 1.44 (s, 9H). LC-MS (ESI) calcd for $C_{16}H_{22}ClNO_4S$ 359.87 [M + H^+], found 360 [M + H^+].

*tert-butyl 3-[(3-methylbenzenesulfonyl)methyl]pyrrolidine-1-carboxylate (**IIk**)*

The title compound was prepared starting from *tert-butyl 3-{[(3- methylphenyl) sulfanyl]methyl}pyrrolidine-1-carboxylate* **Ik** (0.9 mmol, 1 equiv, 0.277 g) and meta-chloroperbenzoic acid (2.7 mmol, 3 equiv, 0.466 g). Yield: 50 %, whitish solid. ^{1}H NMR (300 MHz, $CDCl_3$, δ): 7.76–7.67 (m, 2H), 7.50–7.44 (m, 2H), 3.61 (dd, *J* = 7.3, 10.8 Hz, 1H), 3.44 (br s, 1H), 3.32–3.07 (m, 3H), 3.04–2.90 (m, 1H), 2.71–2.57 (m, 1H), 2.46 (s, 3H), 2.13 (br s, 1H), 1.65 (br s, 1H), 1.44 (s, 9H). LC-MS (ESI) calcd for $C_{17}H_{25}NO_4S$ 339.45 [M + H^+], found 340 [M + H^+].

c) General procedure for the synthesis of amine intermediates III a-k

A solution of appropriate intermediate **IIa-k** (0.7 mmol) and 1M hydrochloric acid in ethyl acetate (15 mL) was stirred at room temperature for 12 hours. After this time, the solution was filtered and the resulting solid was allowed to dry for 24 h under the vacuo.

*3-((naphthalen-1-ylsulfonyl)methyl)pyrrolidine hydrochloride (**IIIa**)*

Yield 35%, white solid. ^{1}H NMR (300 MHz, CD_3OD, δ): 8.58 (d, *J* = 1.2 Hz, 1H), 8.18–8.08 (m, 2H), 8.07–8.01 (m, 1H), 7.93 (dd, *J* = 2.1, 8.5 Hz, 1H), 7.79–7.62 (m, 2H), 3.67–3.45 (m, 3H),

3.44–3.33 (m, 1H), 3.27–3.05 (m, 2H), 2.85–2.66 (m, 1H), 2.36–2.22 (m, 1H), 1.81 (qd, *J* = 9.2, 13.5 Hz, 1H) N*H* protons not detected

*3-(((3,4-dimethylphenyl)sulfonyl)methyl)pyrrolidine hydrochloride (**IIIb**)*

Yield 60%, white solid. ^{1}H NMR (300 MHz, CD_3OD, δ): 7.73–7.62 (m, 2H), 7.42 (d, *J* = 7.6 Hz, 1H), 3.62–3.33 (m, 4H), 3.24 (s, 1H), 3.08 (dd, *J* = 9.4, 11.7 Hz, 1H), 2.79–2.62 (m, 1H), 2.38 (s, 6H), 2.33–2.20 (m, 1H), 1.86–1.71 (m, 1H), N*H* protons not detected

*3-(((3,4-difluorophenyl)sulfonyl)methyl)pyrrolidine hydrochloride (**IIIc**)*

Yield 21%, white solid. ^{1}H NMR (300 MHz, CD_3OD, δ): 7.94 (ddd, *J* = 2.3, 7.5, 9.5 Hz, 1H), 7.88–7.78 (m, 1H), 7.66–7.54 (m, 1H), 3.67–3.47 (m, 3H), 3.46–3.33 (m, 1H), 3.29–3.16 (m, 1H), 3.11 (dd, *J* = 9.4, 11.7 Hz, 1H), 2.85–2.65 (m, 1H), 2.38–2.23 (m, 1H), 1.81 (qd, *J* = 9.2, 12.9 Hz, 1H), N*H* protons not detected

*3-(((3,4-dichlorophenyl)sulfonyl)methyl)pyrrolidine hydrochloride (**IIId**)*

Yield 86%, white solid. ^{1}H NMR (300 MHz, CD_3OD, δ): 8.16–8.09 (m, 1H), 7.93–7.81 (m, 2H), 3.67–3.35 (m, 4H), 3.27–3.17 (m, 1H), 3.11 (dd, *J* = 9.4, 11.7 Hz, 1H), 2.85–2.66 (m, 1H), 2.39–2.23 (m, 1H), 1.90–1.72 (m, 1H), N*H* protons not detected

*3-(((3-chloro-4-fluorophenyl)sulfonyl)methyl)pyrrolidine hydrochloride (**IIIe**)*

Yield 78%, white solid. ^{1}H NMR (300 MHz, CD_3OD, δ):8.13 (dd, *J* = 2.3, 6.4 Hz, 1H), 8.01–7.92 (m, 1H), 7.56 (t, *J* = 8.8 Hz, 1H), 3.65–3.35 (m, 4H), 3.28–3.17 (m, 1H), 3.11 (dd, *J* = 9.4, 11.7 Hz, 1H), 2.85–2.67 (m, 1H), 2.39–2.23 (m, 1H), 1.90–1.73 (m, 1H), N*H* protons not detected

*3-(((4-(trifluoromethyl)phenyl)sulfonyl)methyl)pyrrolidine hydrochloride (**IIIf**)*

Yield 50%, white solid. ^{1}H NMR (300 MHz, CD_3OD, δ): 8.18 (d, *J* = 8.2 Hz, 2H), 8.00 (d, *J* = 8.2 Hz, 2H), 3.69–3.45 (m, 3H), 3.45–3.35 (m, 1H), 3.29–3.17 (m, 1H), 3.11 (dd, *J* = 9.4, 11.7 Hz, 1H), 2.85–2.66 (m, 1H), 2.39–2.22 (m, 1H), 1.82 (qd, *J* = 9.2, 12.9 Hz, 1H), N*H* protons not detected

*3-(((3-(trifluoromethyl)phenyl)sulfonyl)methyl)pyrrolidine hydrochloride (**IIIg**)*

Yield 37%, white solid. ^{1}H NMR (300 MHz, CD_3OD, δ): 8.30–8.21 (m, 2H), 8.09 (d, *J* = 7.6 Hz, 1H), 7.96–7.86 (m, 1H), 3.68–3.48 (m, 3H), 3.45–3.34 (m, 1H), 3.28–3.07 (m, 2H), 2.88–2.70 (m, 1H), 2.40–2.24 (m, 1H), 1.83 (qd, *J* = 9.2, 13.5 Hz, 1H), N*H* protons not detected

*3-(((4-fluorophenyl)sulfonyl)methyl)pyrrolidine hydrochloride (**IIIh**)*

Yield 47%, white solid. ^{1}H NMR (300 MHz, CD_3OD, δ): 8.08–7.96 (m, 2H), 7.47–7.33 (m, 2H), 3.65–3.33 (m, 4H), 3.28–3.16 (m, 1H), 3.09 (dd, *J* = 9.4, 11.7 Hz, 1H), 2.85–2.65 (m, 1H), 2.38–2.21 (m, 1H), 1.80 (qd, *J* = 9.2, 13.5 Hz, 1H), N*H* protons not detected

*3-(((3-fluorophenyl)sulfonyl)methyl)pyrrolidine hydrochloride (**IIIi**)*

Yield 62%, white solid. ^{1}H NMR (300 MHz, CD_3OD, δ): 7.84–7.78 (m, 1H), 7.76–7.66 (m, 2H), 7.58–7.48 (m, 1H), 3.66–3.35 (m, 4H), 3.29–3.17 (m, 1H), 3.11 (dd, *J* = 9.1, 12.0 Hz, 1H), 2.83–2.66 (m, 1H), 2.37–2.23 (m, 1H), 1.81 (qd, *J* = 9.3, 13.3 Hz, 1H), N*H* protons not detected

*3-(((3-chlorophenyl)sulfonyl)methyl)pyrrolidine hydrochloride (**IIIj**)*

Yield 41%, white solid. ^{1}H NMR (300 MHz, CD_3OD, δ): 7.98 (t, *J* = 1.8 Hz, 1H), 7.94–7.87 (m, 1H), 7.82–7.75 (m, 1H), 7.71–7.62 (m, 1H), 3.66–3.33 (m, 4H), 3.28–3.17 (m, 1H), 3.10 (dd, *J* = 9.4, 11.7 Hz, 1H), 2.84–2.67 (m, 1H), 2.37–2.24 (m, 1H), 1.89–1.73 (m, 1H), N*H* protons not detected

*3-((m-tolylsulfonyl)methyl)pyrrolidine hydrochloride (**IIIk**)*

Yield 78%, white solid. ^{1}H NMR (300 MHz, CD_3OD, δ): 7.81–7.71 (m, 2H), 7.62–7.49 (m, 2H), 3.64–3.33 (m, 4H), 3.28–3.16 (m, 1H), 3.14–3.03 (m, 1H), 2.81–2.63 (m, 1H), 2.47 (s, 3H), 2.35–2.22 (m, 1H), 1.80 (qd, *J* = 9.2, 13.5 Hz, 1H), N*H* protons not detected

d) Synthesis of (3S,5S)-tert-(1-(3-(6-fluorobenzo[d]isoxazol-3-yl)propyl)-5-methylpyrrolidin-3-yl)carbamate (V)

A mixture of 3-(3-chloropropyl)-6-fluorobenzo[d]isoxazole (10.0 mmol, 1.0 equiv, 2.13 g), (*3S, 5S*) *tert*-butyl 5-methyl-pyrrolidin-3-yl-carbamate (11.0 mmol, 1.1 equiv, 2.2 g), potassium carbonate (3.0 equiv) and catalytic amount of potassium iodide in acetonitrile (60 mL) was stirred at 60 °C for 48 h. After that time, reaction mixture was cooled to the room temperature, the solid

was filtrated and the solvent was evaporated under the reduced pressure. Next, the crude product was purified by column chromatography over silica gel using dichloromethane/methanol 90:10 (v/v) as eluent. Yield 48%, yellowish oil, ^{1}H NMR (300 MHz, $CDCl_3$, δ): 7.58 (dd, *J* = 5.0, 8.8 Hz, 1H), 7.25 (dd, *J* = 2.1, 8.5 Hz, 1H), 7.01 (dt, *J* = 2.2, 8.8 Hz, 1H), 3.83–3.76 (m, 1H), 3.31–3.26 (m, 1H), 2.97–2.86 (m, 2H), 2.91 (br s, 1H), 2.13 (dd, *J* = 9.67, 7.33 Hz, 1H), 2.07–2.02 (m, 2H), 1.95–1.85 (m, 2H), 1.76-1.56 (m, 2H), 1.24–1.17 (m, 1H), 1.39 (s, 9H), 0.92 (d, J = 6.45 Hz, 3H); Formula $C_{20}H_{28}FN_3O_4$; MS (ESI^+): m/z 378 $[M+H]^+$

e) Synthesis of (3S,5S)-1-(3-(6-fluorobenzo[d]isoxazol-3-yl)propyl)-5-methylpyrrolidin-3-amine hydrochloride (VI)

(3S,5S)-tert-(1-(3-(6-fluorobenzo[d]isoxazol-3-yl)propyl)-5-methylpyrrolidin-3-yl)carbamate **V** (4.26 mmol, 1.0 equiv, 1.60 g) was mixed with 1.0 M solution of hydrochloric acid in ethyl acetate (80 ml), and stirred for 18 h (until the disappearance of starting materials - TLC monitoring). Then ethyl acetate was removed under reduced pressure to obtain dark oil, which was washed with ethyl acetate and dried under the vacuum and the product was used in the next step without further purification. Yield 91%, brown oil, ^{1}H NMR (300 MHz, CD_3OD, δ): 7.79 (dd, *J* = 5.0, 8.8 Hz, 1H), 7.38 (dd, *J* = 2.1, 8.5 Hz, 1H), 7.12 (dt, *J* = 2.2, 8.8 Hz, 1H), 3.91–3.82 (m, 1H), 3.41–3.31 (m, 1H), 3.02–2.88 (m, 2H), 2.27 (dd, *J* = 9.67, 7.33 Hz, 1H), 2.15–2.10 (m, 2H), 1.99–1.89 (m, 2H), 1.82-1.67 (m, 2H), 1.32–1.23 (m, 1H), 1.42 (s, 9H), NH protons not detected; LC-MS (ESI) calcd for $C_{14}H_{19}ClFN_3O$ 299.77 [M + H^+], found 278 [M + H^+].

5. HPLC traces and 1H and 13C NMR spectra of compounds 7, 11 and 16

a) Characterizations of compound 7

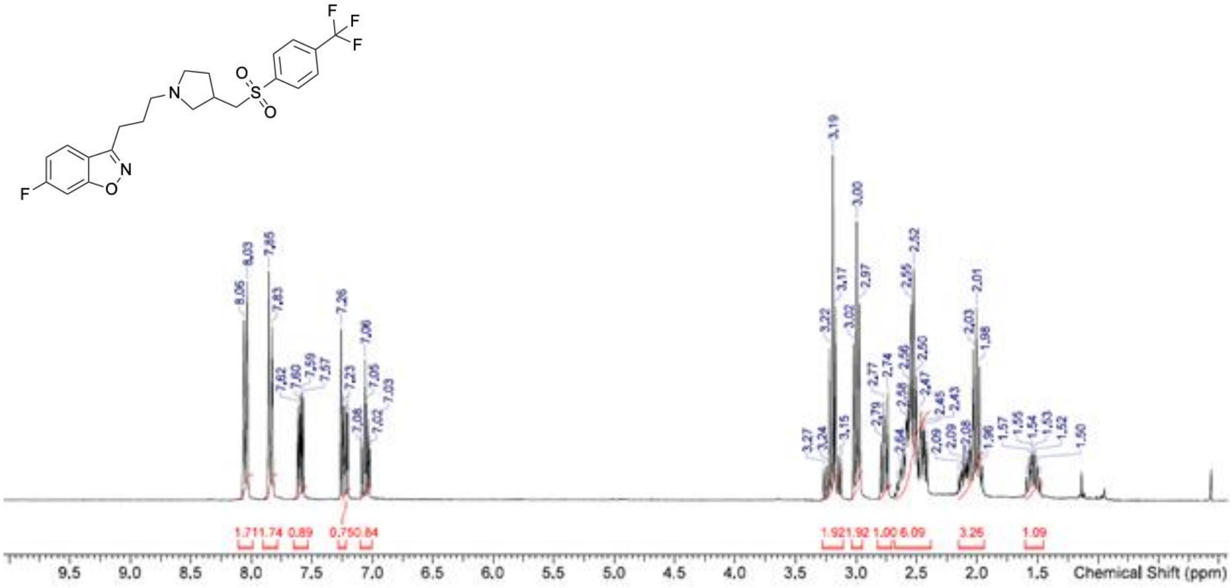

Figure S6. 1H NMR (300 MHz, CDCl3) spectrum of compound 7.

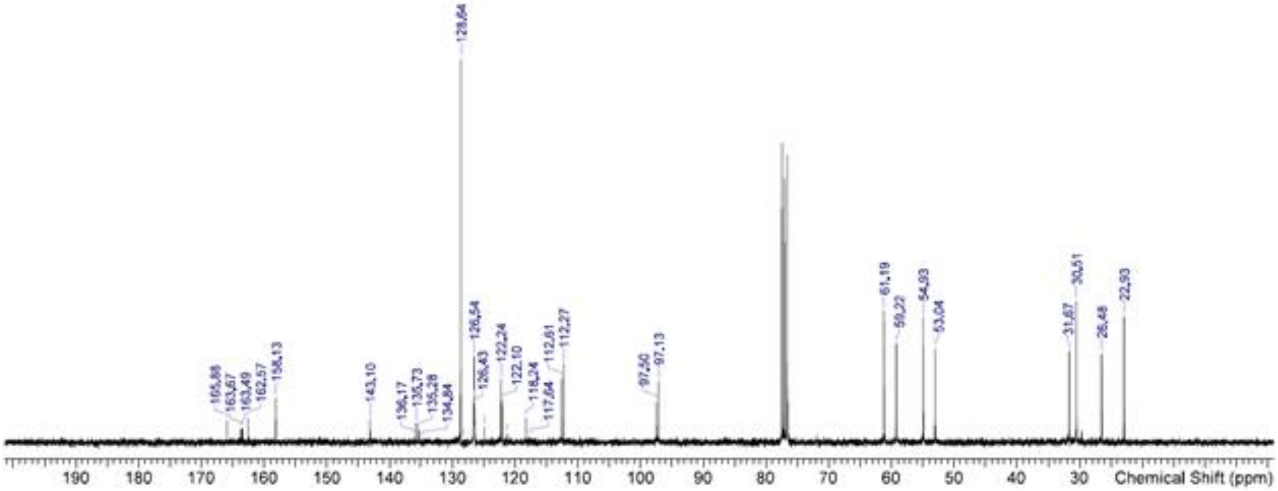

Figure S7. ^{13}C NMR (75 MHz, $CDCl_3$) spectrum of compound 7.

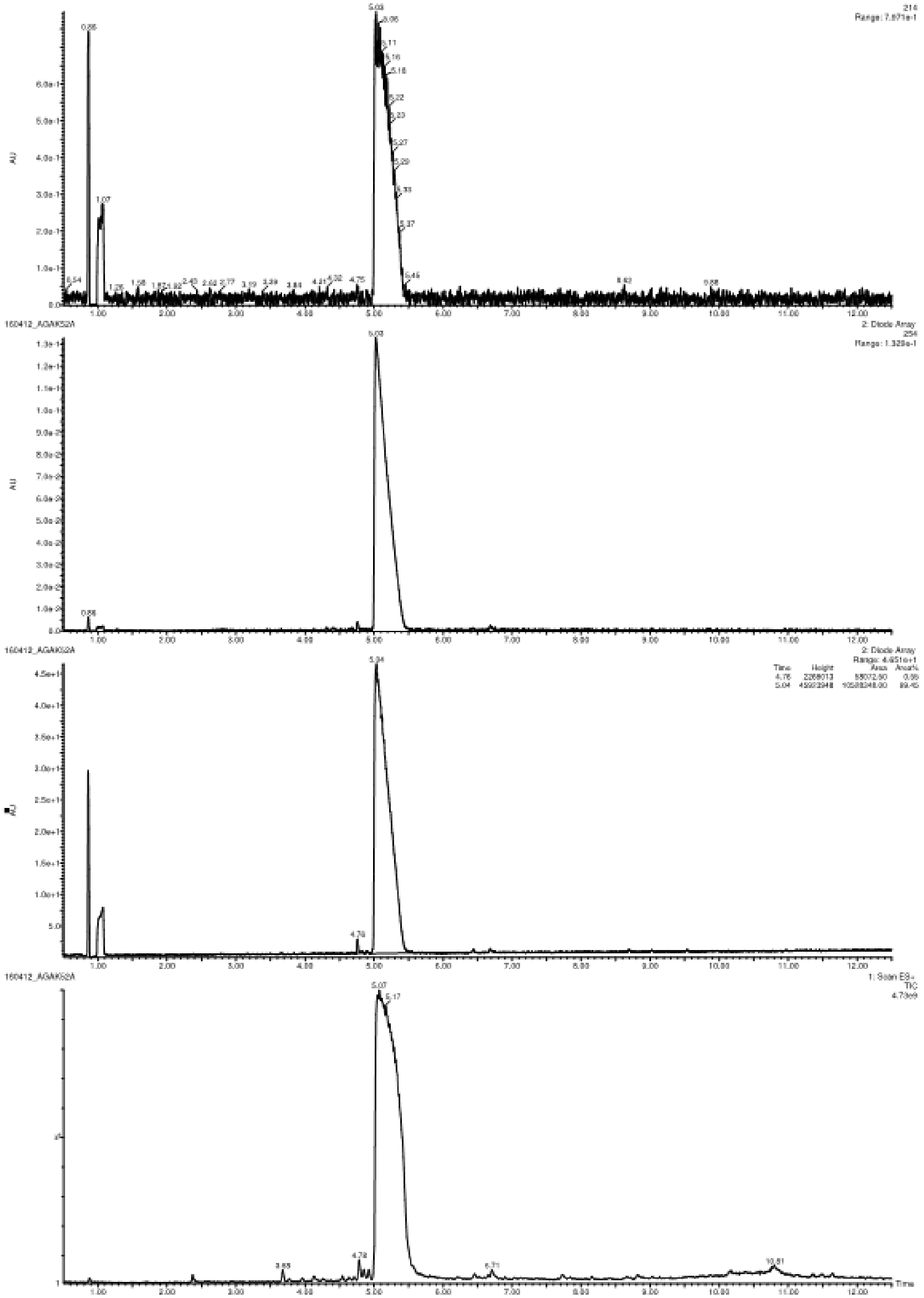

Figure S8. HPLC spectrum of compound 7 (99.45%).

b) Characterizations of compound 11

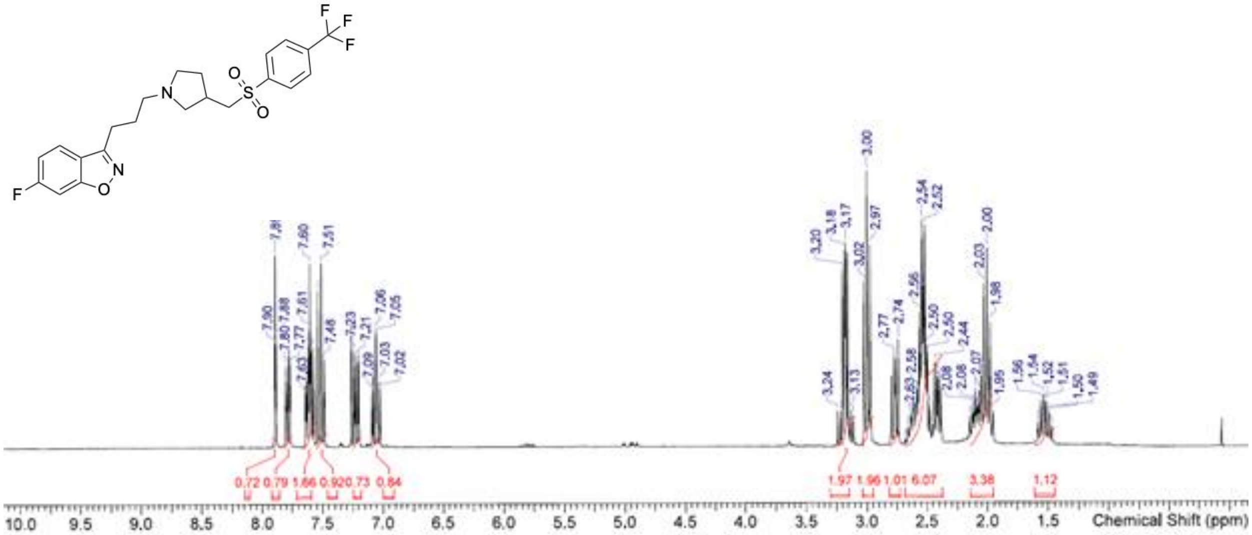

Figure S9. ^{1}H NMR (300 MHz, $CDCl_3$) spectrum of compound 11.

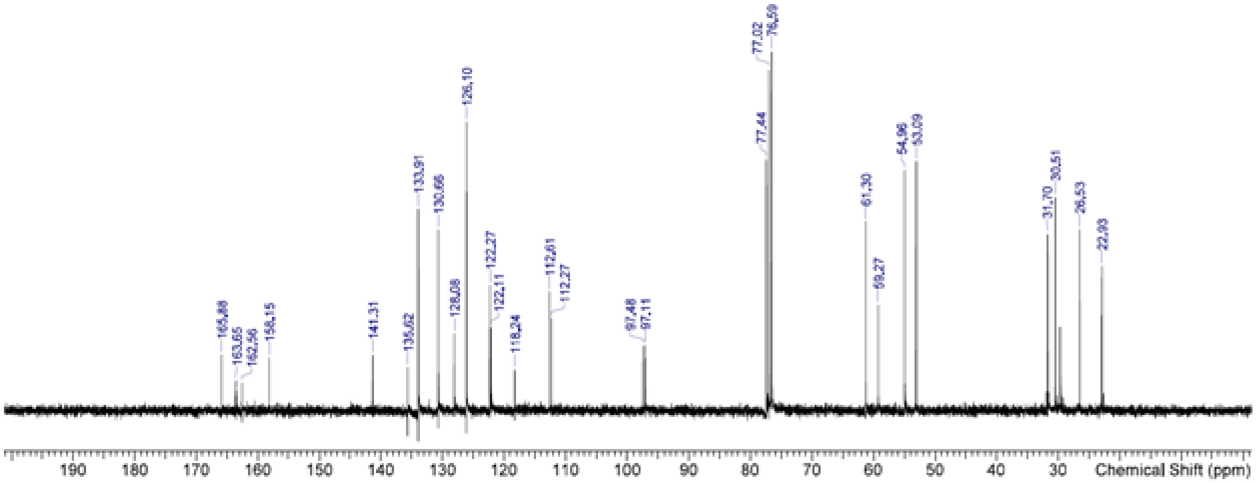

Figure S10. ^{13}C NMR (75 MHz, $CDCl_3$) spectrum of compound 11.

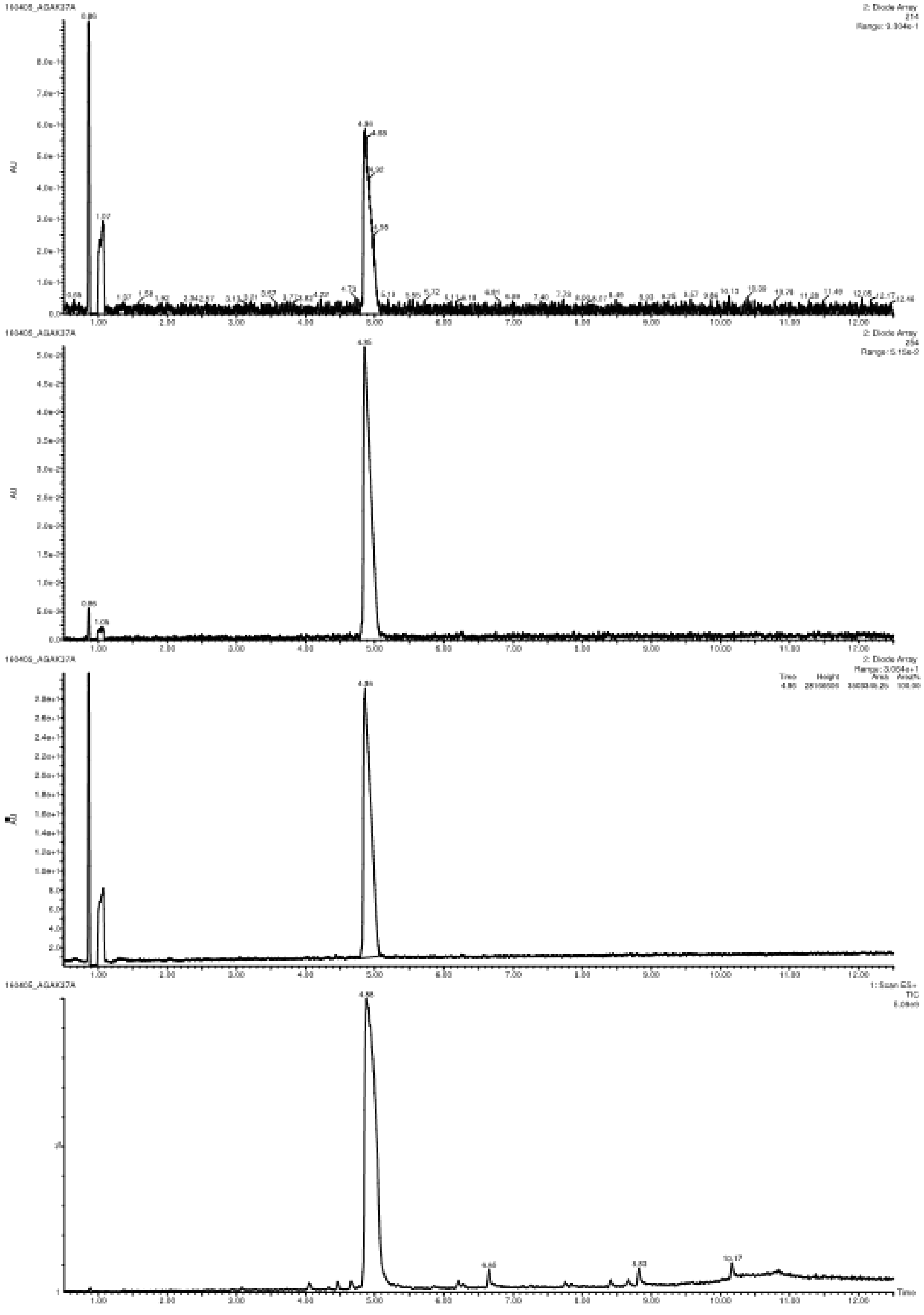

Figure S11. HPLC spectrum of compound 11 (100.00%).

c) Characterizations of compound 16

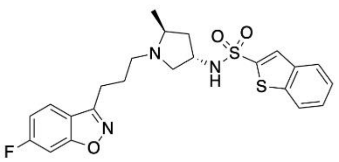

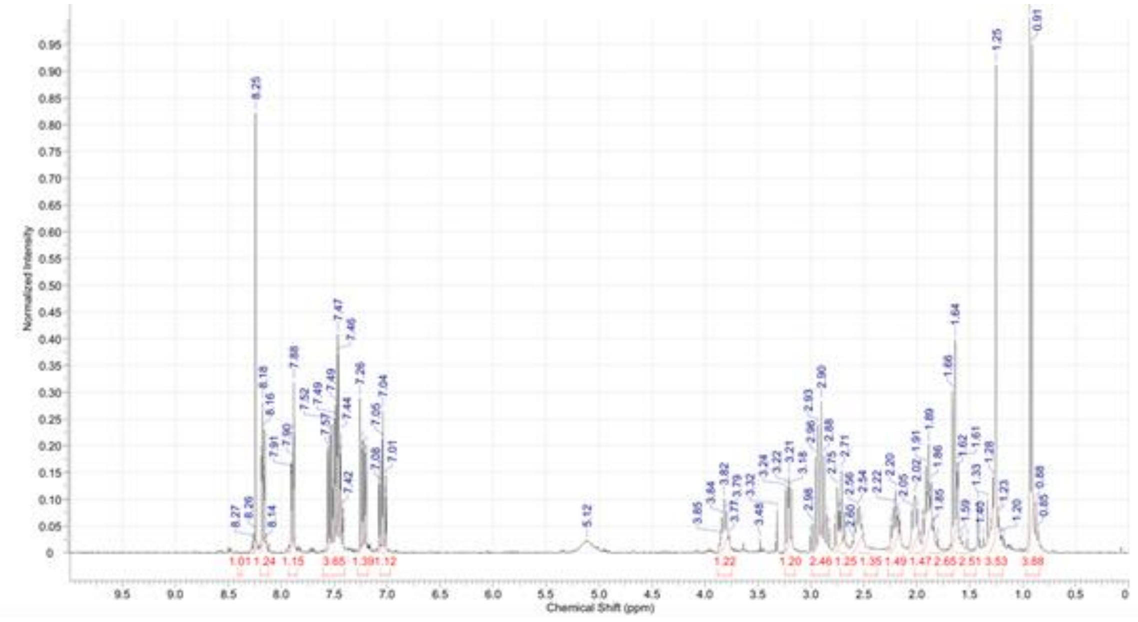

Figure S12. ^{1}H NMR (300 MHz, $CDCl_3$) spectrum of compound 16.

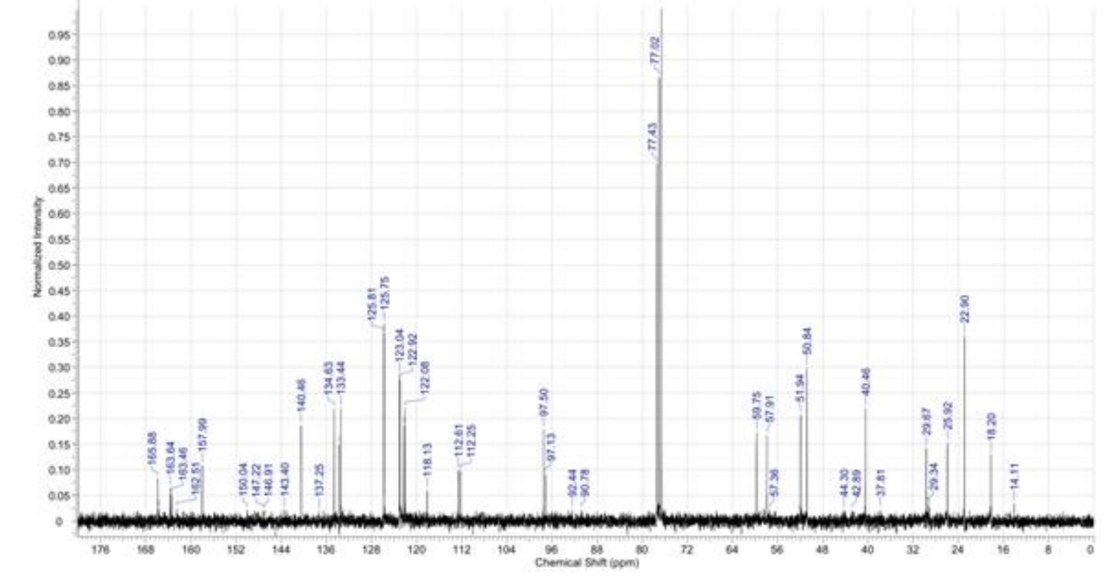

Figure S13. ^{13}C NMR (75 MHz, $CDCl_3$) spectrum of compound 16.

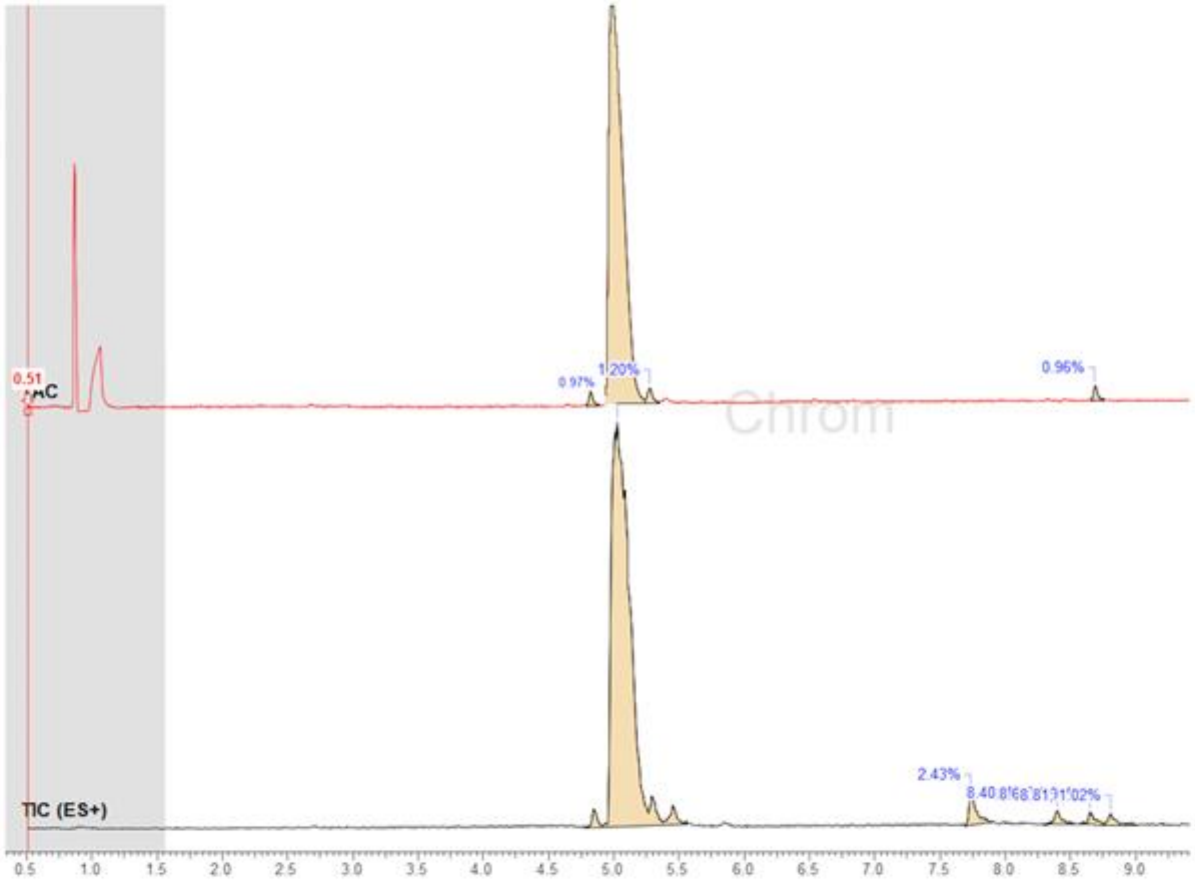

Figure S14. HPLC spectrum of compound 16 (96.87%).

6. Results of in vivo studies (compound 11)

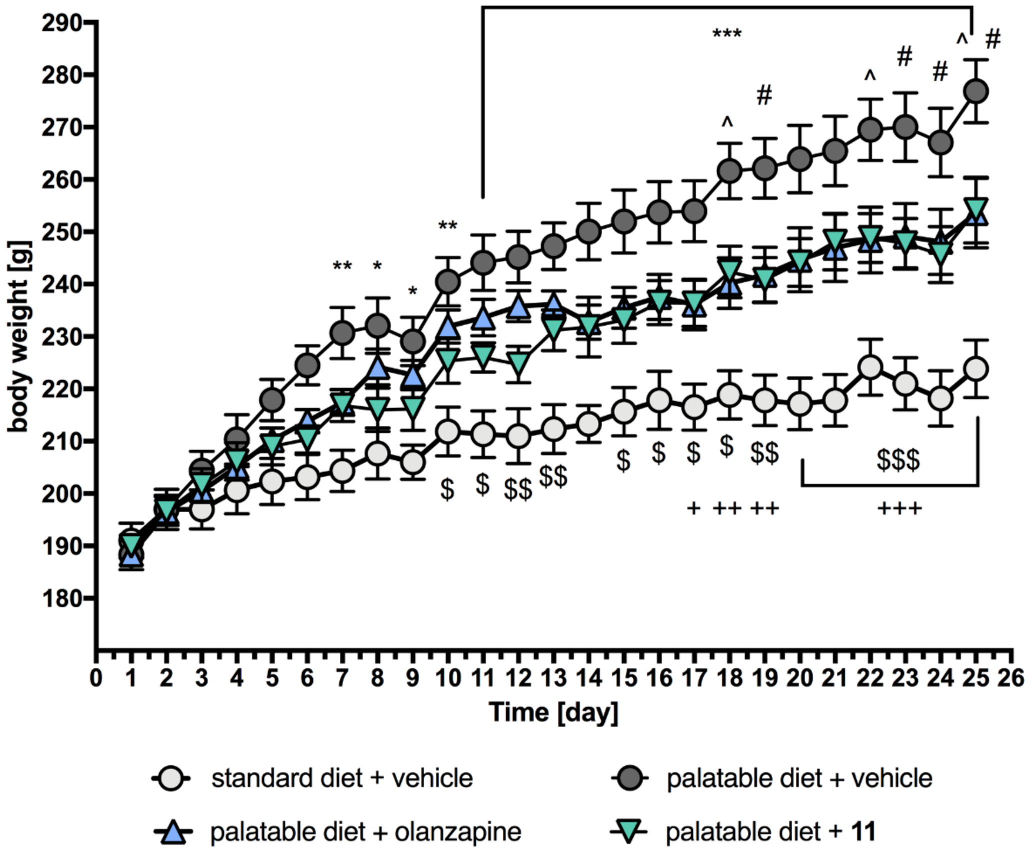

Figure S15. Effect of 11 and olanzapine on body weight

Statistical analysis: two-way ANOVA with repeated measure (Bonferroni *post hoc*); *p<0.05, **p<0.01, ***p<0.001 vehicle + standard diet vs vehicle + palatable diet; ^p<0.05 vehicle + palatable diet vs olanzapine + palatable diet; #p<0.05 vehicle + palatable diet vs 11 + palatable diet; +p<0.05, ++p<0.01, +++p<0.001 vehicle + standard diet vs 11 + palatable diet; $p<0.05, $$p<0.01, $$$p<0.001 vehicle + standard diet vs olanzapine + palatable diet; Mean +/- SEM, n= 6. The palatable diet induced significant increase in body weight comparing to animals fed with standard diet. Administration of 11 to animals that were fed with palatable diet significantly increased the body weigh comparing to animals fed with standard diet, however their body weight was significantly lower comparing to the control group (animals fed with palatable diet). Administration of olanzapine produced similar results.
